# Supplementary material for: IHR-PVS National Bridging Workshops, a tool to operationalize the collaboration between human and animal health while advancing sector-specific goals in countries
Source: PLoS One. 2021 Jun 1;16(6):e0245312. doi: 10.1371/journal.pone.0245312 (PMC8168895; doi:10.1371/journal.pone.0245312)

World Health Organization

WORLD ORGANISATION FOR ANIMAL HEALTH

Participant Handbook

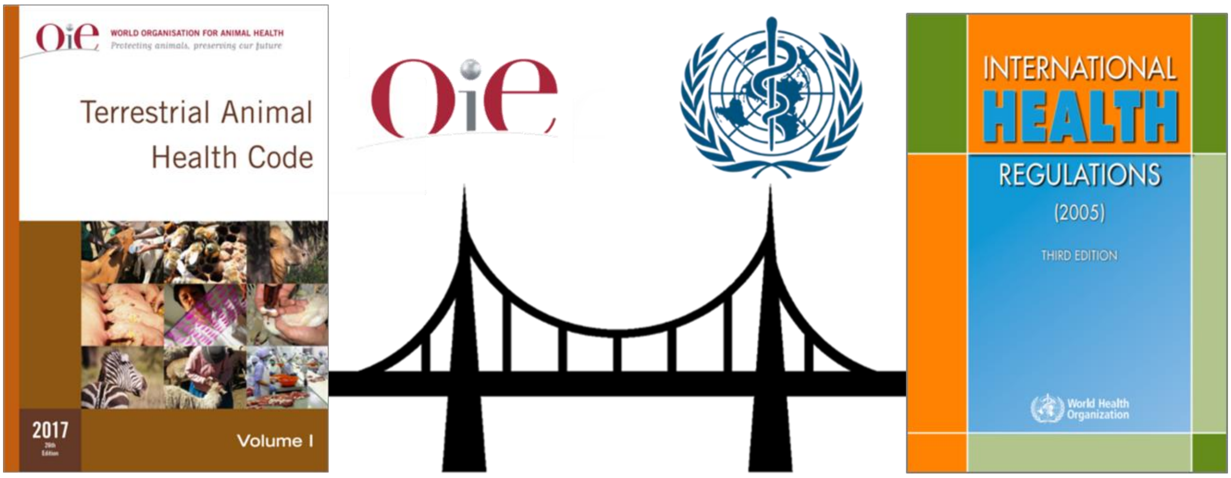

National IHR and PVS Pathway Bridging Workshop

The Road to One Health

(Version 6)

TABLE OF CONTENTS

TABLE OF CONTENTS 3

ABBREVIATIONS & ACRONYMS 4

INTRODUCTION 5

Background 5

Objectives of IHR-PVS National Bridging Workshops and expected outcomes 6

OVERALL PROCESS 7

SESSION 1: ONE HEALTH CONCEPT & NATIONAL PERSPECTIVES 9

Movie 1: Tripartite One Health collaboration & vision 9

Presentations: National public health & animal health services 13

Movie 2: Driving successful interactions 14

SESSION 2: THE ROAD TO ONE HEALTH – INTERACTIONS & GAPS 22

Exercise 1: Case studies & assessment of levels of collaboration 22

SESSION 3: BRIDGES ALONG THE ROAD TO ONE HEALTH 25

Movie 3: IHR monitoring & evaluation framework 25

Movie 4: PVS Pathway 32

Movie 5: IHR-PVS Bridging 39

IHR-PVS Matrix 43

Exercise 2: Mapping of gaps on the IHR-PVS matrix 47

Plenary: Discussion 47

SESSION 4: EXTRACTION OF ASSESSMENT RESULTS 48

Exercise 3: Extraction of assessment results 48

SESSION 5: JOINT ROAD PLANNING 50

Exercise 4: Identification of joint activities 50

SESSION 6: FINALIZATION OF THE JOINT ROAD-MAP 53

Exercise 5: Fine-tuning of the joint road-map 53

Exercise 6: World café 56

Exercise 7: Prioritization vote 56

SESSION 7: WAY FORWARD 57

EVALUATION OF THE WORKSHOP 58

ABBREVIATIONS & ACRONYMS

FAO	Food and Agriculture Organization of the United Nations
IHR	International Health Regulations (2005)
JEE	Joint External Evaluation
MEF	Monitoring and Evaluation Framework
NAPHS	National Action Plan for Health Security
OIE	World Organisation for Animal Health
PVS	Performance of Veterinary Services
WHO	World Health Organization

INTRODUCTION

BACKGROUND

The World Health Organization (WHO) and the World Organisation for Animal Health (OIE) are the two main international organizations responsible for proposing references and guidance for the public health and animal health sectors respectively. Working in close collaboration with FAO, WHO and OIE have been active promoters and implementers of an intersectoral collaborative approach among institutions and systems to prevent, detect, and control diseases among animals and humans. They have developed various frameworks, tools and guidance material to strengthen capacities at the national, regional and global levels.

- WHO Member States adopted a legally binding instrument, the International Health Regulations (IHR, revised in 2005), for the prevention and control of events that may constitute a public health emergency of international concern. Through these regulations, States Parties are required to **develop, strengthen and maintain** minimum national core public health capacities to **detect, assess, notify and respond to public health threats** and as such, should implement plans of action to develop and ensure that the core capacities required by the IHR are present and functioning throughout their territories. Various assessment and monitoring tools have been developed by WHO such as the **IHR Monitoring and Evaluation Framework (MEF)**, which includes *inter alias* the Annual Reporting Questionnaire for Monitoring Progress and the Joint External Evaluation (JEE) Tool.

- The OIE is the intergovernmental organization responsible for developing standards, guidelines and recommendations for animal health and zoonoses; these are mainly laid down in the OIE Terrestrial and Aquatic Animal Codes and Manuals. In order to achieve the sustainable improvement of national Veterinary Services' compliance with those standards, in particular on the quality of Veterinary Services. The OIE has developed the **Performance of Veterinary Services (PVS) Pathway**, which is composed of different tools to assist countries to objectively assess and address the main weaknesses of their Veterinary Services.

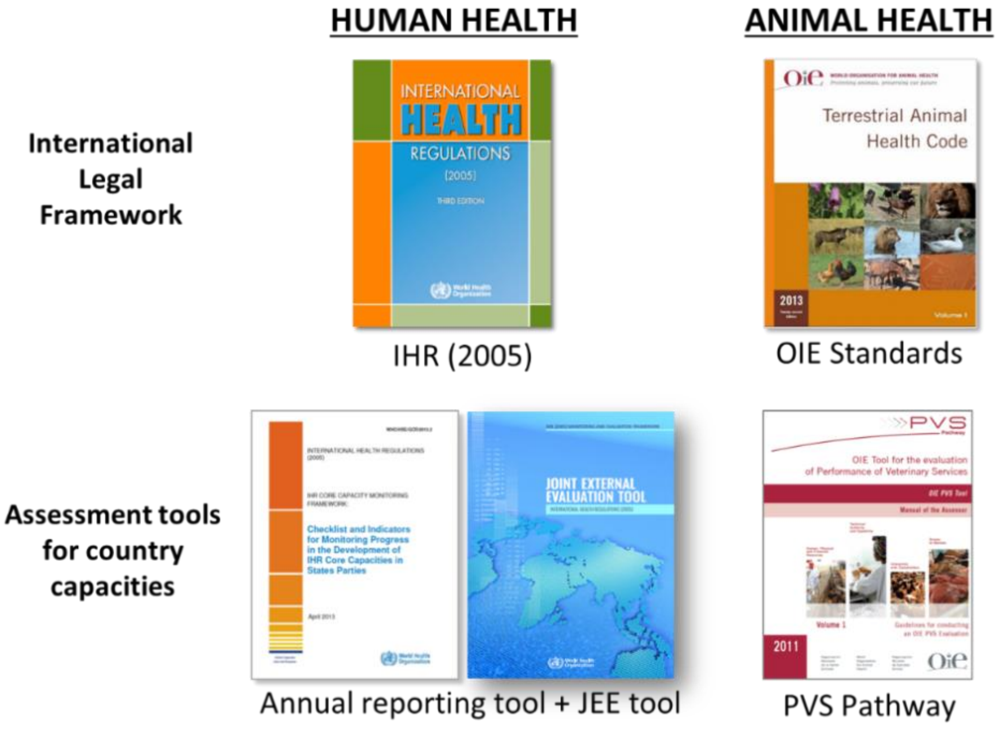

The use of WHO IHR monitoring tools and OIE PVS Pathway would result in a **detailed assessment of the existing forces and gaps**, with **better alignment of capacity building approach and strategies** at country level between the human and animal health sectors. The IHR-PVS National Bridging Workshops (NBW) enable countries to further explore possible overlapping areas addressed in the OIE and IHR capacity frameworks and develop, where relevant, appropriate bridges to facilitate coordination. A structured approach using user-friendly material, case studies and group exercises enables the identification of synergies, review of gaps and the definition of operational strategies to be used by policy makers for concerted corrective measures and strategic investments in national action plans for improved health security.

OBJECTIVES OF IHR-PVS NATIONAL BRIDGING WORKSHOPS AND EXPECTED OUTCOMES

The main objective of the NBW is to provide an opportunity to human and animal health services of hosting countries to review their current collaboration gaps in key technical areas and to develop a joint road-map of corrective measures and strategic investments to improve the work at the animal-human interface in the prevention, detection and control of zoonotic diseases.

Expected outcomes of the workshop:

- Increased awareness and understanding on the IHR-MEF and the OIE PVS Pathway, their differences and connections;
- Understanding of the contribution of the veterinary services in the implementation of the IHR (2005) and how the results of the PVS Pathway and IHR-MEF can be used to explore strategic planning;
- Diagnosis of current strengths and weaknesses in the collaboration between animal and human health services for key technical areas;
- Identification of practical next steps and activities for the development and implementation of joint national roadmap to strengthen collaboration and coordination.

OVERALL PROCESS

The workshop uses an interactive methodology and a structured approach with user-friendly material, case studies, group exercises, videos and facilitation techniques. The workshop is made of seven sessions that are structured in a step-by-step process from gap identification to action planning and validation of a joint roadmap for the improvement of the collaboration between the public health and animal health sectors.

Session 1 - Setting the scene: The first session sets the scene by providing background information on the One Health concept and the subsequent tripartite OIE-WHO-FAO collaboration. It is followed by comprehensive presentations from both Ministries on the national public and animal health services. A second documentary provides concrete worldwide examples of fruitful intersectoral collaboration, showing how the two sectors share a lot in terms of approaches, references and strategic views (total duration: **1h40**).

Session 2 - Identification of collaboration gaps: Participants are split in several working groups, each with a case study scenario. Participants discuss the management of zoonotic diseases, identify areas of convergence, evaluate the level of collaboration between the different sectors for key technical areas and identify the main gaps (total duration: **3h30**).

Session 3 - IHR-PVS tools and their bridging: The tools from both sectors (IHR MEF, JEE, PVS) are presented. Joint areas and activities identified for each case study are mapped onto a giant matrix consisting of the indicators of the IHR MEF and of the PVS Pathway. This process enables participants to visualize the gaps identified in each essential capacity and to distinguish disease-specific vs systemic gaps. This will also help determine which technical areas the following sessions will focus on (total duration: **2h30**).

Session 4 - Extraction of assessment results: Participants explore the improvement plans already proposed in the respective assessments (IHR annual reporting, JEE, PVS Evaluation, etc.), extract relevant sections and identify what can be synergized and improved jointly (total duration: **2h00**).

Session 5 - Joint road-planning: Results obtained from the case studies and from the assessment reports are used to develop a realistic and achievable road-map to improve the collaboration between the sectors (total duration: **2h30**).

Session 6 - Finalization of the joint road-map: Through a world-café exercise followed by a plenary discussion, participants contribute to all technical areas to consolidate the joint-road map by making sure it is harmonized, concrete and achievable (total duration: **3h00**).

Session 7 - Way forward: the last session draws the way forward by identifying the next steps and by linking the developed road-map with other mandated plans such as the National Action Plan for Health Security. This is also where any need from the country can be addressed. This will depend greatly on the current status of the country in terms of IHR-MEF and on their level of One Health capacity.

The workshop uses a road analogy (The Road to One Health), and its process can be summarized with the following figure:

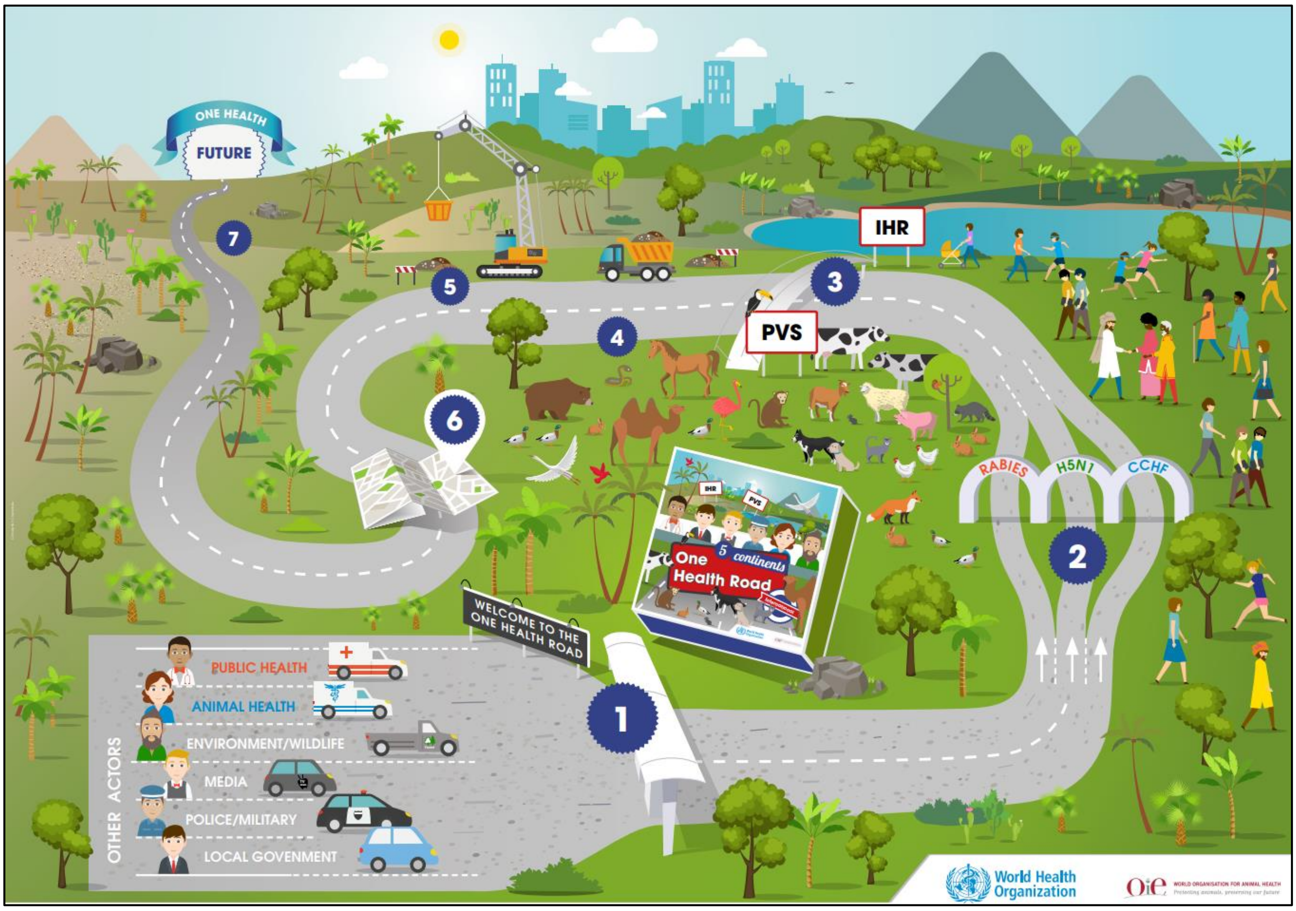

SESSION 1: ONE HEALTH CONCEPT & NATIONAL PERSPECTIVES

Objective: Session 1 sets the scene of the workshop by providing background information on the One Health concept and the subsequent tripartite OIE-WHO-FAO collaboration. It is followed by comprehensive presentations from both Ministries on the national public and animal health services. A second documentary provides concrete worldwide examples of fruitful intersectoral collaboration, showing how the two sectors share a lot in terms of approaches, references and strategic views.

MOVIE 1: TRIPARTITE ONE HEALTH COLLABORATION & VISION

This first documentary video introduces the One Health Concept, its history, rationale and purpose and how it became an international paradigm. The video also introduces the workshop in the global and national context by providing information on the tripartite collaboration between WHO, OIE and FAO.

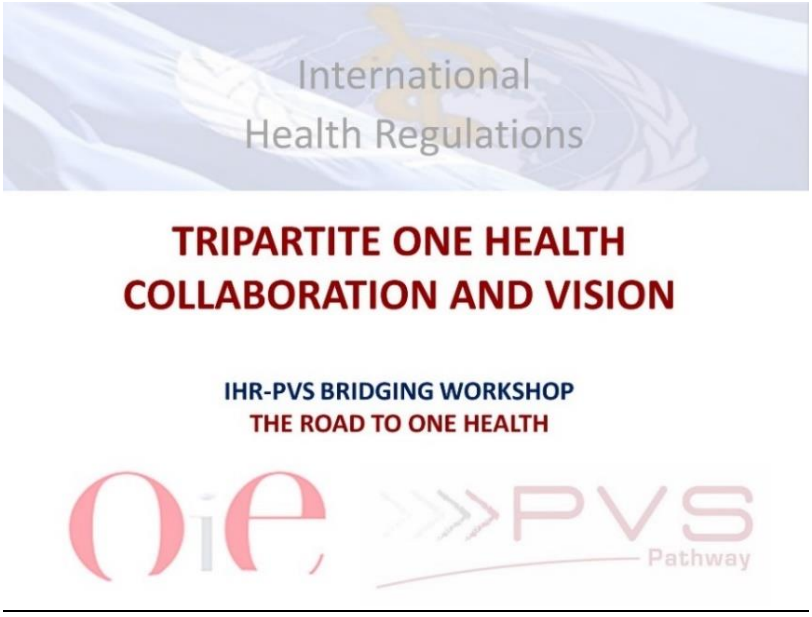

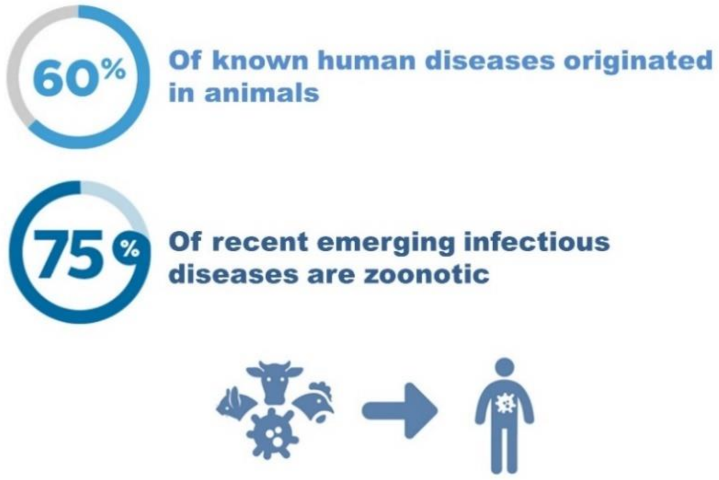

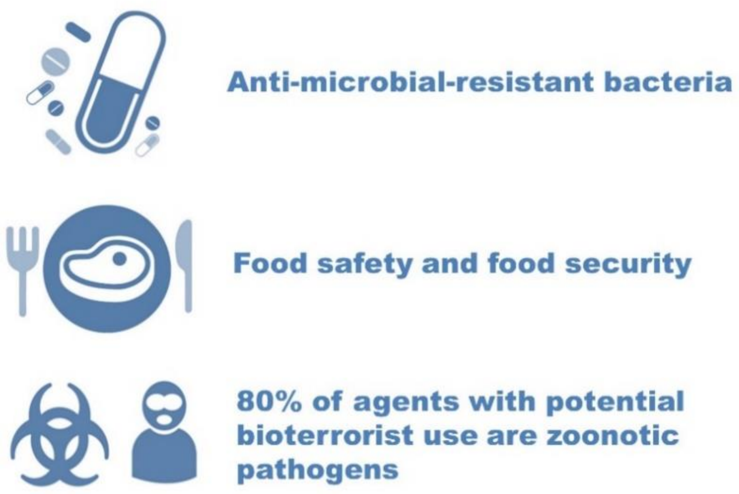

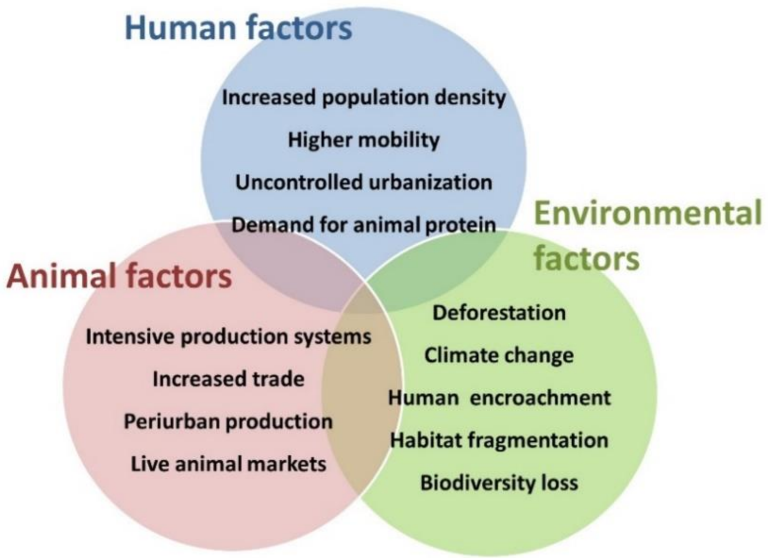

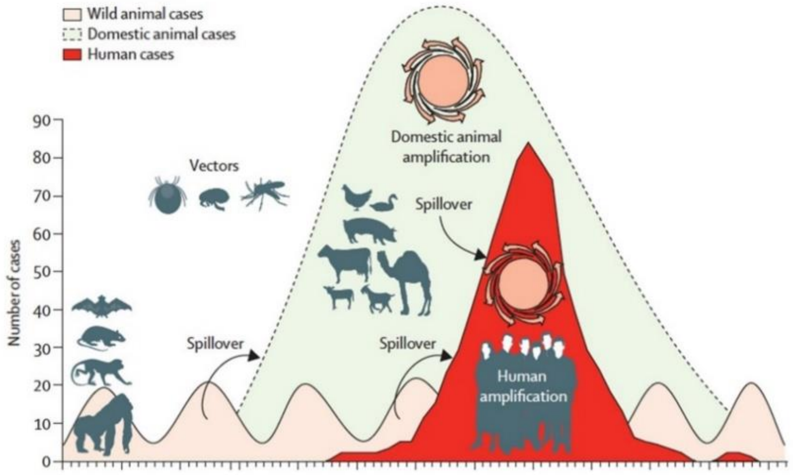

From P.Formenty, in Karesk and coll, 2012, Ecology of Zoonosis, The Lancet

WILDLIFE CONSERVATION SOCIETY

Key principles of Manhattan

New York, September 29, 2004

- *"We are in an era of "One World, One Health" and we must devise adaptive, forward-looking and multidisciplinary solutions to the challenges that undoubtedly lie ahead."*
- *"It is clear that no one discipline or sector of society has enough knowledge and resources to prevent the emergence or resurgence of diseases in today's globalized world."*
- *"Only by breaking down the barriers among agencies, individuals, specialties and sectors can we unleash the innovation and expertise needed to meet the many serious challenges to the health of people, domestic animals, and wildlife and to the integrity of ecosystems. "*

2004

OIE, unicef, FAO FIAT PANIS, World Health Organization …

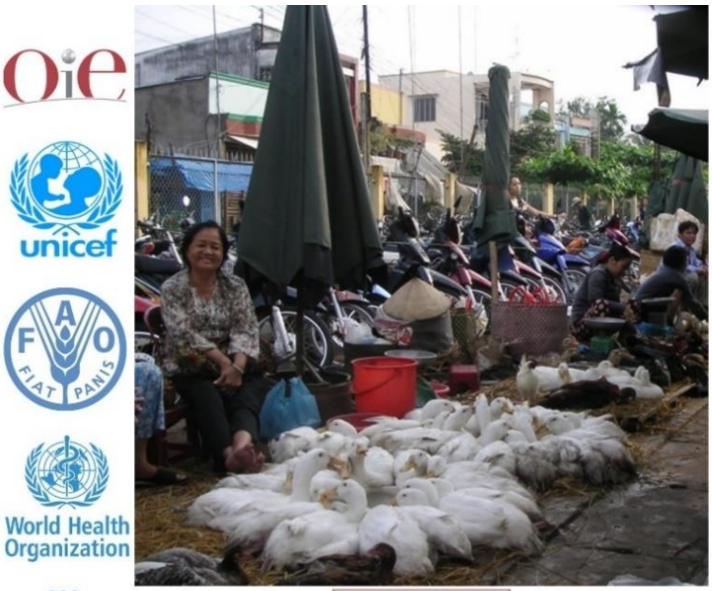

THE WORLD BANK, ADB Asian Development Bank, Banque Africaine de Développement / African Development Bank …

2004-2008

One Health Joint Strategic Framework

Contributing to
One World, One Health*

A Strategic Framework for Reducing Risks of Infectious Diseases at the Animal–Human–Ecosystems Interface

14 October 2008

Consultation Document

Produced by:

FAO, OIE, World Health Organization, UN System Influenza Coordination, unicef, THE WORLD BANK

Five strategic elements:

- Building **robust public and animal health systems** compliant with the **WHO IHR (2005)** and **OIE international standards**
- **Improving** national and international emergency **response capabilities**
- Focusing on **developing economies** for a **global benefit**
- Collaboration **across sectors and disciplines**
- Developing **specific disease control programmes**

2008

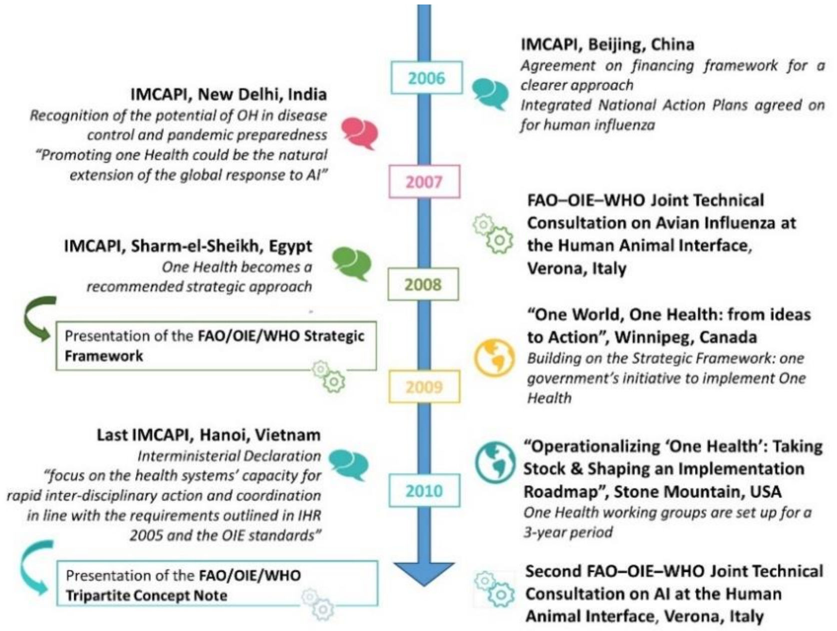

Tripartite Concept Note

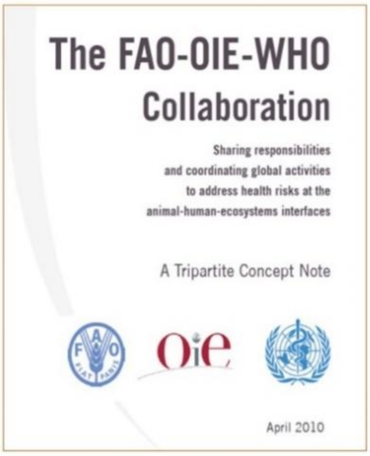
The FAO-OIE-WHO Collaboration

Sharing responsibilities and coordinating global activities to address health risks at the animal-human-ecosystems interfaces

A Tripartite Concept Note

April 2010

- Prevention and control of emerging infectious diseases is a **global public good**
- Robust public and animal health systems based on **good governance**
- National capacity to implement human and animal health **international standards**

2010

KEY MESSAGES

- **The 2 sectors share a lot** in terms of approaches, references, and strategic visions.
- These visions can be translated into **legal / regulatory / operational frameworks** that can be **used to put intersectoral collaboration into practice** at the **country level.**
- WHO and OIE are promoting the **compliance to the IHR (2005) and the OIE Terrestrial and Aquatic Codes** and support their Member Countries in **assessing existing strengths and gaps at the animal-human interface**, and in developing **roadmaps** aimed at improving intersectoral collaboration and operational capacities.

PRESENTATIONS: NATIONAL PUBLIC HEALTH & ANIMAL HEALTH SERVICES

Notes

MOVIE 2: DRIVING SUCCESSFUL INTERACTIONS

This documentary provides participants with concrete worldwide examples of intersectoral collaboration in addressing health issues at the human-animal interface. The movie explains that, although there is almost always an inter-ministerial committee, this does not guaranty efficiency of operations at field level. Using the model developed for Rift Valley Fever, an example of a sub-committee framework to help bridge the two sectors at the technical level for all key technical domains is proposed.

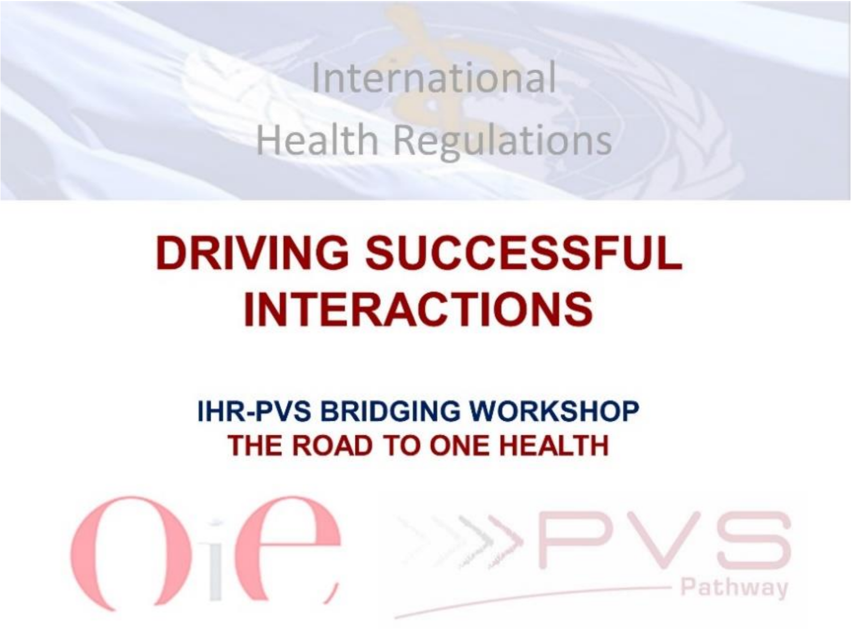

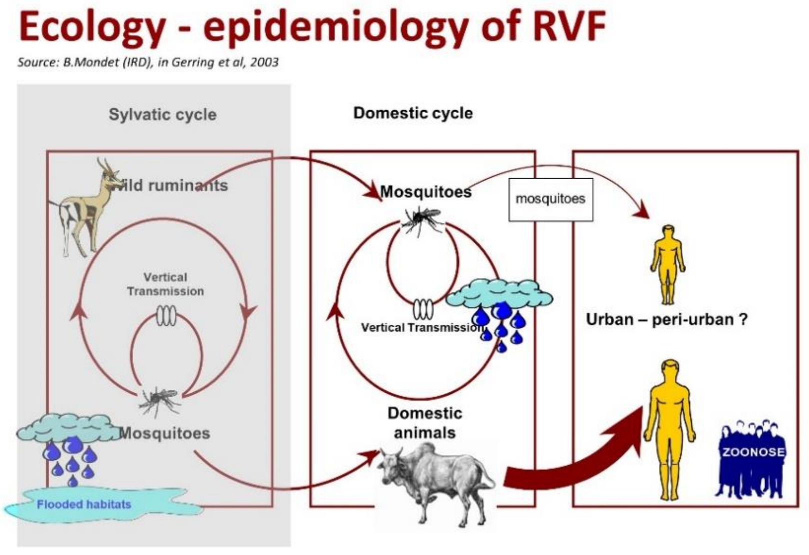

Emergence, amplification and spread

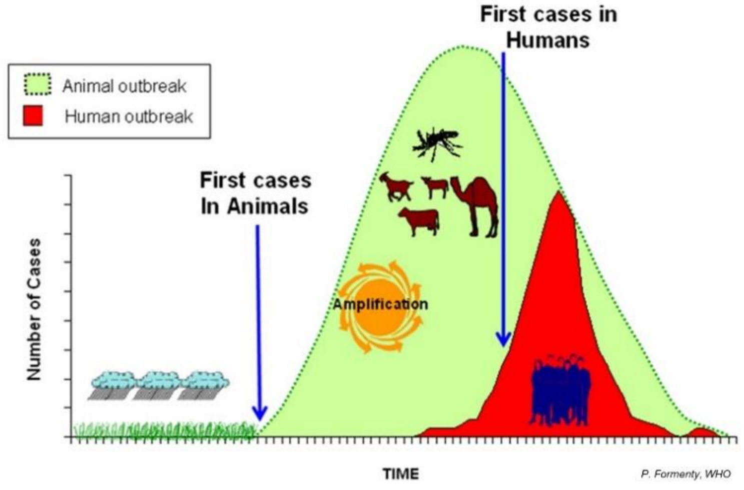

RVF distribution and major outbreaks

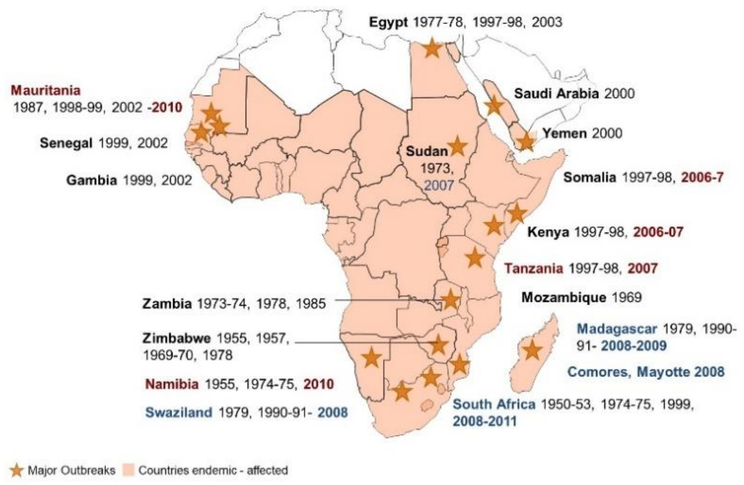

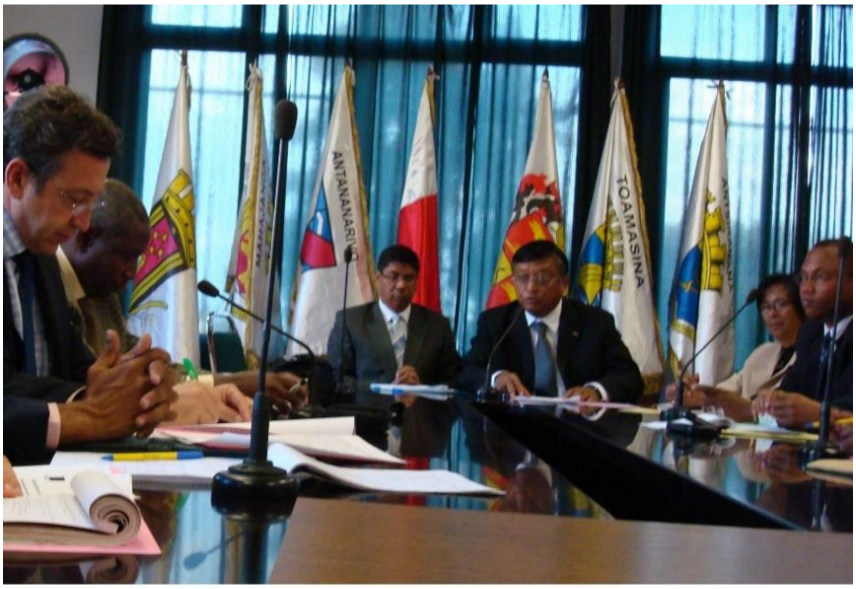

Madagascar, 2008 – Inter-Ministerial Coordination Taskforce

Coordination mechanism for response

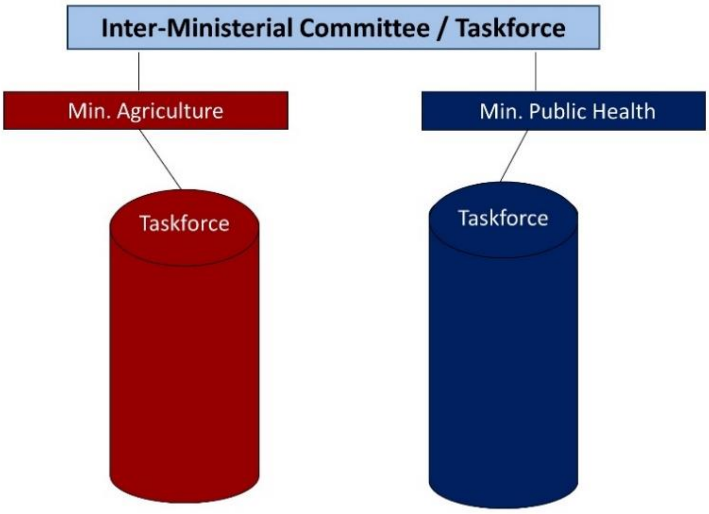

Coordination mechanism for response

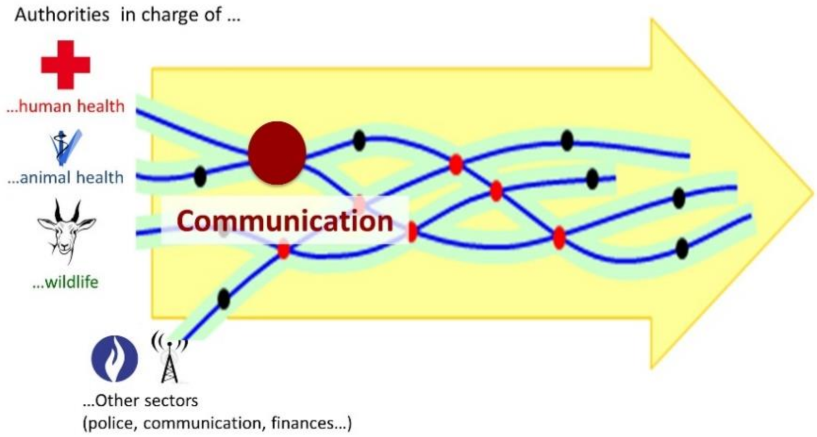

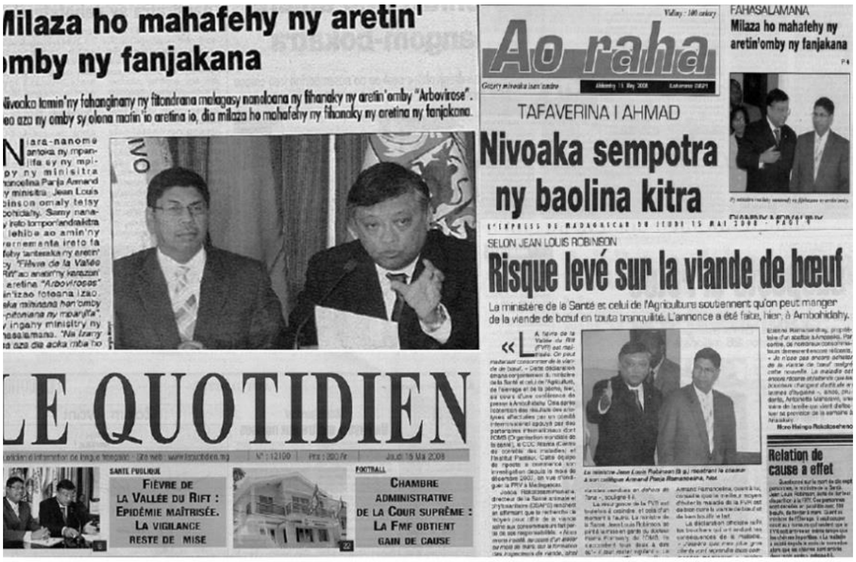

Madagascar, 2008 – Joint communication & Partnership with media

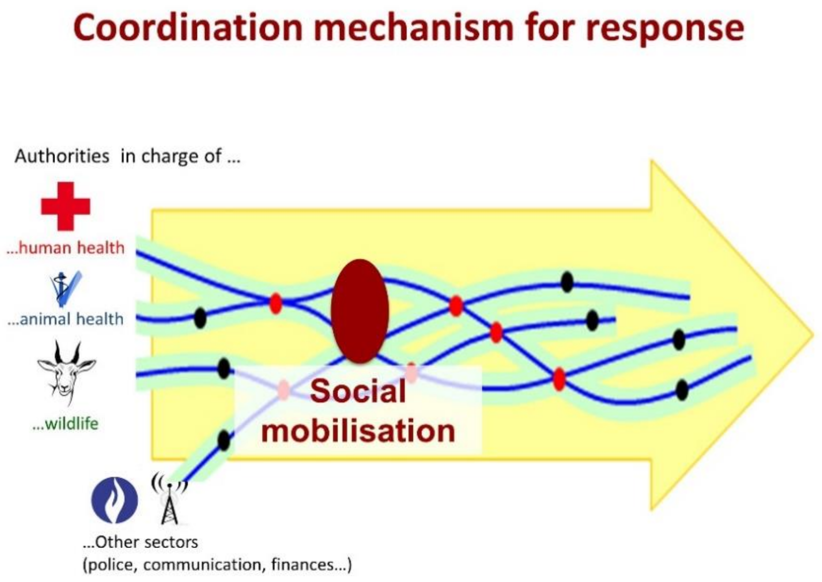

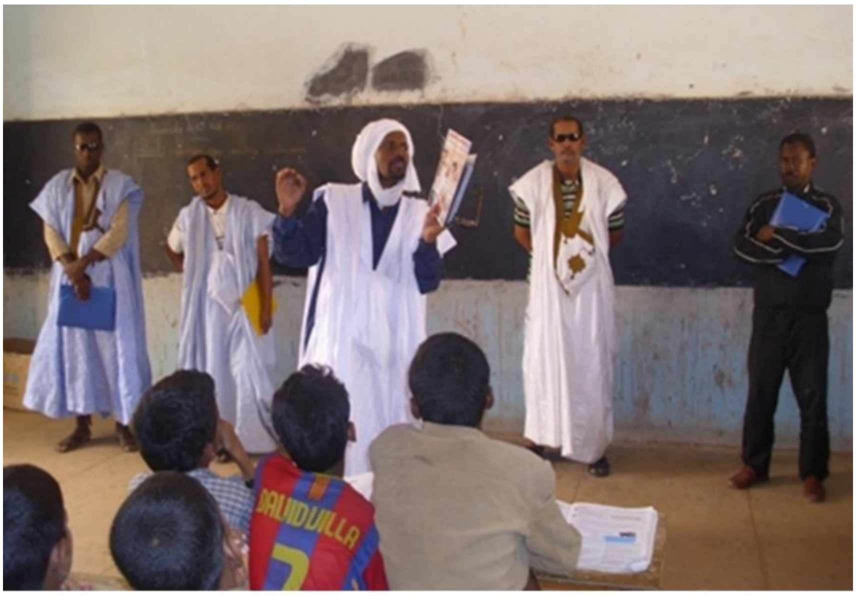

Adrar, Mauritania, 2011 - Social mobilization

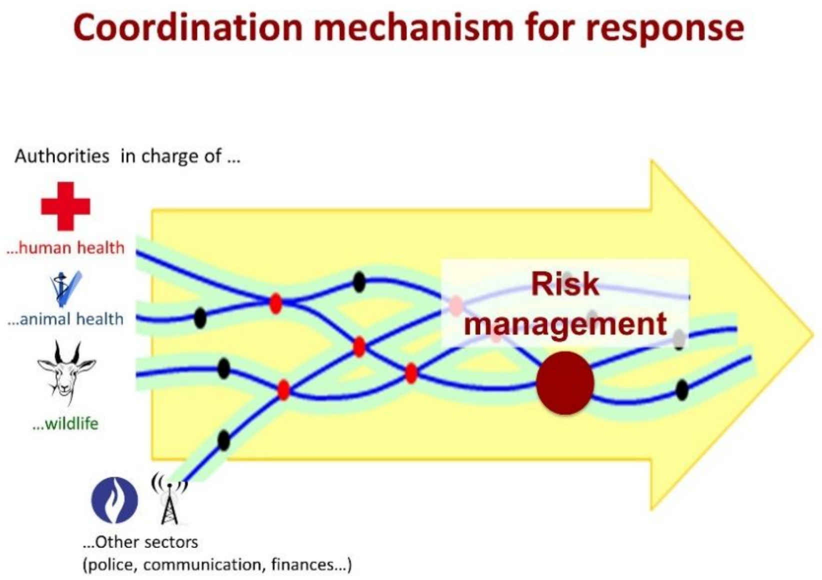

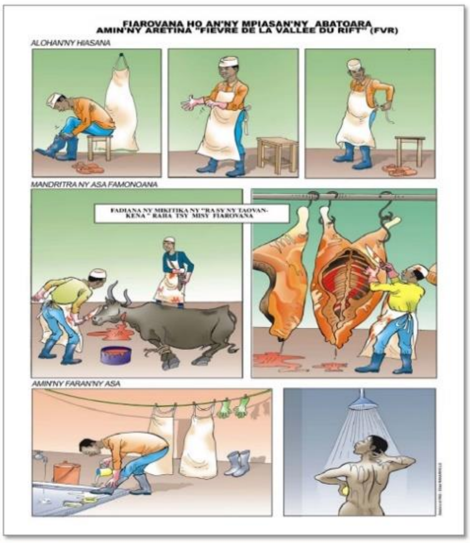

Promote practices that restrict transmission and source of infection

The social and cultural aspects are usually underestimated or neglected when they are key.

The support of medical anthropology is highly beneficial.

Madagascar, 2008 - Protect population at risk

Coordination mechanism for response

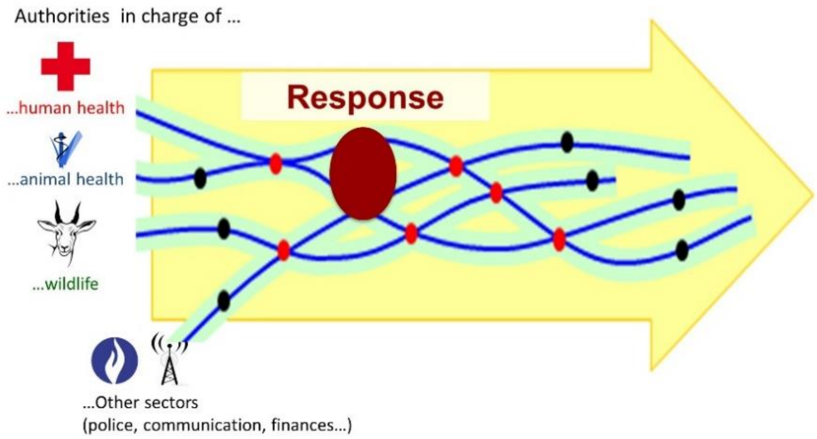

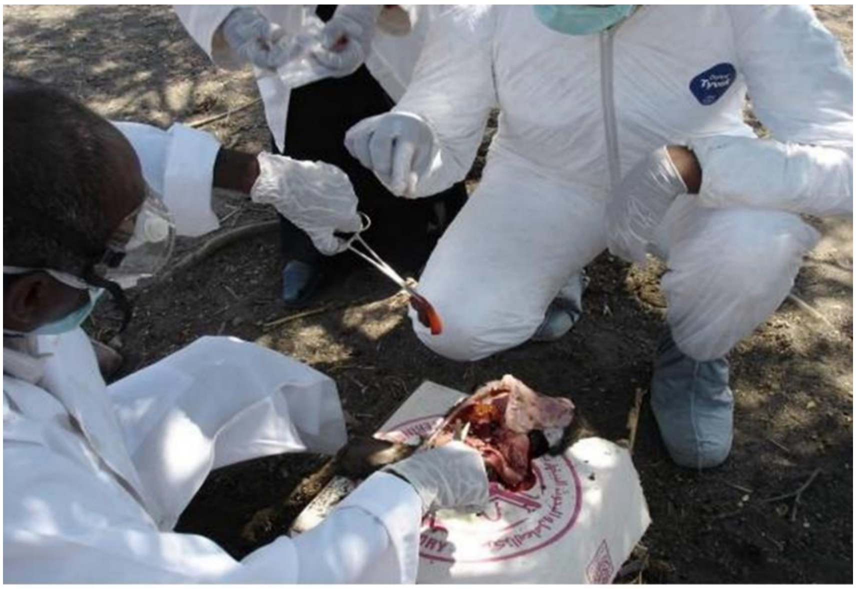

White Nile, Sudan, 2007 – Joint investigation

Coordination mechanism for response

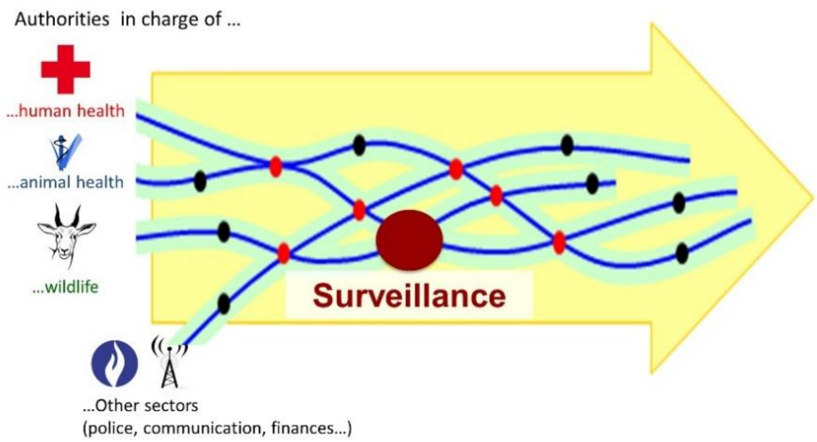

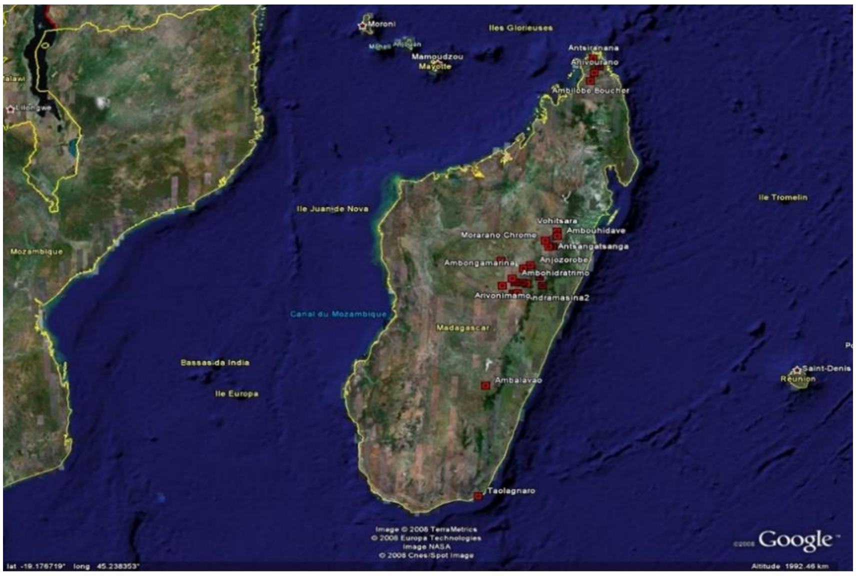

Madagascar, 2008 - Active and passive surveillance

Prevalence IgG in Cattle

Prevalence IgM in Cattle

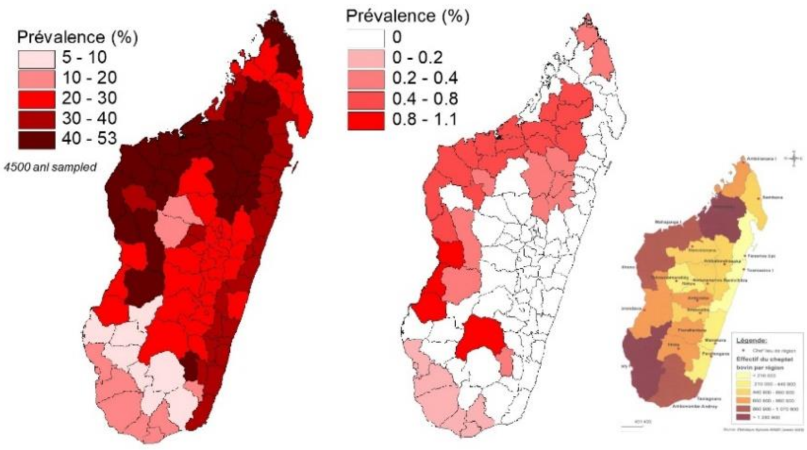

Madagascar, 2008 - Active and passive surveillance

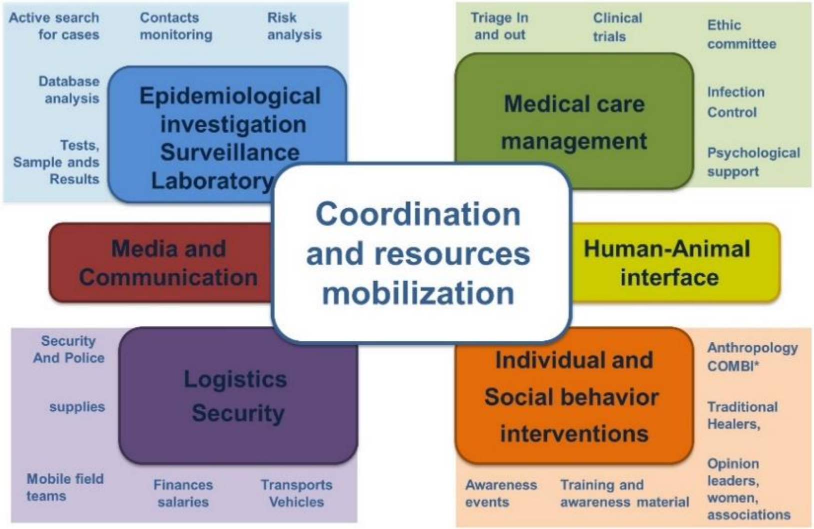

Multidisciplinary strategy for controlling a RVF outbreak

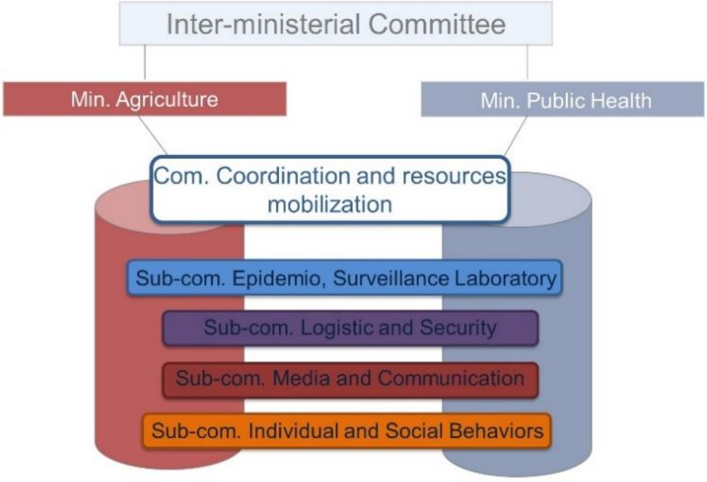

Multidisciplinary strategy for controlling a RVF outbreak

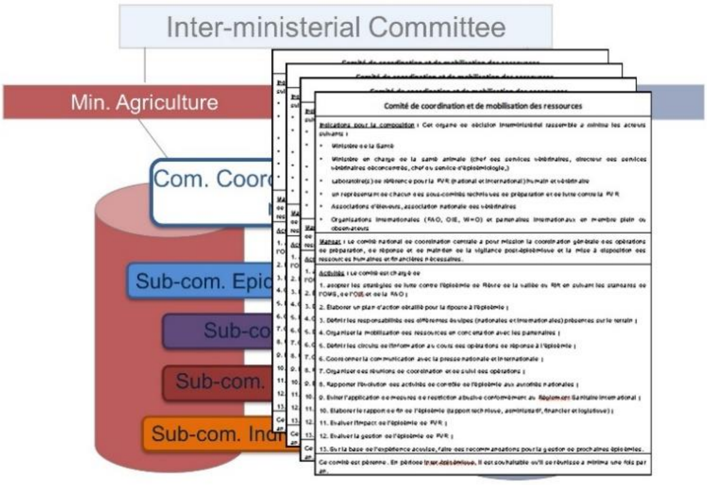

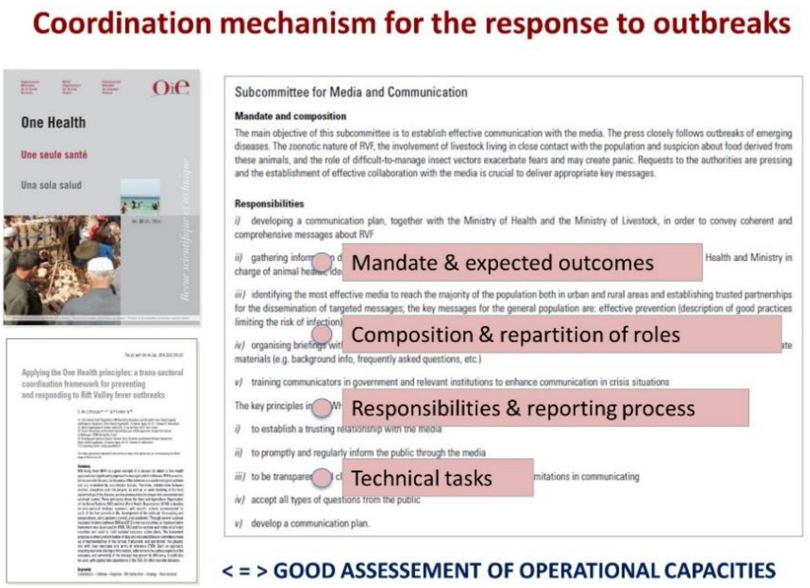

KEY MESSAGES

- During the course of an outbreak, efficient **ad-hoc collaboration** can be obtained when needed.
- With better **preparedness** in peace time, much more could be done to break the silos at the human-animal interface.
- The collaboration should be **organized**, with a strong leadership, and clear repartition of tasks, roles and activities.
- **Pooling of resources and expertise** would be highly beneficial, should the **capacities – and gaps –** in each sector and at the interface be assessed.

Expected outcomes of Session 1:

- Intersectoral collaboration between animal and human health sectors happen, but mainly (only?) during outbreaks; with a better preparedness, much more could be done at the human-animal interface.
- The two sectors have common concern and challenges and conduct similar activities. Competencies exist and can be pooled. This need to be organized though a collaborative approach;
- WHO, OIE and FAO are active promoters of One Health and can provide technical assistance to countries to help enhance inter-sectoral collaboration at the central, local and technical levels.

SESSION 2: THE ROAD TO ONE HEALTH – INTERACTIONS & GAPS

Objective: Discuss the management of zoonotic diseases, identify areas of convergence, evaluate the level of collaboration between the different sectors for key technical areas and identify the main gaps.

EXERCISE 1: CASE STUDIES & ASSESSMENT OF LEVELS OF COLLABORATION

Process

Using experience from previous outbreaks of zoonotic diseases, discuss on how you would have realistically managed these events, and evaluate the level of collaboration between the veterinary and the public health services for 15 key technical areas: coordination, investigation, surveillance, communication, etc. These activities/areas of collaboration are represented by the color-coded technical area cards.

1. Identify a chairman, a rapporteur and a time-keeper for your group
2. Read the scenario and these instructions carefully
3. Discuss on past experiences in the management of similar situations
4. Evaluate, for all 15 technical areas, the current level of collaboration using the color-coded cards:
    - Very good level of collaboration: **GREEN** card
    - Some level of collaboration: **ORANGE** card
    - Insufficient level of collaboration: **RED** card
5. Put the selected cards on the road-lane arrow and link them to all actors involved using the marker pen
6. Fill the report-sheet for each technical card by ticking the chosen colour and writing the one or two key points justifying this choice. These report sheets will be used by other groups in Session 5, therefore please make sure to write in a clear and intelligible manner.

Example of expected results

-An intersectoral committee with actors from both services exists and meets both regularly and on an ad-hoc basis when required. Coordination of the response to the outbreak is done jointly at the central level → **Green card** for **'Coordination at high level'**.

-Communication messages are sometimes developed jointly by both sectors but communication plans are not aligned or shared → **Orange card for 'Communication with media'**.

-Each sector carries out its own surveillance and results are rarely shared → **Red card** for **'Surveillance'**.

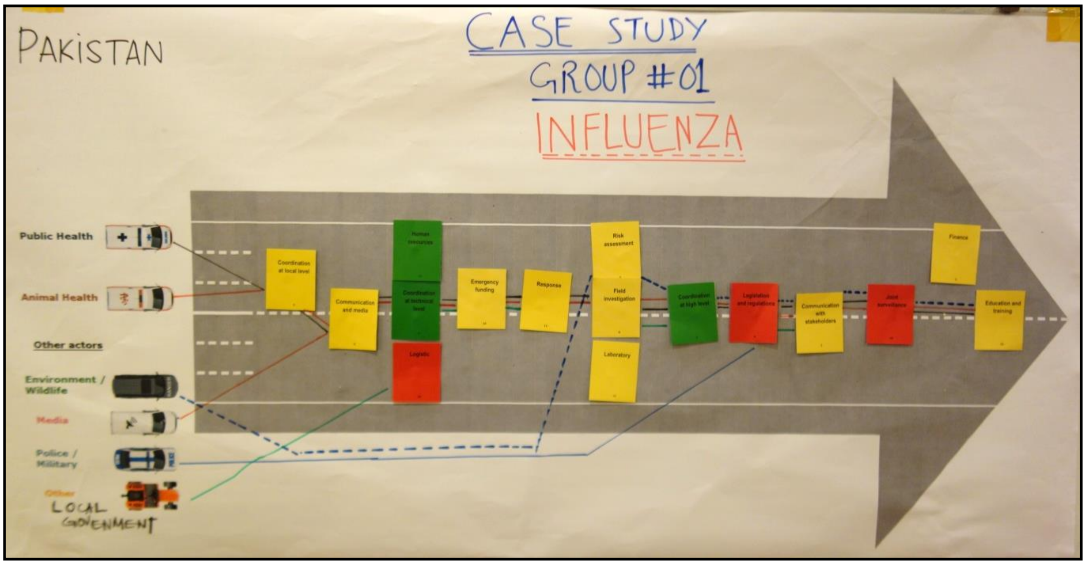

Answers to frequently asked questions or common mistakes

-The arrow does not necessarily represent a timeline;

-There is no required order for the cards. The location of the card on the arrow does not matter either, only its colour and its link to involved actors is important;

-Only one colour for each card should be selected;

-A red card does not necessarily mean that there is absolutely nothing in place, just like a green card does not necessarily mean that everything is perfect;

-The purpose of the scenario is only to set the context for the discussions, do not be too strict with the details and feel free to drift away from the storyline if needed;

-Examples at the back of the cards are only for guidance. They are not check-lists required to get a green card.

Important: It is essential to understand that you must evaluate the level of **collaboration**, and not the level of capacity of each sector!

Material and documents

Case study scenario

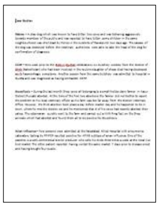

Deck of technical cards

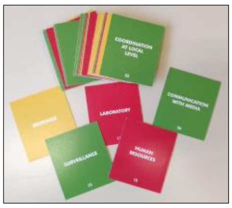

Road-lane arrow poster

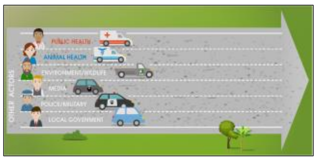

Black marker pen

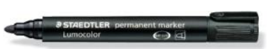

Blue-tack

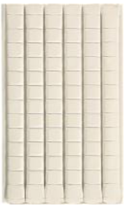

Report sheet

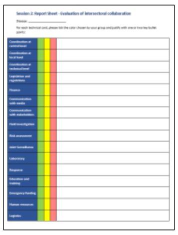

Your results

Disease: ________________________

Level of collaboration (circle your group's result):

Coordination at high level:	GREEN	ORANGE	RED
Coordination at local level:	GREEN	ORANGE	RED
Coordination at technical level:	GREEN	ORANGE	RED
Legislation and regulation:	GREEN	ORANGE	RED
Finance:	GREEN	ORANGE	RED
Communication and media:	GREEN	ORANGE	RED
Communication with stakeholders:	GREEN	ORANGE	RED
Field investigation:	GREEN	ORANGE	RED
Risk assessment:	GREEN	ORANGE	RED
Joint surveillance:	GREEN	ORANGE	RED
Laboratory:	GREEN	ORANGE	RED
Response:	GREEN	ORANGE	RED
Education and training:	GREEN	ORANGE	RED
Emergency funding:	GREEN	ORANGE	RED
Human resources:	GREEN	ORANGE	RED

Most important gaps identified in the collaboration:

Expected outcomes of Session 2:

- Areas of collaboration are identified, and joint activities discussed.
- Level of collaboration between the two sectors for 15 key technical areas is assessed
- The main gaps in the collaboration are identified.

SESSION 3: BRIDGES ALONG THE ROAD TO ONE HEALTH

Objective: Session 3 presents the tools from both sectors (IHR MEF, JEE, PVS) and uses an interactive approach to map the joint areas and activities identified for each case study onto a giant matrix consisting of the indicators of the IHR MEF and of the PVS Pathway.

This process will enable you to visualize the main gaps identified in each essential capacity and to distinguish disease-specific vs systemic gaps. This will also help identify which technical areas the following sessions should focus on.

MOVIE 3: IHR MONITORING & EVALUATION FRAMEWORK

This documentary video presents the International Health Regulations from the initial conception to the recent revisions. It introduces the Monitoring and Evaluation Framework with a special focus on the annual reporting of capacities and the Joint External Evaluation.

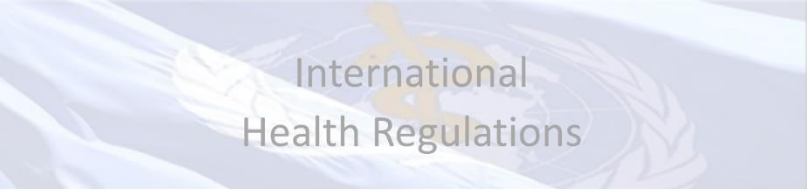

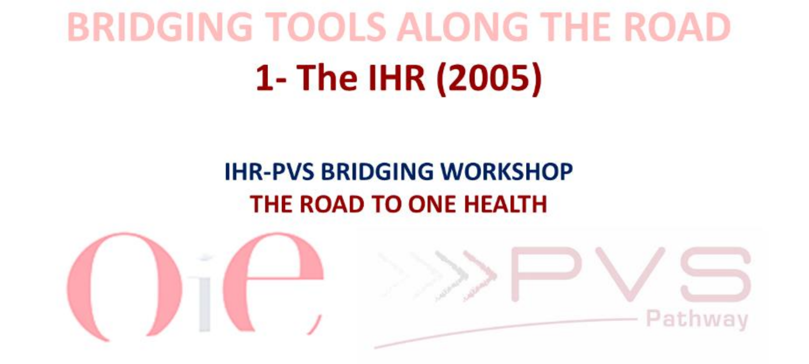

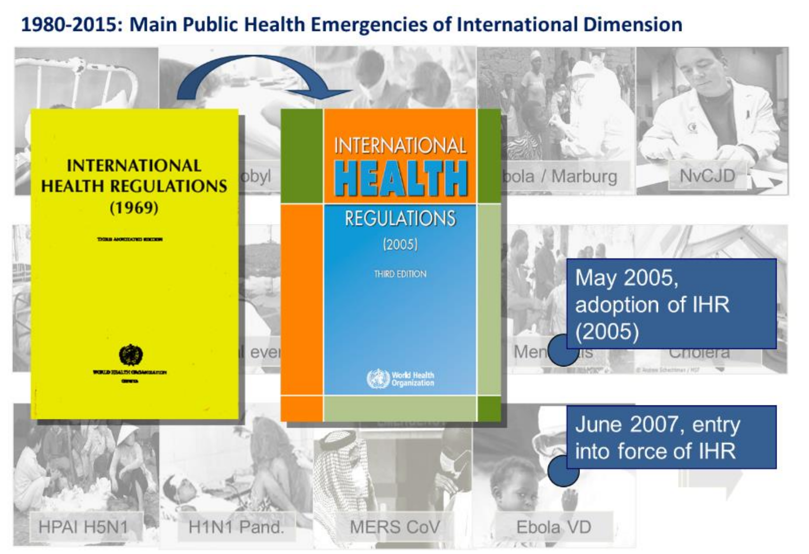

1980-2015: Main Public Health Emergencies of International Dimension

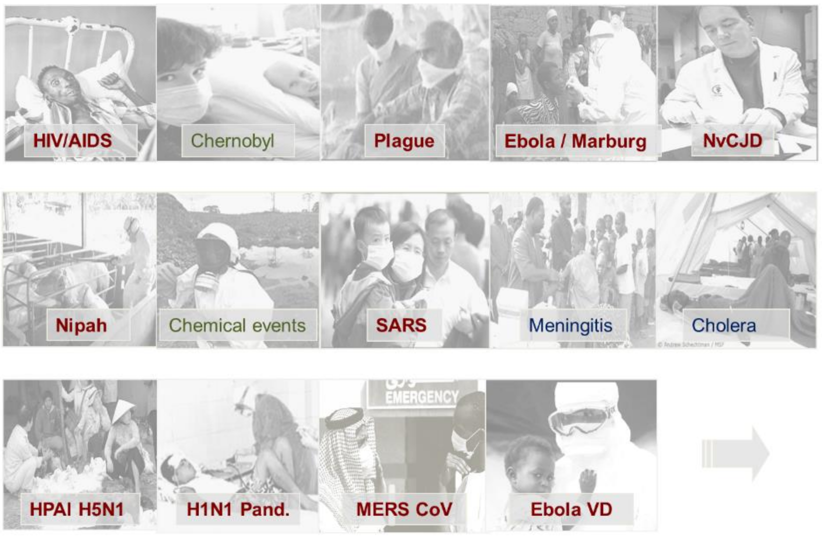

Zoonotic or of animal origin – **Human only** – non infectious

In 2005, the 58th World Health Assembly adopted the revised International Health Regulations (IHR)

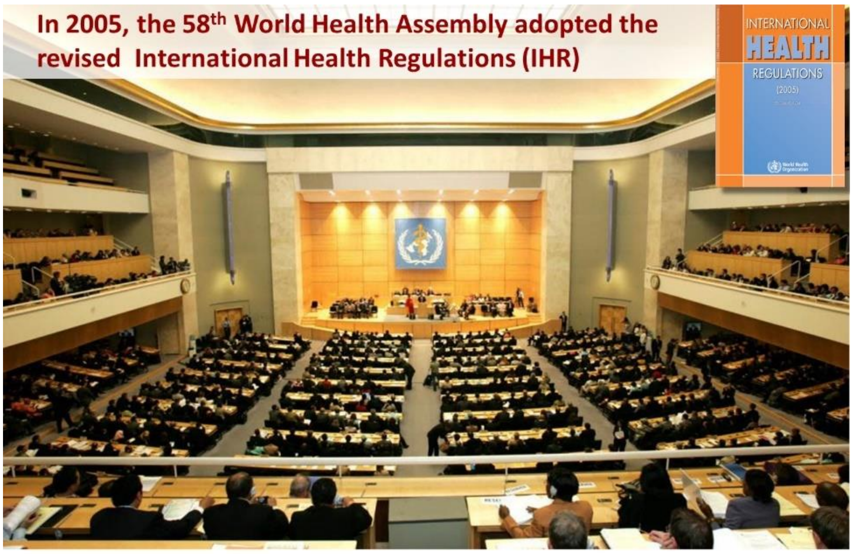

A legal commitment of 196 States Parties that have agreed to play by the same rules to secure international health.

Purpose of the IHR (2005)

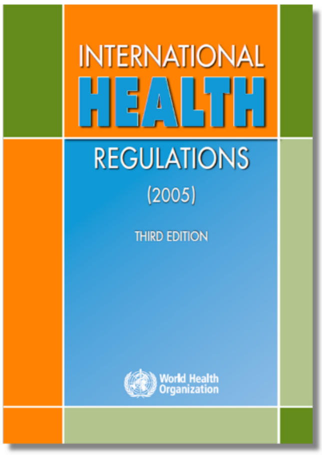

*" to prevent, protect against, control and provide a **public health response to the international spread of disease** in ways that are commensurate with and restricted to public health risks, and which **avoid unnecessary interference with international traffic and trade**"*

IHR (2005), article 2

Purpose of the IHR (2005)

*"Each State Party **shall develop**, strengthen and maintain, as soon as possible (...), **the capacity to detect, assess, notify and report events** in accordance with these Regulations... and ... **the capacity to respond** promptly and effectively..."*

IHR (2005), articles 5 and 13

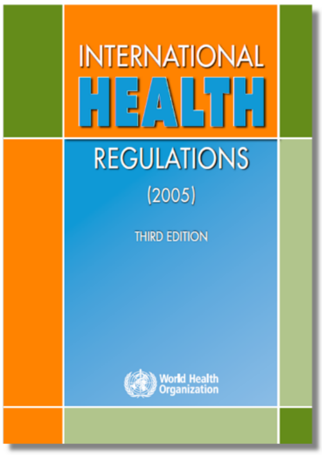

IHR Monitoring and Evaluation Framework (MEF)

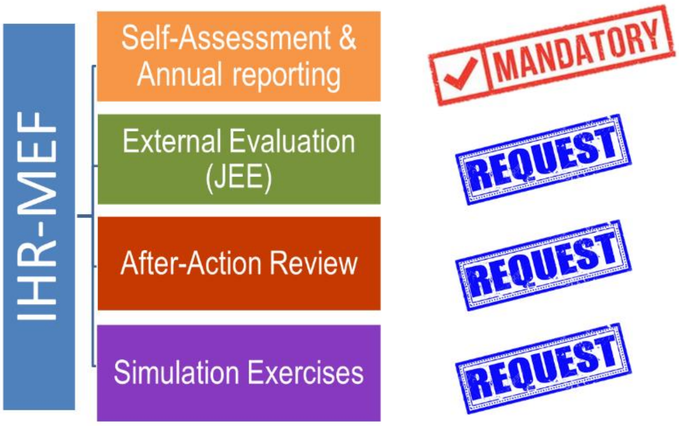

IHR Monitoring and Evaluation Framework (MEF)

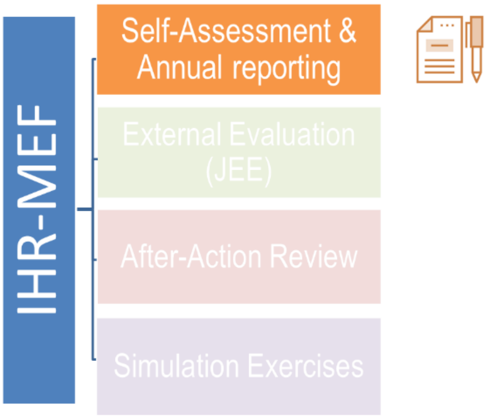

Country does a self evaluation and sends the results to WHO every year

IHR MEF – Self assessment and annual reporting

IHR – Indicators for assessment and monitoring progress

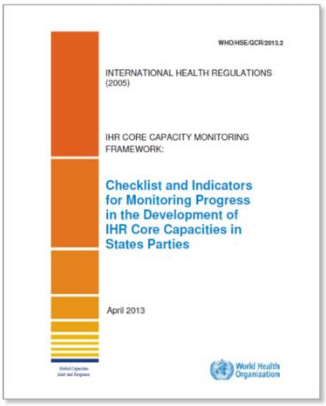

8 Core Capacities

1. National legislation, policy and financing
2. Coordination and NFP Communications
3. Surveillance
4. Preparedness
5. Response
6. Risk Communications
7. Human Resource Capacity
8. Laboratory

9. Points of Entry

10. Specific Hazards

10.1. Zoonotic

10.2. Food safety

10.3. Chemical emergencies

10.4. Radiological emergencies

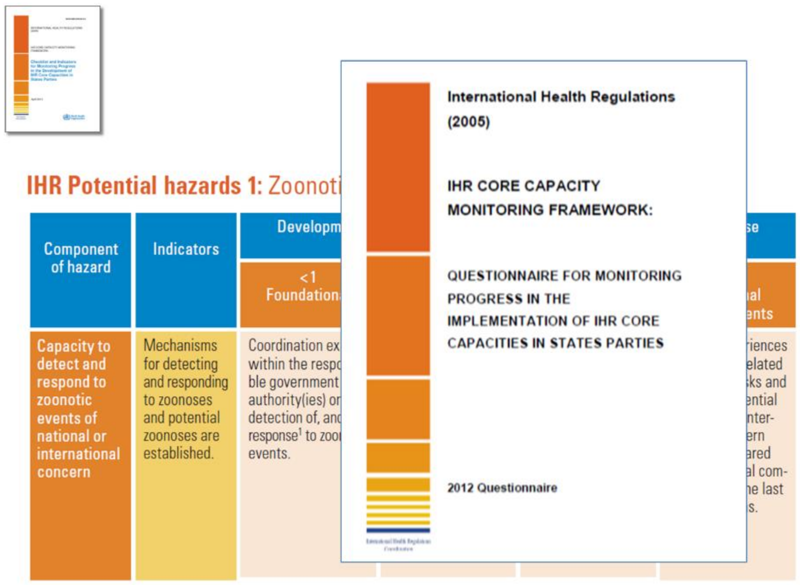

Core Capability	10	Zoonotic Events
Component	10.1	Capacity to detect and respond to zoonotic events of national or international concern
Indicator	10.1.1	*Mechanisms for detecting and responding to zoonoses and potential zoonoses are established and functional

NOTE: Before you begin, please review the general instructions for completing the questionnaire. Mark one appropriate value (Yes, No, or Not Known) for each of the questions below. A 'Not Known' value will be statistically equivalent to a 'No' value. If a question is not applicable for your country context please indicate this in the comment box below.

- 10.1.1.1 Does coordination exist within the responsible government authority (ies) for the detection of and response[90] to zoonotic events?

Yes
No
Not Known

10.1.1.2 Is there a national policy, strategy or plan in place for the surveillance and response to zoonotic events?

- 10.1.1.3 Have focal points responsible for animal health (including wildlife) been designated for coordination[91] with the MoH and/or IHR NFP [92]?

- 10.1.1.4 Have functional mechanisms[93] for intersectoral collaborations that include animal and human health surveillance units and laboratories been established?

- 10.1.1.5 Is a list of priority zoonotic diseases with case definitions available?

- 10.1.1.6 Is there systematic and timely collection and collation of zoonotic disease data?

- 10.1.1.7 Is there timely[94] and systematic information exchange between animal surveillance units, laboratories, human health surveillance units and other relevant sectors regarding potential zoonotic risks and urgent zoonotic events?

- 10.1.1.8 Does the country have access to laboratory capacity, nationally or internationally (through established procedures) to confirm priority zoonotic events?

IHR MEF – Self assessment and annual reporting

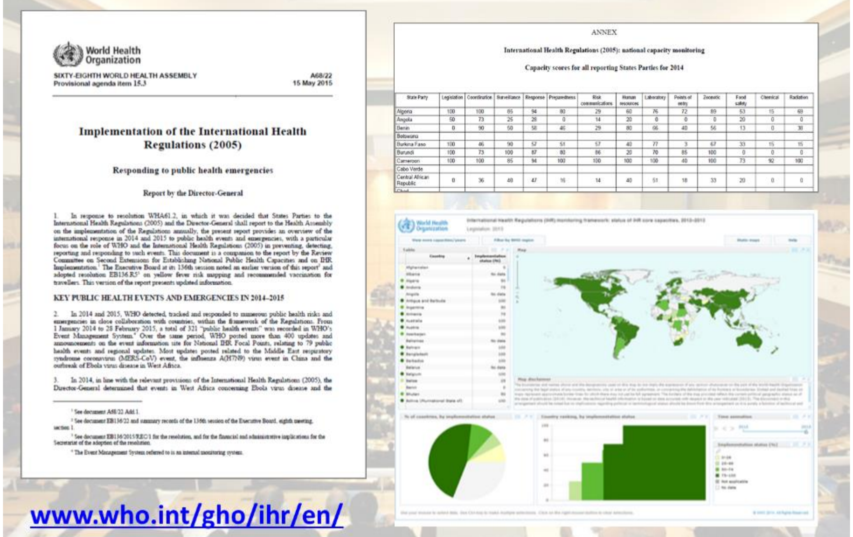

www.who.int/gho/ihr/en/

IHR MEF – Joint External Evaluation

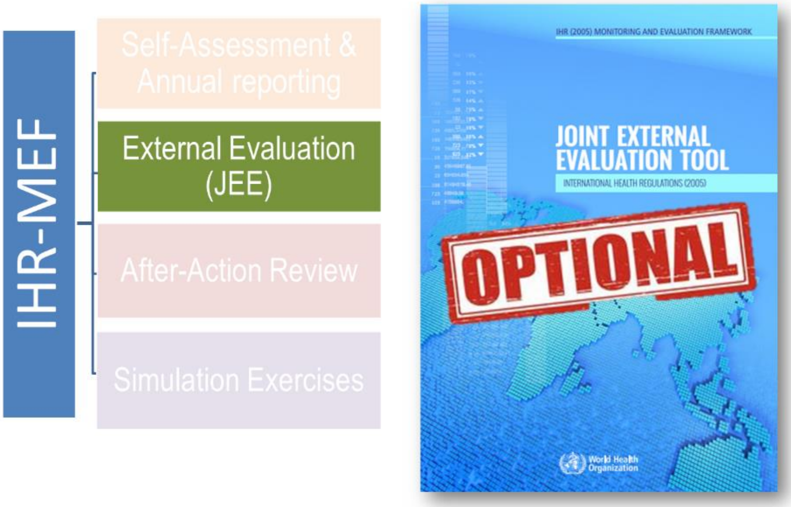

IHR MEF – Joint External Evaluation

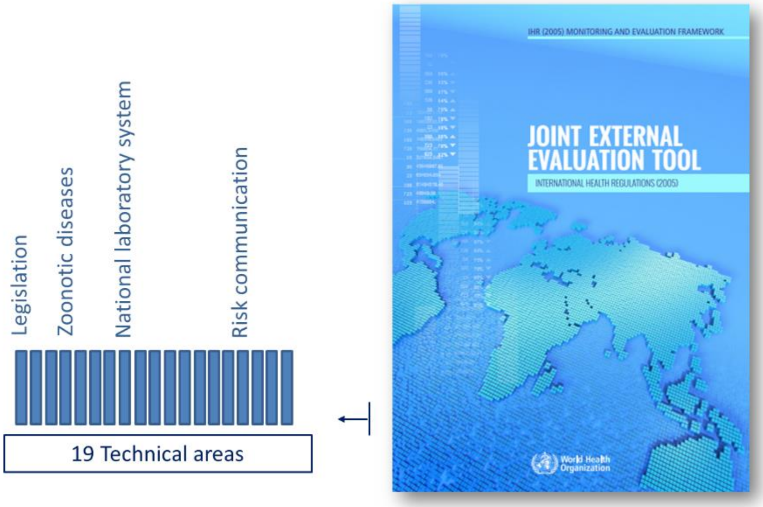

IHR MEF – Joint External Evaluation

48 indicators assessed	
1	No Capacity
2	Limited Capacity
3	Developed Capacity
4	Demonstrated Capacity
5	Sustainable Capacity

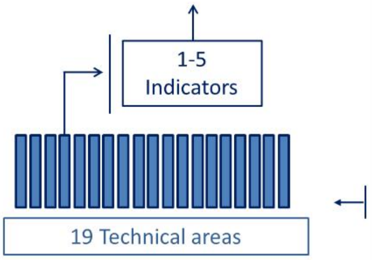

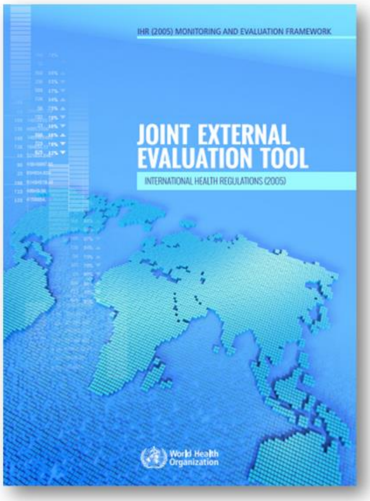

IHR MEF – Joint External Evaluation

Process

1. Country makes a request to WHO for a JEE
2. Country does a first self evaluation using the JEE tool
3. A JEE team of 10-12 international experts goes to the country for a one week mission
4. The JEE team reviews and discusses with national experts on all 19 technical areas
5. Priority actions are identified for each technical areas
6. All indicators are scored on a scale of 1-5 (consensus based)

http://www.who.int/ihr/procedures/mission-reports/en/

IHR MEF – After Action Review

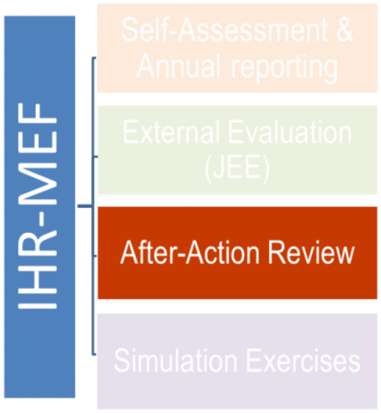

- After an outbreak
- Workshop in-country with all sectors involved in the response
- Objective is to analyse and build on the lessons learned from this event to improve levels of preparedness for future events

IHR MEF – Simulation Exercises

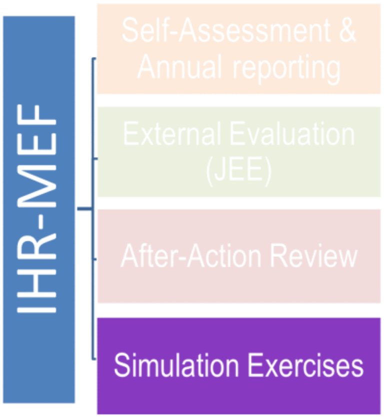

- Workshop in-country with all sectors involved in the response to outbreaks
- Fake outbreak scenario
- Testing of coordination mechanisms and contingency plans

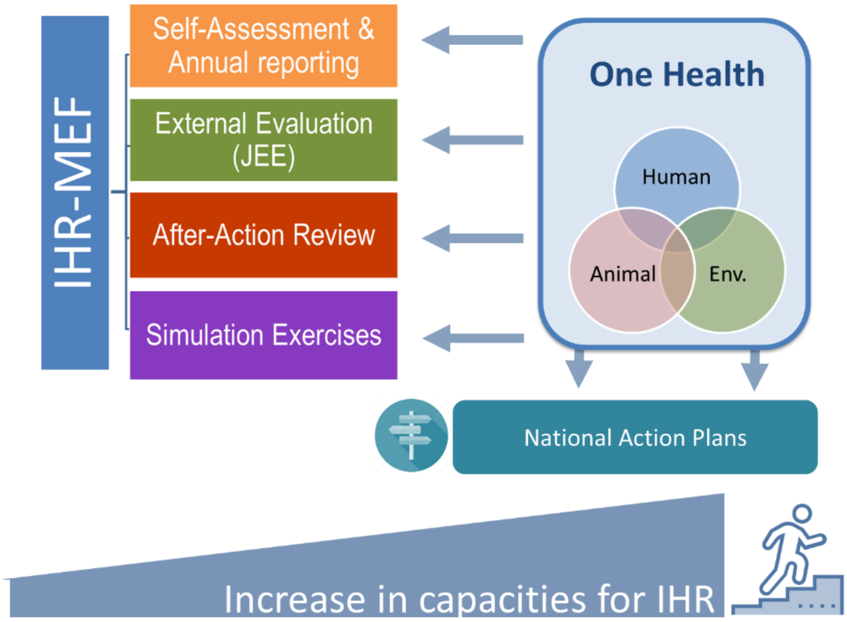

MOVIE 4: PVS PATHWAY

After a quick refresher about the roles and mandate of the OIE, this video presents the PVS Pathway. It explains the different steps of the pathway, their purpose and scope, how they are conducted and what outputs are produced.

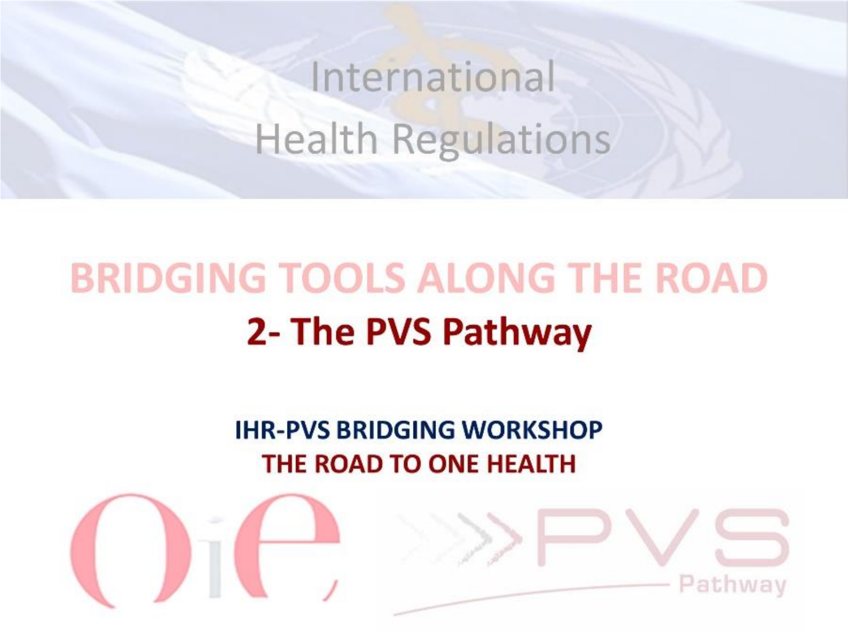

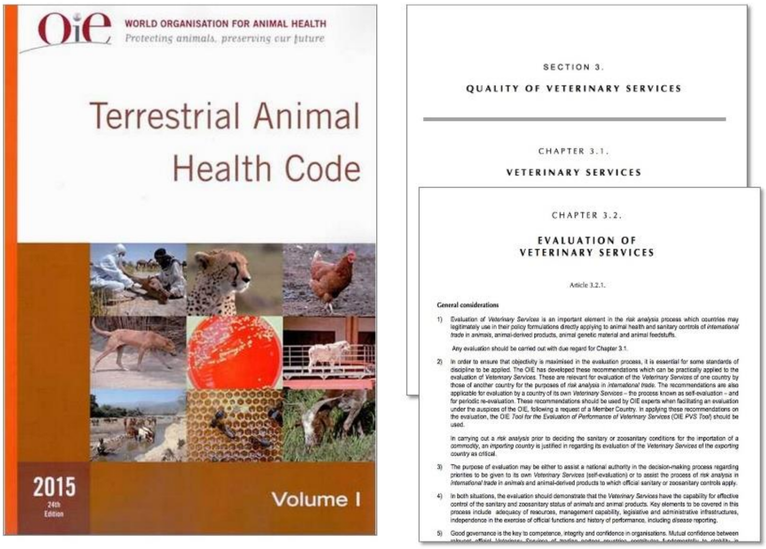

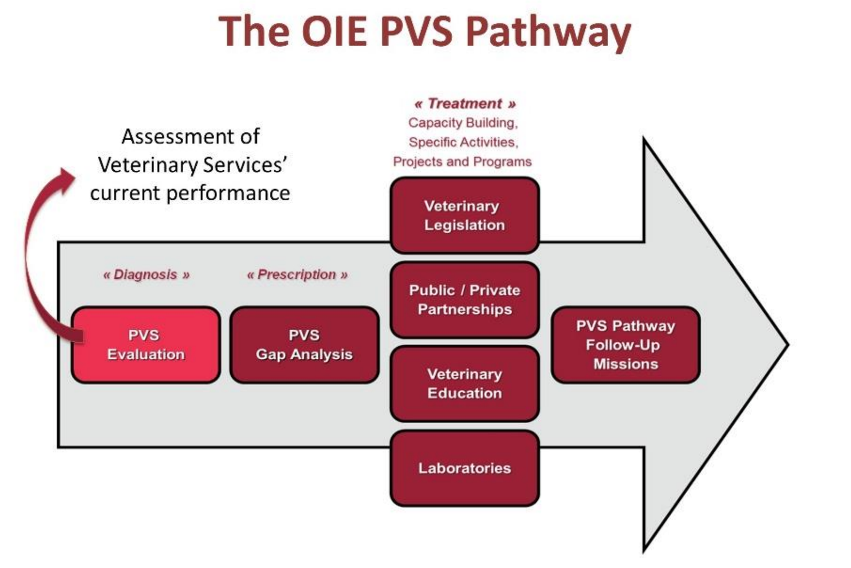
The OIE PVS Pathway
« Treatment »
Capacity Building,
Specific Activities,
Projects and Programs
Assessment of
Veterinary Services'
current performance
« Diagnosis »
« Prescription »
PVS
Evaluation
PVS
Gap Analysis
Veterinary
Legislation
Public / Private
Partnerships
Veterinary
Education
Laboratories
PVS Pathway
Follow-Up
Missions

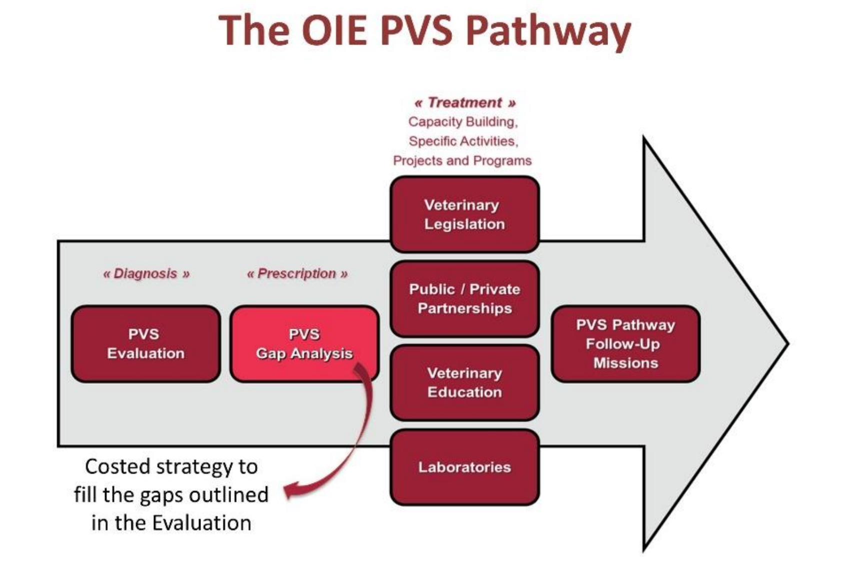
The OIE PVS Pathway
« Treatment »
Capacity Building,
Specific Activities,
Projects and Programs
« Diagnosis »
« Prescription »
PVS
Evaluation
PVS
Gap Analysis
Veterinary
Legislation
Public / Private
Partnerships
Veterinary
Education
Laboratories
PVS Pathway
Follow-Up
Missions
Costed strategy to
fill the gaps outlined
in the Evaluation

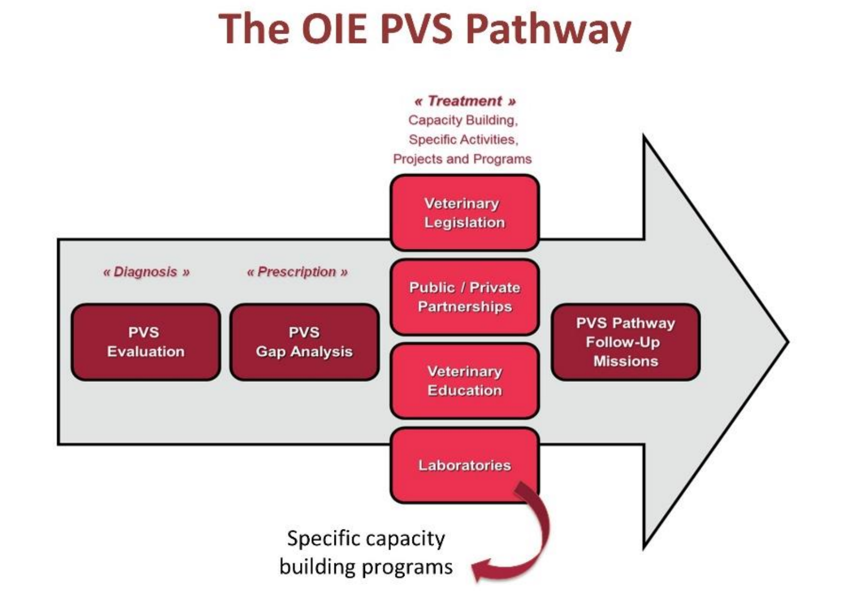
The OIE PVS Pathway
« Treatment »
Capacity Building,
Specific Activities,
Projects and Programs
« Diagnosis »
« Prescription »
PVS
Evaluation
PVS
Gap Analysis
Veterinary
Legislation
Public / Private
Partnerships
Veterinary
Education
Laboratories
PVS Pathway
Follow-Up
Missions
Specific capacity
building programs

The OIE PVS Pathway

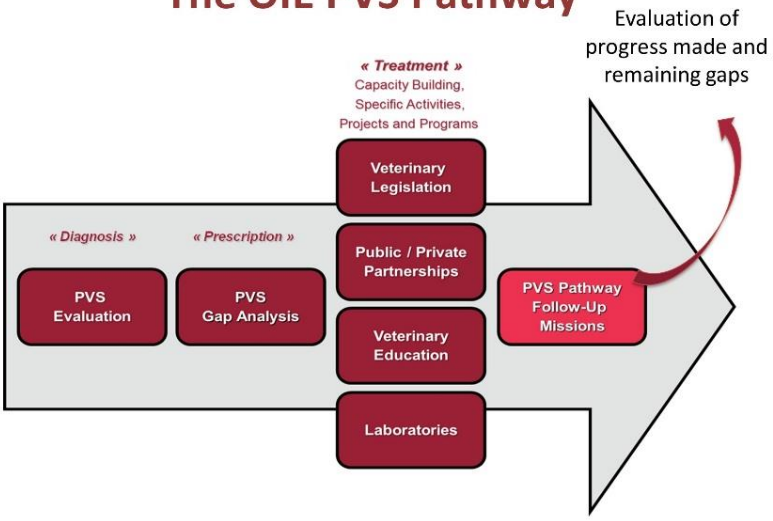

International Health Regulations

Step 1: PVS Evaluation

PVS Evaluation

- Performed jointly by national and OIE trained and certified external experts

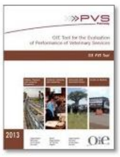

- Robust methodology based on the **systematic review** of **Critical Competencies** (CC) covering all the veterinary domain

CC on human, physical and financial resources

1. Professional and technical staffing
2. Competencies of veterinarians and vet. para-professionals
3. Continuing education
4. Technical independence
5. Stability and sustainability
6. Physical resources
7. Operational funding
8. Emergency funding
9. Capital investment
10. Management of resources and operations

- A total of **47 CCs** are discussed with national counterparts during a 2-3 weeks **mission**.

PVS Evaluation

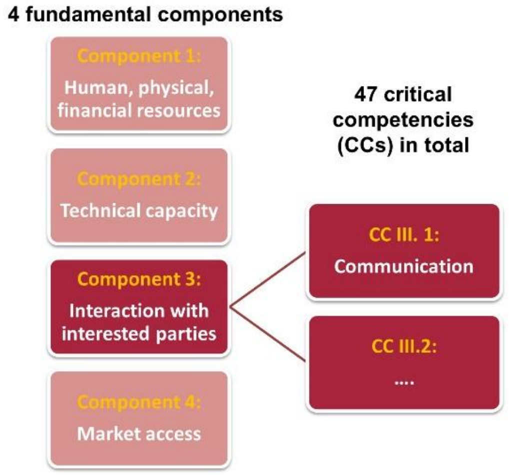

PVS Evaluation

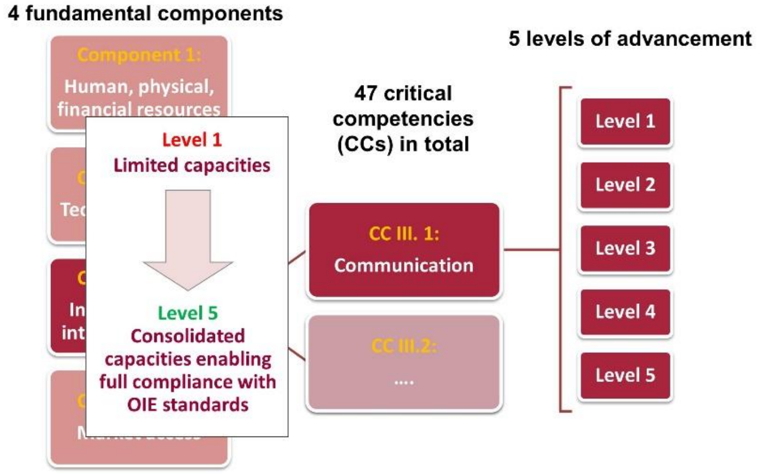

Example of a CC Card

III-1 - Communication	Levels of advancement
The capability of the VS to keep interested parties informed, in a transparent, effective and timely manner, of VS activities and programs, and of developments in animal health and food safety.	1. The VS have **no mechanism** in place to inform stakeholders of VS activities and programs.
	2. The VS have **informal** communication mechanisms.
	3. The VS maintain an official contact point for communications but it is **not always up-to-date in providing information**.
	4. The VS contact point for communications provides **up-to-date information**, **accessible** via the Internet and other appropriate channels, on activities and programs.
	5. The VS have a well-developed **communication plan, and actively and regularly** circulate information to stakeholders.

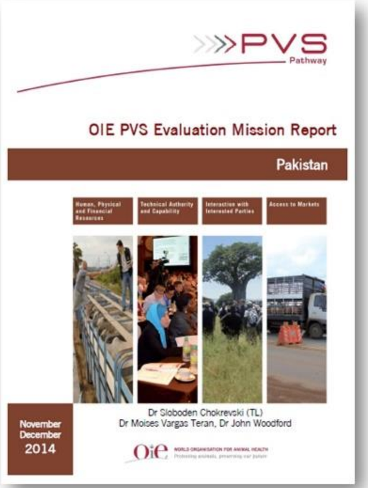

PVS Evaluation: final report

- The level of confidentiality of these reports is the decision of the countries
- Countries can waive this confidentiality and make reports accessible to other organizations or even to the general public
- Publicly available evaluation reports can be found on the OIE website

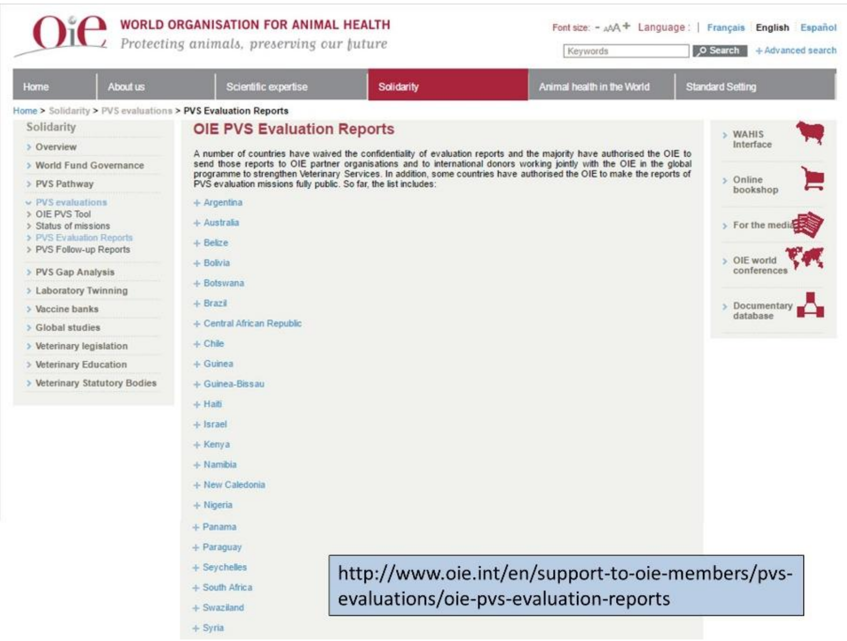

http://www.oie.int/en/support-to-oie-members/pvs-evaluations/oie-pvs-evaluation-reports

International Health Regulations

Step 2: PVS Gap Analysis

Example of a CC Card

III-1 - Communication	Levels of advancement
The capability of the VS to keep interested parties informed, in a transparent, effective and timely manner, of VS activities and programs, and of developments in animal health and food safety.	1. The VS have **no mechanism** in place to inform stakeholders of VS activities and programs.
	2. The VS have **informal** communication mechanisms.
	3. The VS maintain an of[illegible] contact point for communications but it is [illegible]**lways up-to-date in providing information**.
	4. The VS contact point for communications provides **up-to-date information**, **accessible** via the Internet and other appropriate channels, on activities and programs.
	5. The VS have a well-developed **communication plan, and actively and regularly** circulate information to stakeholders.

Example of a CC Card

**3. Strategy (if relevant)**		
Create a full time position in the VS for communication		
**4. Tasks to implement (chronological)**		
Specific tasks		1. **Recruit competent university degree on communication, with relevant physical resources**<br>2. **Secure sufficient budget for communication** (estimated on the basis of posters, broadcasting, leaflets, etc.)
Tasks linked to cross-cutting competencies	III.2 Consultation	*IT support*
	IV.1, 2, 3. Legislation	
	I.3. Continuing Education	*Training on outbreak communication*
**5. Objectively verifiable indicators (OIE PVS or specific)**		
- Job description<br>- Communication materials		

22

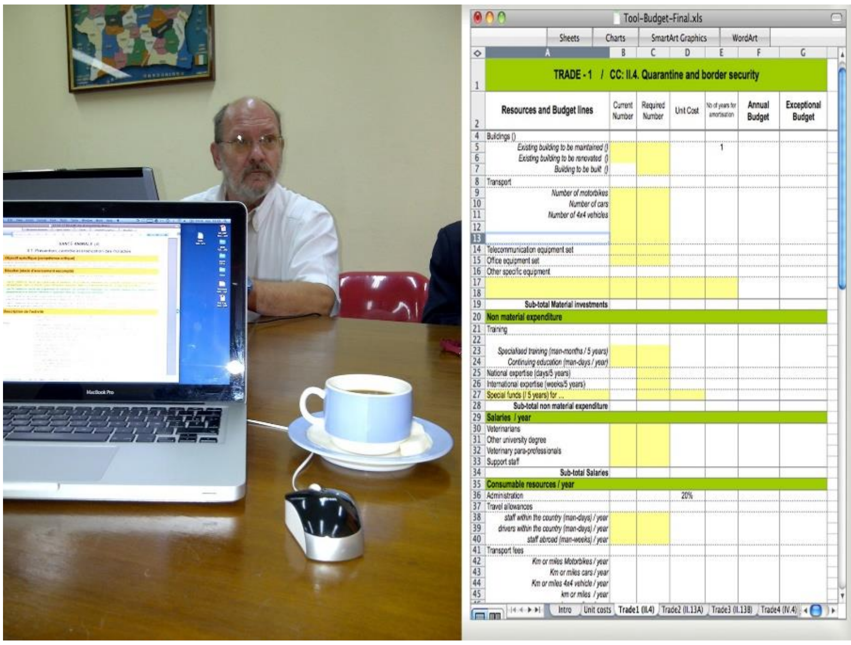

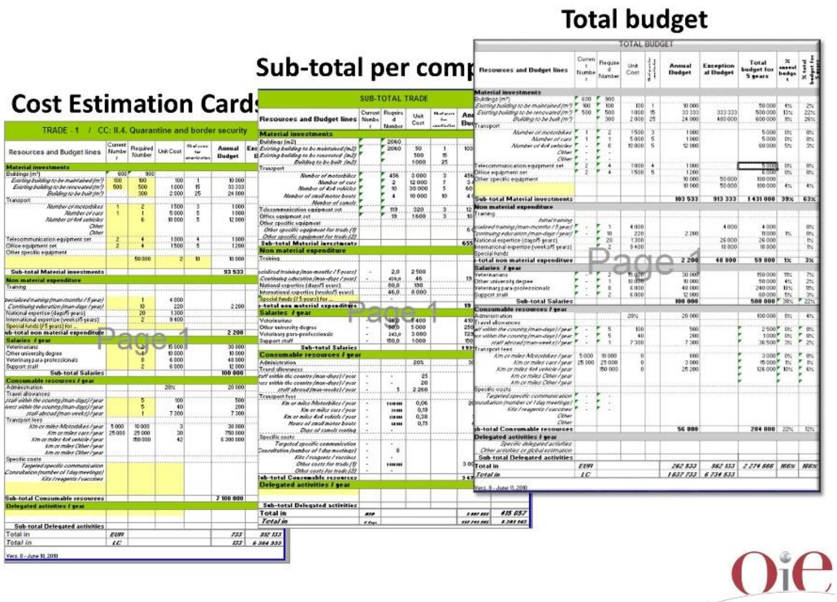

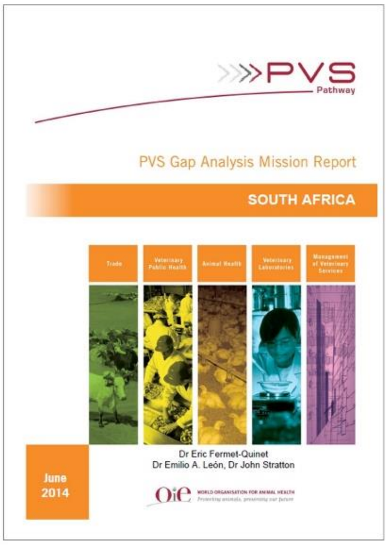

PVS Gap Analysis: final report

- Summary of priorities
- Indicative strategy and desired level of advancement for each CC
- Indicative costing

The level of confidentiality of these reports is the decision of the countries. Publicly available evaluation reports can be found on the OIE website

MOVIE 5: IHR-PVS BRIDGING

This brief video helps participants to understand how the OIE and WHO tools can be bridged. It shows how the Technical Areas of the IHR MEF can intersect or overlap with the Critical Competencies of the PVS Pathway. It presents the IHR-PVS matrix which will be used in the next exercise.

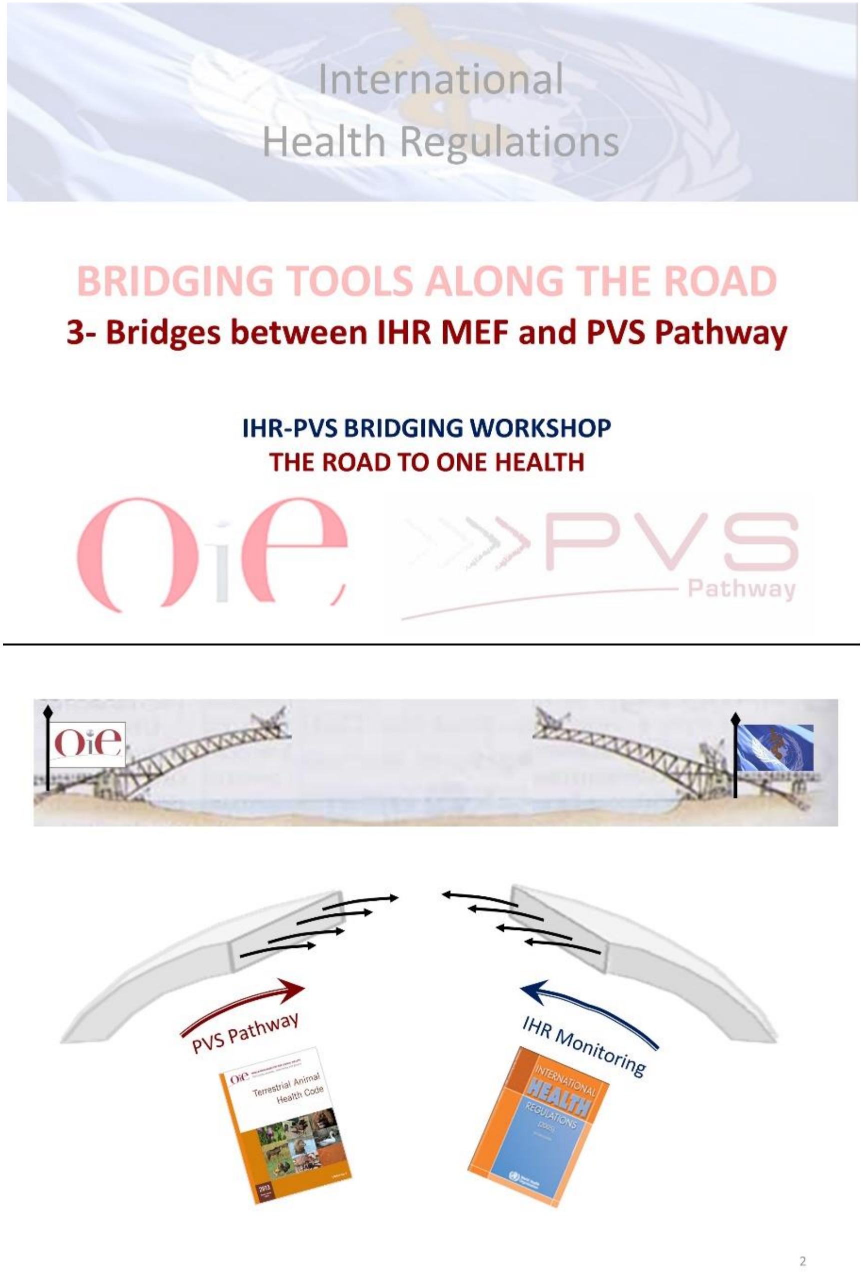

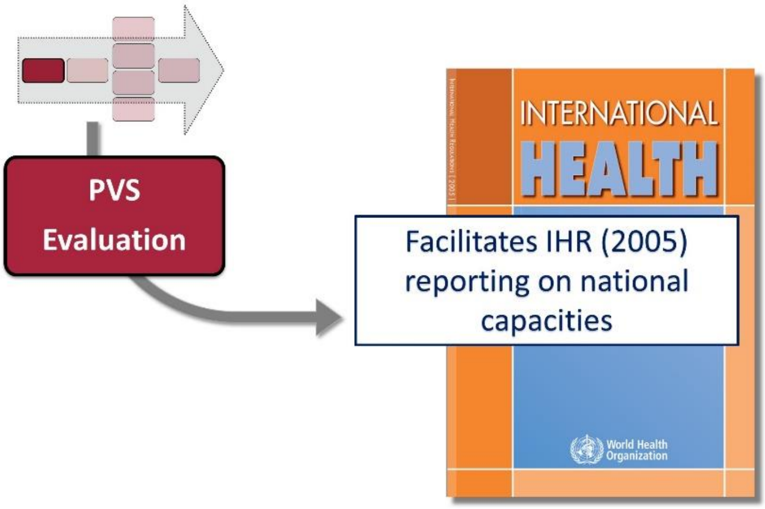

		PVS Tool				
		Critical Compentency 1	Critical Compentency 2	Critical Competency 3	…	Critical Competency 47
IHR Monitoring Framework	Indicator 1		X			
	Indicator 2	X		X		X
	Indicator 3		X			
	…					
	Indicator 28					X

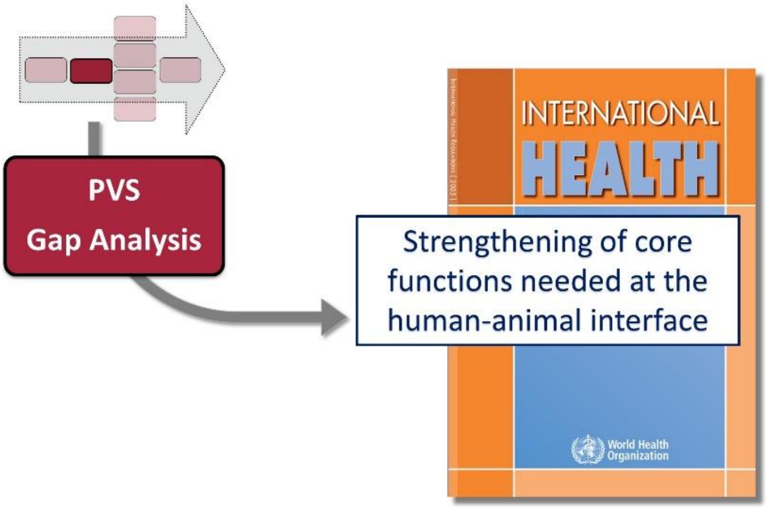

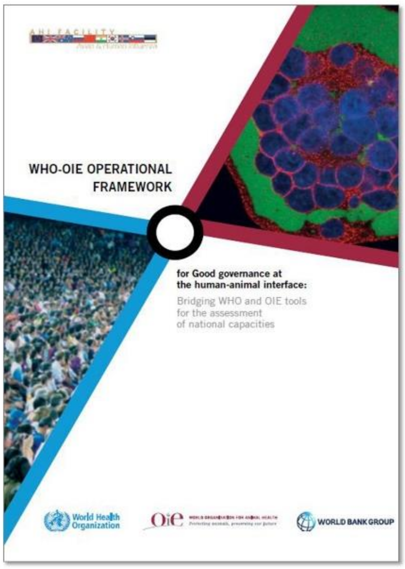

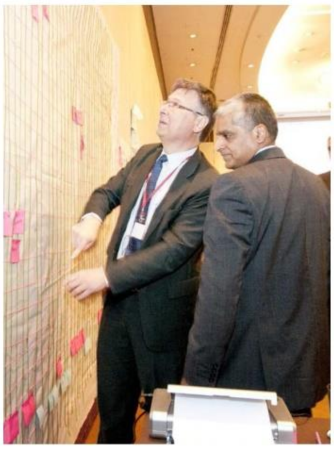

WHO and OIE experts working on the matrix of IHR Core Capacities and PVS Critical Competencies during a national workshop

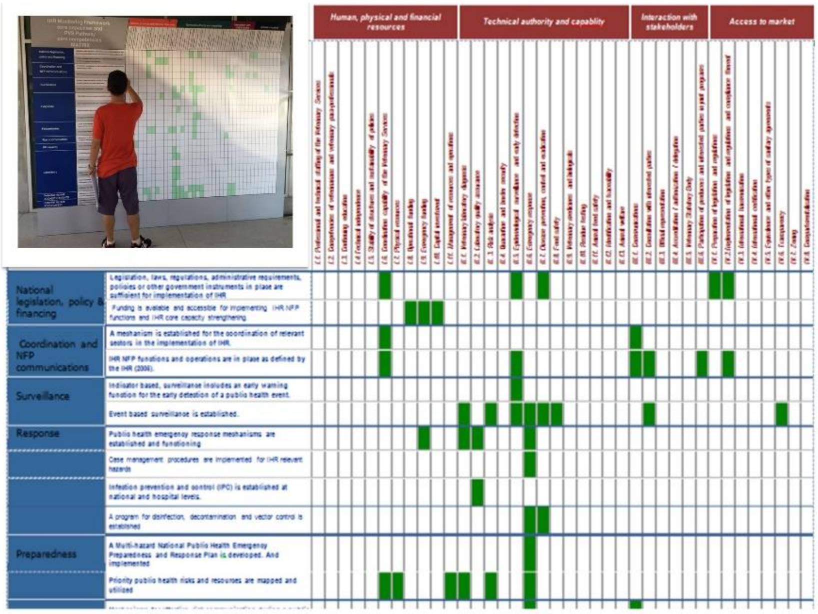

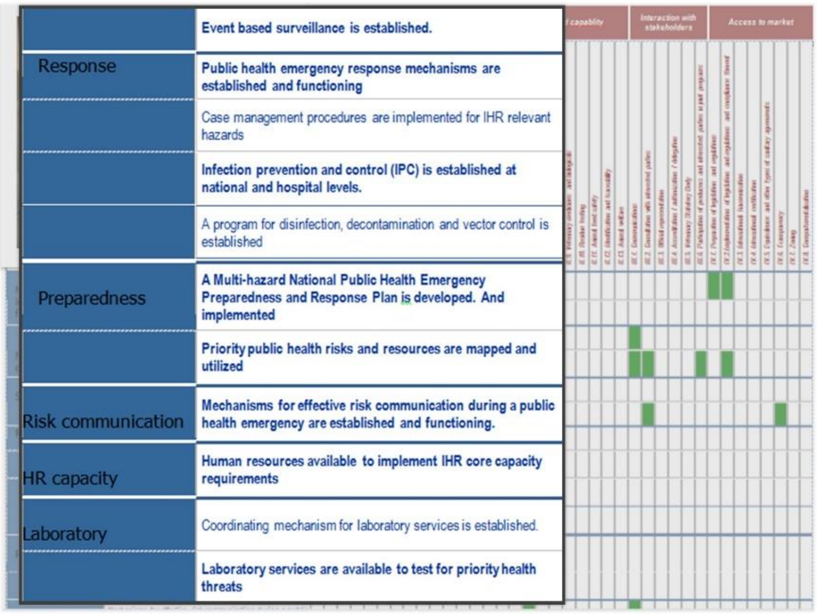

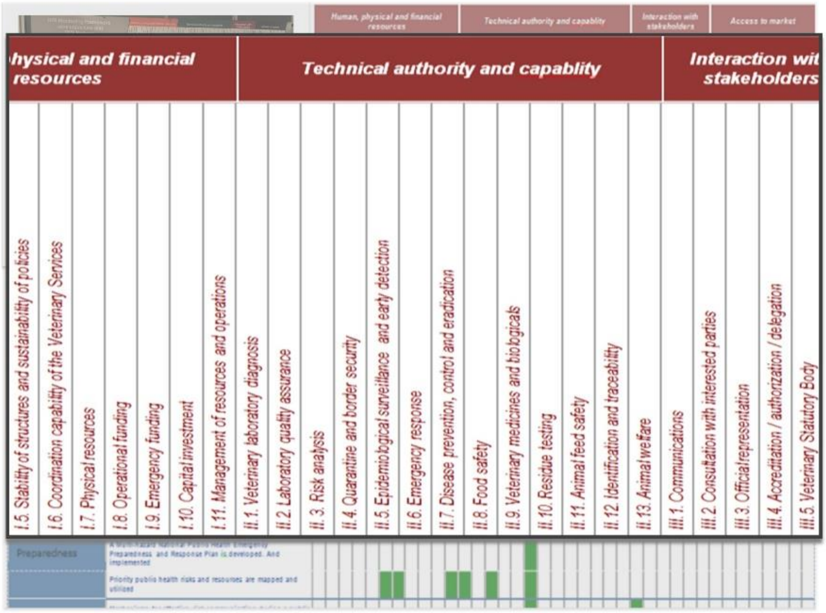
hysical and financial resources
Technical authority and capablity
Interaction wit stakeholders
I.5. Stability of structures and sustainability of policies
I.6. Coordination capability of the Veterinary Services
I.7. Physical resources
I.8. Operational funding
I.9. Emergency funding
I.10. Capital investment
I.11. Management of resources and operations
II.1. Veterinary laboratory diagnosis
II.2. Laboratory quality assurance
II.3. Risk analysis
II.4. Quarantine and border security
II.5. Epidemiological surveillance and early detection
II.6. Emergency response
II.7. Disease prevention, control and eradication
II.8. Food safety
II.9. Veterinary medicines and biologicals
II.10. Residue testing
II.11. Animal feed safety
II.12. Identification and traceability
II.13. Animal welfare
III.1. Communications
III.2. Consultation with interested parties
III.3. Official representation
III.4. Accreditation / authorization / delegation
III.5. Veterinary Statutory Body

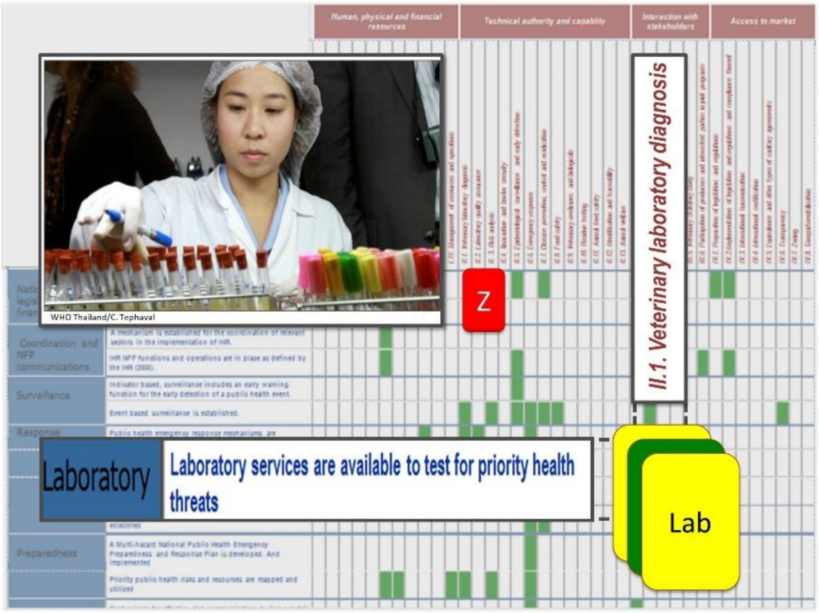
WHO Thailand/C. Tephaval
Z
II.1. Veterinary laboratory diagnosis
Laboratory
Laboratory services are available to test for priority health threats
Lab

IHR-PVS MATRIX

PVS PATHWAY – Human, physical & financial resources

JOINT EXTERNAL EVALUATION (JEE)		I.1. Professional and technical staffing of the Veterinary Services	I.2. Competencies of veterinarians & veterinary para-professionals	I.3. Continuing education	I.4 Technical independence	I.5. Stability of structures and sustainability of policies	I.6. Coordination capability of the Veterinary Services	I.7. Physical resources	I.8. Operational funding	I.9. Emergency funding	I.10. Capital investment	I.11. Management of resources and operations
Prevent	National Legislation, Policy & Financing					□			■	■	□	
Prevent	IHR Coordination, Communication & Advocacy						■					
Prevent	Antimicrobial Resistance (AMR)											
Prevent	Zoonotic Disease						■		□			
Prevent	Food Safety						■					
Prevent	Biosafety & Biosecurity											
Prevent	Immunization											
Detect	National Laboratory System											
Detect	Surveillance						□					
Detect	Reporting						□					
Detect	Workforce Development / Human Resources	■	■	■					□			□
Respond	Preparedness / Emergency Preparedness						□	□		■		□
Respond	Emergency Response Operations						□	□		■		
Respond	Linking Public Health & Security Authorities						■					
Respond	Medical Countermeasures & Personnel Deployment											
Respond	Risk Communication						□					
Other	Points of Entry (PoE)											
Other	Chemical Events											
Other	Radiation Emergencies											

PVS PATHWAY – Technical authority & capability

JOINT EXTERNAL EVALUATION (JEE)		II.1. Veterinary laboratory diagnosis	II.2. Laboratory quality assurance	II. 3. Risk analysis	II.4. Quarantine and border security	II.5. Epidemiological surveillance and early detection	II.6. Emergency response	II.7. Disease prevention, control and eradication	II.8. Food safety	II.9. Veterinary medicines and biologicals	II.10. Residue testing	II.11. Animal feed safety	II.12. Identification and traceability	II.13. Animal welfare
Prevent	National Legislation, Policy & Financing													
Prevent	IHR Coordination, Communication & Advocacy													
Prevent	Antimicrobial Resistance (AMR)	■	□							■	■			
Prevent	Zoonotic Disease	■	□	□		■	■	■						
Prevent	Food Safety	■	□	□		■			■		■		■	
Prevent	Biosafety & Biosecurity	■	■					□						
Prevent	Immunization													
Detect	National Laboratory System	■	■								□			
Detect	Surveillance			□		■								
Detect	Reporting					□	■							
Detect	Workforce Development / Human Resources													
Respond	Preparedness / Emergency Preparedness			□			■							
Respond	Emergency Response Operations					□	■	■						
Respond	Linking Public Health & Security Authorities													
Respond	Medical Countermeasures & Personnel Deployment													
Respond	Risk Communication			□										
Other	Points of Entry (PoE)				□									
Other	Chemical Events													
Other	Radiation Emergencies													

PVS PATHWAY – Interaction with stakeholders

JOINT EXTERNAL EVALUATION (JEE)		III.1. Communication	III.2. Consultation with interested parties	III.3. Official representation	III.4. Accreditation / authorisation / delegation	III.5. Veterinary Statutory Body	III.6. Participation of producers & interested parties in joint programs
Prevent	National Legislation, Policy & Financing						
Prevent	IHR Coordination, Communication & Advocacy	□	■	□			□
Prevent	Antimicrobial Resistance (AMR)						
Prevent	Zoonotic Disease						
Prevent	Food Safety						
Prevent	Biosafety & Biosecurity						
Prevent	Immunization						
Detect	National Laboratory System						
Detect	Surveillance						□
Detect	Reporting						
Detect	Workforce Development / Human Resources						
Respond	Preparedness / Emergency Preparedness						
Respond	Emergency Response Operations						
Respond	Linking Public Health & Security Authorities		■				
Respond	Medical Countermeasures & Personnel Deployment						
Respond	Risk Communication	■					
Other	Points of Entry (PoE)						
Other	Chemical Events						
Other	Radiation Emergencies						

PVS PATHWAY – Access to market

JOINT EXTERNAL EVALUATION (JEE)		IV.1. Preparation of legislation and regulations	IV.2.Implementation of legislation and regulations and compliance	IV.3. International harmonisation	IV.4. International certification	IV.5. Equivalence and other types of sanitary agreements	IV.6. Transparency	IV.7. Zoning	IV.8. Compartmentalisation
Prevent	National Legislation, Policy & Financing	■	■	□					
Prevent	IHR Coordination, Communication & Advocacy								
Prevent	Antimicrobial Resistance (AMR)								
Prevent	Zoonotic Disease								
Prevent	Food Safety								
Prevent	Biosafety & Biosecurity								
Prevent	Immunization								
Detect	National Laboratory System								
Detect	Surveillance								
Detect	Reporting		□				■		
Detect	Workforce Development / Human Resources								
Respond	Preparedness / Emergency Preparedness								
Respond	Emergency Response Operations								
Respond	Linking Public Health & Security Authorities		■						
Respond	Medical Countermeasures & Personnel Deployment								
Respond	Risk Communication								
Other	Points of Entry (PoE)				□				
Other	Chemical Events								
Other	Radiation Emergencies								

Comparison table JEE vs PVS Evaluation

	JEE (WHO)	PVS Evaluation (OIE)
Full name	Joint External Evaluation	Performance of Veterinary Services Evaluation
Framework	The JEE is one of the 4 components of the IHR Monitoring and Evaluation Framework	The PVS Evaluation is the first step of the PVS Pathway
Objective	Assesses the capacities of the country to respond to public health threats and their compliance with IHR	Assesses the capacities of Veterinary Services and their compliance with OIE standards
Obligation	Voluntary process (request made by country)	
Assessors	External experts + National counterparts	
Format	Self-assessment + 5-day mission (1-day site visits) of external experts	2-3-week mission (many site visits) of external experts
Indicators	49 indicators (in 19 technical areas)	47 indicators named Critical Competencies (in 4 components)
Scoring	Each indicator is scored on a 1-5 scale	
Gaps identified	✓ For each indicator	
Recommendations	✓ For each indicator	
Confidentiality	Report is made public	To be decided by the country
Follow-up	Outcomes to feed into National Action Plan for Health Security	Outcomes to feed into PVS Gap Analysis

PVS Evaluation – Extract from a report (South Africa)

This is the page for Critical Competency **II.3** on **Risk analysis**

There is a total of **47** Critical Competencies in the PVS evaluation

The score given was **3**

OIE PVS Evaluation – October 2012

II-3 Risk analysis	Levels of advancement
The authority and capability of the VS to base its risk management measures on risk assessment.	1. Risk management measures are not usually supported by risk assessment.
	2. The VS compile and maintain data but do not have the capability to carry out risk analysis. Some risk management measures are based on risk assessment.
	**3. The VS compile and maintain data and have the capability to carry out risk analysis. The majority of risk management measures are based on risk assessment.**
	4. The VS conduct risk analysis in compliance with relevant OIE standards, and base their risk management measures on the outcomes of risk assessment.
	5. The VS are consistent in basing sanitary measures on risk assessment, and in communicating their procedures and outcomes internationally, meeting all their OIE obligations (including WTO SPS Agreement obligations where applicable).

List of documents in appendix

***Evidence** (Appendix 6):* H6-10, P185-186,

Summary of the findings for this Critical Competency

Findings:

There is no Risk Analysis unit and specifically dedicated staff although epidemiology staff at national and sometimes at provincial levels are conducting some risk assessments.

Two veterinarians at national level have received short course training on risk analysis but have not followed up with further development in this area. For instance, no further training has been done at the provincial level or worked with resources at the university

Risk analyses were completed for the importation of pork from non-PRRS free countries and the importation of sable antelope from Zambia.

Risk analysis on AI management is currently implemented by an independent foreign consultancy on request of the Ostrich Business Chamber, which declared that the VS have been unable to provide such independent risk analysis for 7 years.

Risk analysis is lacking for most animal health programs, leading to the maintenance of unrealistic targets and strategies which are then not implemented (e.g. TB, brucellosis, anthrax, etc.).

The categorisation of different production systems is not based on multifactorial analysis, but only on historical socio-economic factor which only differentiate between so-called commercial, emerging, communal and the subsistence sectors. Such classification limits the ability to undertake risk analysis using well defined animal production systems to develop programme plans and survey designs.

Strengths identified

Strengths:

- Clear understanding of risk analysis concept by some staff.
- Risk analysis is implemented for imports.

Weaknesses identified

Weaknesses:

- No specific/designated staff or unit and defined methodology for risk analysis.
- AH programs/activities are not designed or based on risk analysis.
- Insufficient understanding of the different production systems when developing risk based animal health programs.

Recommendations made by the experts for this Critical Competency

Recommendations:

- Develop a systematic approach to risk analysis with dedicated staff and unit at national level and expand training/skills to provincial level.
- Establish a comprehensive approach on typology (characterisation) of production systems based on a multifactorial analyse including species, breeds, numbers, feeding, land management, in-take and off-take, reproductions, inputs, self-consumption, marketing and sales, social background, workforce, education, etc…

JEE – Extract from a report (Vietnam)

Biosafety and biosecurity

This is the **6th** Technical Area (TA) of the JEE. There are **19** TAs in total.

Introduction

Research with infectious agents is critical for the development and availability of public health and medical tools that are needed to detect, diagnose, recognize, and respond to outbreaks of infectious disease of both natural and deliberate origin.

Target

A whole-of-government national biosafety and biosecurity system is in place, ensuring that especially dangerous pathogens are identified, held, secured and monitored in a minimal number of facilities according to best practices; biological risk management training and educational outreach are conducted to promote a shared culture of responsibility, reduce dual use risks, mitigate biological proliferation and deliberate use threats, and ensure safe transfer of biological agents; and country-specific biosafety and biosecurity legislation, laboratory licensing, and pathogen control measures are in place as appropriate.

Introduction to the TA and its target

Viet Nam level of capabilities

The assessment of the country's capacities for this technical area starts here

Viet Nam has made important progress in biosafety, as part of a broader plan for strengthening national capacity in public health laboratories. Progress has been made in developing a biosafety legislative framework and biosafety training capacity in the regional institutes that would enable delivery of training to staff from provincial and district laboratories. Biosafety would be further strengthened by reviewing biosafety legislation and regulations against the international standards and investment in maintenance and certification of key biosafety equipment such as biosafety cabinets in all biosafety level 2 (BSL-2) laboratories. For this to happen, there is a need to build capacity in provincial health departments and preventive medicine laboratories to inspect and certify laboratories in the provinces.

Summary of the findings

Recommendations for priority actions

- Strengthen capacity of provincial health departments to certify and inspect diagnostic laboratories (BSL-1/2).
- Commit resources to maintain key biosafety infrastructure, such as biosafety cabinets, in a sustainable manner.
- Implement targeted biosafety and biorisk management training throughout the country in a coordinated manner, to develop a large network of trainers and trained laboratory workers who can regularly access expertise, tools and manuals to support biosafety practices.

3-5 key recommendations for this TA

Indicators and scores

P.6.1 Whole-of-government biosafety and biosecurity system is in place for human, animal and agriculture facilities – Score 3

This is the first of the two indicators for this TA. It was given a score of **3**. There are **48** indicators in total for the 19 TAs.

Strengths/best practices

- The five-year plan for strengthening laboratory capacity in Viet Nam is a framework that recognizes the critical functions of the public health laboratory system and sets out clear objectives that contribute to the overall goal of strengthening laboratory capacity.
- Viet Nam is cognizant of the importance of developing biosecurity regulations, with awareness of requirements to establish systems and procedures to ensure containment of dangerous pathogens.

Strengths regarding the first indicator

Areas that need strengthening/challenges

- Build capacity and resources to service and certify biosafety cabinets to a national standard. This could be linked to a broader effort to develop capacity at provincial health departments to oversee relevant laboratories, including development of materials and training to strengthen assessment of BSL-1/2 laboratories.
- Promote regular internal audits and strengthen the assessment mechanism for certification of BSL-1/2 laboratories.

Gaps and recommendations identified for the first indicator

P.6.2 Biosafety and biosecurity training and practices – Score 3

This is the second indicator for this TA. It was given a score of **3**.

Strengths/best practices

- Hubs of expertise in biosafety identified through four regional institutes that are responsible for conducting training of laboratory workers.
- Laboratory personnel, facilities, equipment and performance in national, regional and provincial laboratories were recently evaluated to enable targeted actions as part of a broader plan for strengthening national capacity in public health laboratories.

Strengths regarding the second indicator

Areas that need strengthening/challenges

- Mechanisms to monitor and document the effectiveness of training on biosafety and biosecurity for laboratory workers are required.
- Targeted biosafety and biorisk management training in a coordinated manner is needed throughout the country to improve biosafety practices by developing a network of trainers and trained laboratory workers who can regularly access expertise, tools and manuals.

Gaps and recommendations identified for the second indicator

EXERCISE 2: MAPPING OF GAPS ON THE IHR-PVS MATRIX

The same groups as for the first exercise are kept.

Process

1. Gather the 15 technical area cards that you have selected in the first exercise;
2. Give the cards numbered **2**, **3**, **5**, **8**, **9**, **13**, **14**, and **15** to the workshop facilitator;
3. Identify on your A1-size matrix poster where the seven remaining cards (**1**, **4**, **6**, **7**, **10**, **11**, **12**) fit-in by matching them to their corresponding indicators from the PVS (columns) and IHR (rows);
4. Position the seven cards of your group on the large matrix, using the blue-tack.

PLENARY: DISCUSSION

A plenary analysis of the outcome is conducted in front of the matrix. Gap clusters are identified and discussed.

**Notes**

Expected outcomes of Session 3:

- Understanding that tools are available to explore operational capacities in each of the sectors.
- Understanding of the contribution of the veterinary sector to the IHR.
- Understanding of the bridges between the IHR MEF and the PVS Pathway. Reviewing together the results of capacities assessment may help in identifying possible synergies and optimize collaboration.
- Understanding that most gaps identified are not disease-specific but systemic.
- Identification of the technical areas to focus on during the next sessions.

SESSION 4: EXTRACTION OF ASSESSMENT RESULTS

Objective: Explore the improvement plans already proposed in the respective assessments (IHR annual reporting, JEE, PVS Evaluation, etc.), extract relevant sections and identify what can be synergized and improved jointly.

EXERCISE 3: EXTRACTION OF ASSESSMENT RESULTS

Groups are now organized by technical area. Find a group for which you feel your expertise is relevant but ensure that participants from both sectors are equally represented in the technical groups.

Process

1. Each group identifies a chairman, a rapporteur and a time-keeper;
2. Using the two **indicator tables**, identify the sections from the PVS Evaluation and the JEE which are relevant to your technical area;
3. Extract the main gaps (up to 12) reported in the assessment documents and write them on the **Gap cards;**
4. Extract the main recommendations (up to 12) and report them on the **Recommendation cards;**
5. Position the **Gap** and **Recommendation** cards on the flip-chart with **blue-tack** and following this template:

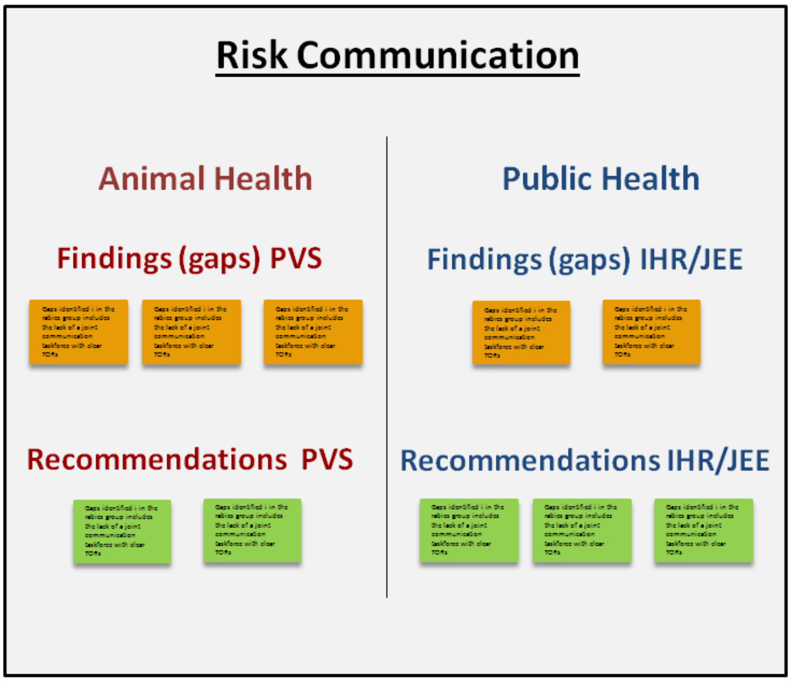

Answers to frequently asked questions or common mistakes

-Focus should be made on gaps/recommendations that are **somewhat relevant to One Health**. If a gap or recommendation is entirely specific to one sector it is not relevant.

-Groups should focus only on their technical area and **avoid overlap** with thematics addressed by other groups.

-Avoid the situation where veterinarians work on their report and public health service work on theirs. This is a good opportunity for each sector to know about the other sector and open their assessment reports. The group should go through all the tools together.

Important: There is no restitution of the working groups for this session because it is only a preliminary step for Session 5.

Material and documents

Flip-chart

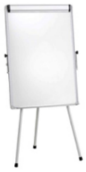

Gap cards

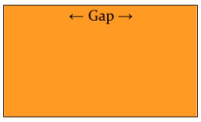

Recommendation cards

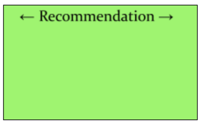

JEE report

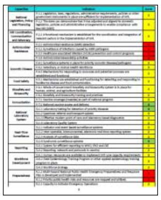

PVS Evaluation report

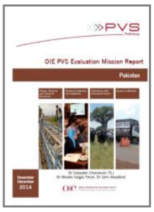

Indicator tables

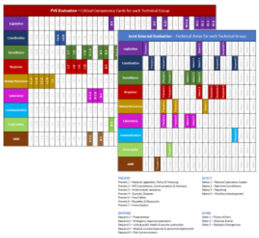

Fine point markers

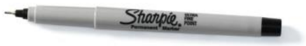

Blue-tack

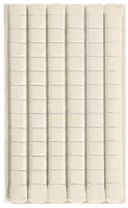

Expected outcomes of Session 4:

- Good understanding of the assessment reports for both sectors, their purpose and their structure.
- Main gaps relevant to each technical area have been extracted.
- Main recommendations from existing reports have been extracted.
- A common understanding of the effort needed starts to emerge.

SESSION 5: JOINT ROAD PLANNING

Objective: use the results obtained from the case studies and from the assessment reports to develop a realistic and achievable road-map to improve the collaboration between the sectors.

EXERCISE 4: IDENTIFICATION OF JOINT ACTIVITIES

The same groups (per technical area) as for the previous exercise are kept.

Process

1. Read fully these instructions before starting, including the good/bad examples on page 52.
2. Identify **realistic** and **achievable** JOINT ACTIVITIES (minimum 3, maximum 10) that would strengthen the inter-sectoral collaboration and improve performance for your thematic area.
3. Activities must fit the **SMART** criteria (**Specific**, **Measurable**, **Achievable**, **Relevant** and **Time-bound**). The activities need to be clearly understandable (What? How?) by just reading them, without requiring further information.
4. Write the activities on the flip-chart and discuss them with the facilitating team.
5. Fine-tune the activities according to the outcomes of the discussion.

Activities should not be defined only based on gaps identified in the assessment reports. Use all sources of information, including:

- The gaps identified in the case-study exercise (using the session 2 report-sheet)
- The gaps and recommendations found in the assessment reports (JEE, PVS, etc.)
- The discussions held during the workshop so far
- And most importantly, your ***personal experience***!

Answers to frequently asked questions or common mistakes

-Activities need to be clear and accurate. For example, "capacity building of communication staff" is not an activity, but "3-day training for 25 communication staff" is.

"Enhance", "Improve", "Harmonize", "Standardize" → **Not an activity**
"Create", "Conduct", "Produce", "Develop", "Prepare", "Draft" → **Activity**

-Activities should be clear enough so that someone who is not from your group can understand **precisely** what you will do and how you will do it, without the need for any further explanation.

-Use existing resources and material nationally and internationally: avoid developing big things that already exist elsewhere (ex: assessment tools, training curricula, etc)

-The back of the technical cards relevant to each group could contain some example of activities to use.

Important:

- It is essential to understand that you are **not** aiming at improving each sector, but that you are aiming to improve the **collaboration** between the two.
- Activities should be achievable: it is better to plan for little steps and to do them, than to plan for big leaps and to stand still!
- Make sure the activities are **SMART** (**Specific**, **Measurable**, **Achievable**, **Relevant** and **Time-bound**).

Some bad examples

Bad example	Reason
Conduct a training for staff	**Not specific.** Training for what? For who? How many trainings? Which level (national? Regional?)? For How many trainees?
Develop a response plan	**Not specific.** A plan for what exactly? Generic multi-hazard or disease specific? Which diseases? Which sectors?
Conduct a training of trainers at national level and run cascade trainings at the district level on risk communication	**Not realistic.** If the country has 600 districts, this is most likely impossible.<br>**Not relevant**. Does everyone really need training on risk communication?
Build capacity for joint response at field level	**Not specific.** How will you build capacity?<br>**Not measurable.** How can you measure implementation of this activity?

Some good examples of SMART activities

Set-up and institutionalize three joint technical area working groups (TAWG) at the national level for (1) surveillance activities, (2) risk communication, and (3) outbreak investigation and response
Set-up and institutionalize 9 joint rapid response teams (one at national level and one in each of the 8 regions)
Designate and institutionalize focal points for risk communication in each sector (1 at national level and 1 in each of the 8 regions)
Develop TORs and SOPs for information sharing between focal points in each sector
Develop an IT platform that links the data information systems of both sectors
Conduct a training needs analysis for outbreak investigation and response
Conduct a training of trainer at national level followed by a training in each region (8 total) on joint outbreak investigation for joint rapid response teams
Develop a joint multi-hazard response plan (with specific annexes for priority zoonotic diseases) involving both sectors
Conduct a joint-simulation exercise on a zoonotic disease every year to test contingency plans and procedures in place
Organize routine meetings of the joint technical area working groups every 6 weeks
Organize a joint risk assessment meeting every two months at the national level for priority zoonotic and food-borne diseases
Organize a consultative meeting with epidemiology and laboratory units from both sectors to harmonize processes and optimize shared logistics

Material and documents

Flip-chart

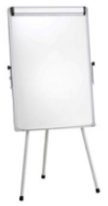

Session 2 results

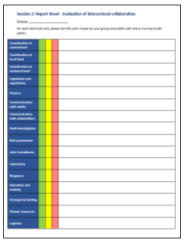

Session 4 results

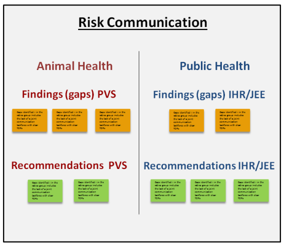

Fine point markers

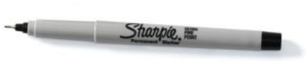

Technical card(s) relevant to your technical group

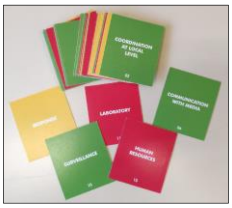

Expected outcome of Session 5:

- Clear and achievable activities are identified to improve inter-sectoral collaboration between the two sectors for all technical areas selected.

SESSION 6: FINALIZATION OF THE JOINT ROAD-MAP

Objective: To have all participants contribute to all technical areas and to consolidate the joint-road map by making sure it is harmonized, concrete and achievable.

EXERCISE 5: FINE-TUNING OF THE JOINT ROAD-MAP

The same groups (per technical area) as for the previous exercise are kept.

Process

1. Discuss with the facilitators to group the activities together under 1-to-3 specific objectives. Write the objectives on the **Objective cards**.
2. For **each** activity, fill up an **Activity card** indicating a desired date of achievement, who is responsible and explaining the **detailed** process of implementation.
3. Position the cards on a flipchart using blue-tack and the template shown on the next page.
4. For each activity, evaluate, using the coloured stickers, the cost of implementation and the level of impact this would have in terms of improvement by following the following scale:

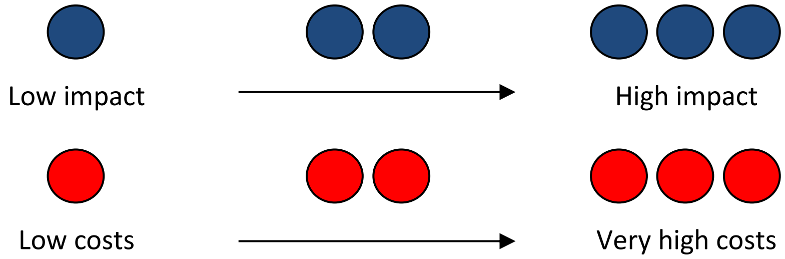

Answers to frequently asked questions or common mistakes

-Results will determine the future road-map, please use good hand-writing and avoid using acronyms.

-The cards must be sufficiently complete and clear, so that someone who is not in the workshop (for example your Minister of Finances) can understand precisely what you will undertake, why, and how you will implement it, by just reading the card. No further explanation should be required.

-Responsibility should be specific. “MoH and MoA” is not a satisfying answer for the box “Responsibility”.

-Use existing resources and material nationally and internationally: avoid developing big things that already exist elsewhere (ex: assessment tools, training curricula, etc)

Important:

- Activities should be achievable: it is better to plan for little steps and to do them, than to plan for big leaps and to stand still!
- Make sure the activities are **SMART** (**Specific**, **Measurable**, **Achievable**, **Relevant** and **Time-bound**).

Example of expected result (overall)

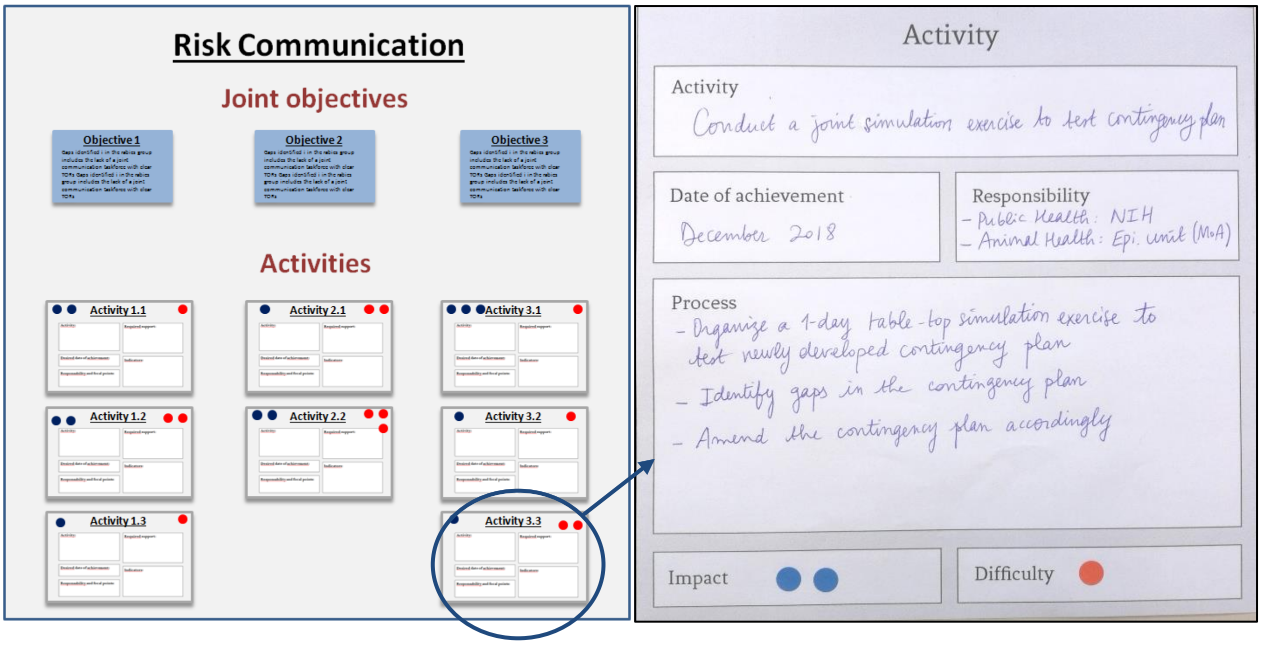

Example of expected result (detailed)

Objective 1: Set-up an operational framework for routine data-sharing of surveillance results between the animal health and human health sectors.

-Activity 1.1. Develop a Memorandum of Understanding (MoU) between the two Ministries for routine data-sharing of surveillance results.

Date of achievement: June 2019.

Process: -Organize a meeting with the two sectors at national level to draft the MoU;
-Circulate the drafted MoU for revision from both Ministries;
-Organize a validation workshop for official endorsement.

-Activity 1.2. Develop ToRs and SoPs for routine data sharing of surveillance results.

Date of achievement: August 2019.

Process: -Organize a technical meeting with the two sectors at national level to develop the ToRs and SoPs in line with the MoU;
-Validation of the ToRs and SoPs by both Ministries.

-Activity 1.3. Nominate a focal person in each sector at the national level and in each region who will be responsible for data-sharing.

Date of achievement: July 2019.

Process: -Each sector to designate a focal person at the national level and in each region, as per developed ToRs;
-Institutionalize the list of focal persons;
-Revise the list of focal persons on a yearly basis and amend if necessary.

Keep in mind:

-Objective = what do you want to reach?

-Activity = what exactly will you do?

-Process = how exactly will you do it?

Check-list to validate an Objective:

-Is my objective specific enough?

-Is my objective about improving collaboration and not just one sector's capacity?

-Can my Minister understand my objective from just reading the card?

Check-list to validate an Activity:

-Is my activity very specific?

-Is my activity measurable?

-Is my activity achievable?

-Is my activity relevant?

-Is my activity time-bound?

-Can my Minister understand everything about my activity from just reading the card?

-Does my activity answer all relevant questions such as: how? For who? Why? How many? Which level? Etc.

Material and documents

Flip-chart

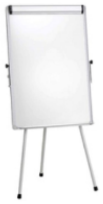

Objective cards

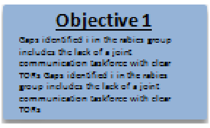

Activity cards

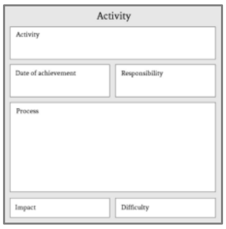

Fine point markers

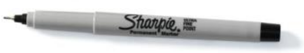

Blue-tack

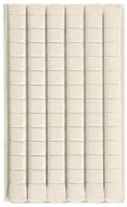

Red stickers (x30)

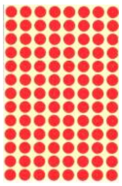

Blue stickers (x30)

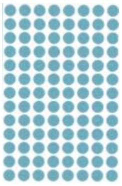

EXERCISE 6: WORLD CAFÉ

The World Café exercise enables participants to contribute to the action points of all technical areas. Each group will rotate through the other groups to make comments or ask for further information by leaving post-it notes. World café Instructions will be given by the facilitators.

Notes

EXERCISE 7: PRIORITIZATION VOTE

This exercise enables to evaluate the level of priority of the different activities defined.

Process

Each participant is given 5 stickers and must select the 5 objectives that they believe is of highest priority (voting for one objective means voting for all the activities it contains).

OR

If facilitators are using an online application voting system, you can access the vote by **either** scanning this QR code with a mobile phone **OR** by going to the following website: www.bit.ly/NBWVote using your computer or phone.

Expected outcomes of Session 6:

- Harmonized, concrete and achievable road-map
- Buy-in and ownership of all participants who feel that they contributed to all areas of the road-map.
- Prioritization of the activities.

SESSION 7: WAY FORWARD

Objective: the last session draws the way forward by identifying the next steps and by inscribing the developed road-map into other mandated plans such as the National Action Plan for Health Security. This is also where any need from the country can be addressed. This will depend greatly on the status of the country in terms of IHR-MEF and on the level of One Health capacity.

Notes

Expected outcomes of Session 7: Depends on the country needs and level of advancement in implementation of the IHR-MEF but options can include:

- Linkages with NAPHS.
- Identification of immediate and practical next steps.
- Identification of opportunities for other components of the IHR-MEF.

EVALUATION OF THE WORKSHOP

This questionnaire aims to collect your feedback and suggestions on the IHR-PVS National Bridging Workshop. The objective of WHO and OIE is to improve the quality of future events.

(Optional) Last name / first name: ……………………………………………….……

Your sector: Human health ☐ Animal Health☐ Environment ☐ Other ☐

Your level: National ☐ Regional ☐ Local/district ☐ Other ☐

Scale: 1 = Not satisfied at all 2 = Not really satisfied 3 = Satisfied 4 = Fully satisfied

	Satisfaction level	**Comment**<br>If rated 1 or 2, please justify
**Overall experience**	1☐ 2☐ 3☐ 4☐	
**Content**<br>(Quality, relevance, technical-level)	1☐ 2☐ 3☐ 4☐	
**Format**<br>(Method, material, activities)	1☐ 2☐ 3☐ 4☐	
**Facilitators**<br>(Communication skills, technical expertise)	1☐ 2☐ 3☐ 4☐	
**Organization**<br>(Logistics, venue, assistance)	1☐ 2☐ 3☐ 4☐	

Scale: 1 = No impact at all 2 = Weak impact 3 = Significant impact 4 = Highest impact

	**Impact**	**Comment**<br>If rated 1 or 2, please justify
How would you rate the impact of this event on:		
**Your technical knowledge on the subject matter**	1☐ 2☐ 3☐ 4☐	
**The work of your department/unit**	1☐ 2☐ 3☐ 4☐	
**The collaboration between AH and PH in your country**	1☐ 2☐ 3☐ 4☐	

Would you recommend this workshop to other countries?

Not at all ☐ Likely not ☐ Probably ☐ Absolutely ☐

Evaluation of the sessions

1 = Not satisfied at all 2 = Not really satisfied 3 = Satisfied 4 = Fully satisfied		
Please rate only the sessions you have attended	**Content, Format and Usefulness of the session**	**Comment** Please help us improve by justifying any 1 or 2 rating
**Session 1:** Setting the scene	1☐ 2☐ 3☐ 4☐	
**Session 2:** Case studies and evaluation of collaboration	1☐ 2☐ 3☐ 4☐	
**Session 3:** IHR & PVS tools, mapping of gaps on the IHR-PVS matrix and collective analysis	1☐ 2☐ 3☐ 4☐	
**Session 4:** Compilation of gaps & recommendations from existing reports	1☐ 2☐ 3☐ 4☐	
**Session 5:** Activities & objectives	1☐ 2☐ 3☐ 4☐	
**Session 6:** Fine-tuning of the road-map, World café, Prioritization vote	1☐ 2☐ 3☐ 4☐	
**Session 7:** Way forward	1☐ 2☐ 3☐ 4☐	

- **In your view, what were the main strengths of this workshop?**

- **In your view, what were the main weaknesses of this workshop?**

Thank you for taking the time to fill out this form. Please return it to one of the organisers.

Notes

Owner of this Handbook

Name:
Email:

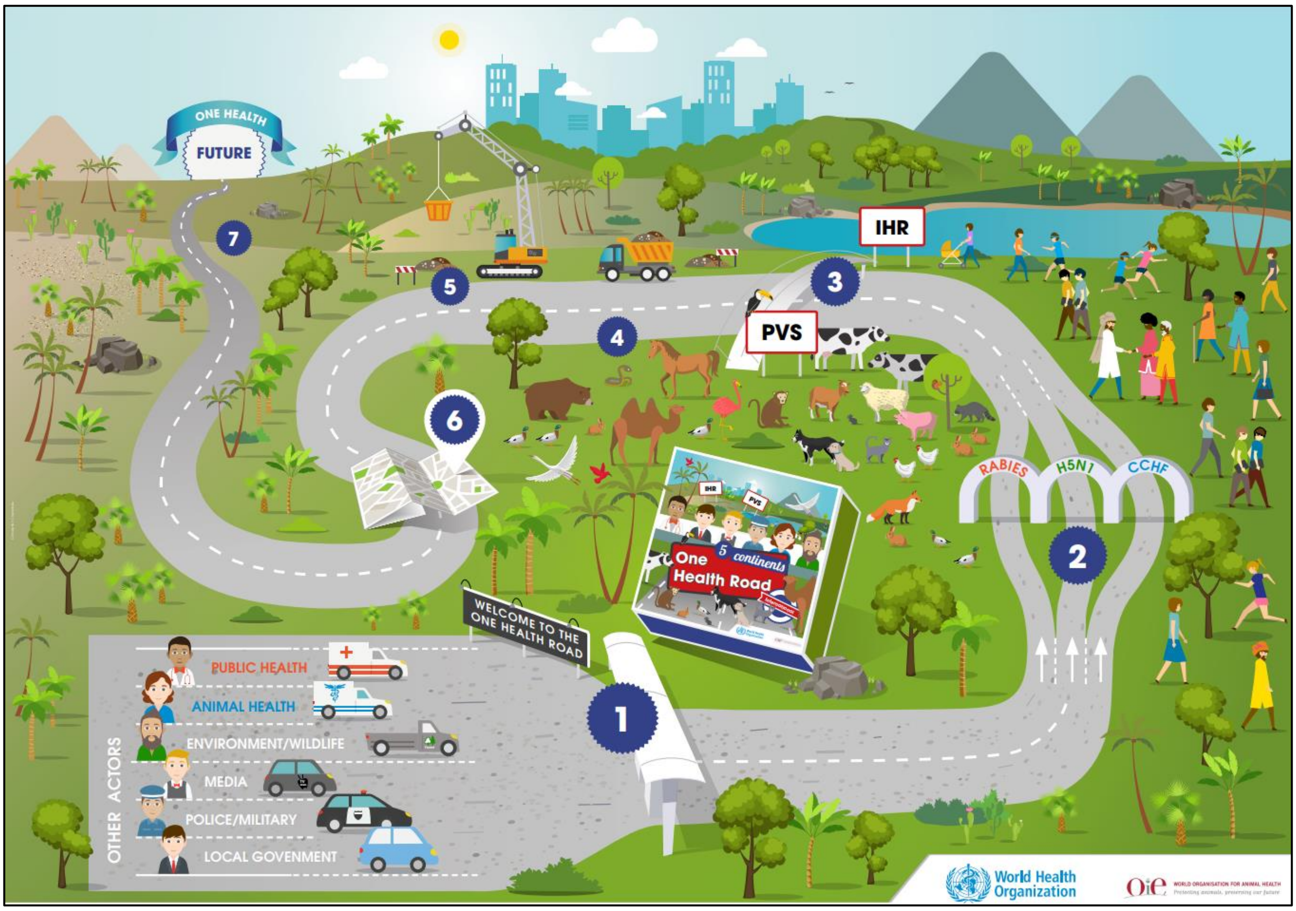

Supplement: S1 Appendix — (PDF) [file pone.0245312.s001.pdf]
