# Supplementary material for: IHR-PVS National Bridging Workshops, a tool to operationalize the collaboration between human and animal health while advancing sector-specific goals in countries
Source: PLoS One. 2021 Jun 1;16(6):e0245312. doi: 10.1371/journal.pone.0245312 (PMC8168895; doi:10.1371/journal.pone.0245312)
Supplement: S2 Appendix — (PDF) [file pone.0245312.s002.pdf]

WORLD ORGANISATION FOR ANIMAL HEALTH

World Health Organization

Facilitator Manual

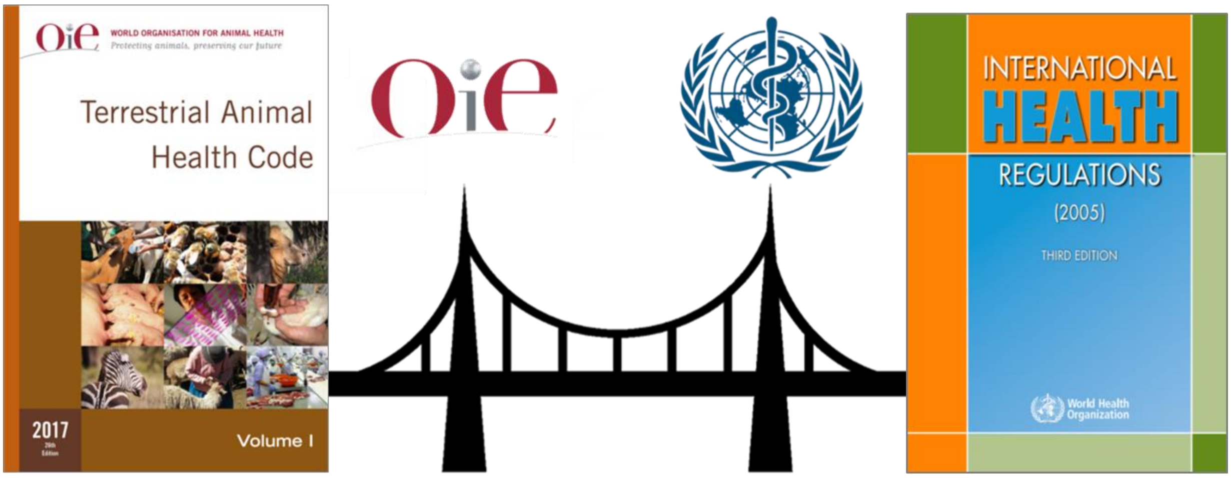

National IHR and PVS Pathway Bridging Workshop

The Road to One Health

(Version 6)

TABLE OF CONTENTS

TABLE OF CONTENTS 3

Purpose of this manual 4

ABBREVIATIONS & ACRONYMS 5

INTRODUCTION 6

Background 6

Expected outcomes of IHR-PVS National Bridging Workshops 7

OVERALL PROCESS 8

LIST OF MATERIAL 10

Logistics 10

Participant material 10

Workshop material 11

PRE-WORKSHOP MEETING 13

SESSION 1: ONE HEALTH CONCEPT & NATIONAL PERSPECTIVES 14

Workshop approach & methodology 14

Movie 1: Tripartite One Health collaboration & vision 14

Presentations: National public health & animal health services 15

Movie 2: Driving successful interactions 15

SESSION 2: THE ROAD TO ONE HEALTH – INTERACTIONS & GAPS 16

Exercise 1: Case studies & assessment of levels of collaboration 16

Plenary: Restitution of exercise 1 18

SESSION 3: BRIDGES ALONG THE ROAD TO ONE HEALTH 19

Movie 3: IHR monitoring & evaluation framework 19

Movie 4: PVS Pathway 19

Movie 5: IHR-PVS Bridging 20

Exercise 2: Mapping of gaps on the IHR-PVS matrix 20

Plenary: Discussion 22

SESSION 4: EXTRACTION OF ASSESSMENT RESULTS 23

Exercise 3: Extraction of assessment results 23

SESSION 5: JOINT ROAD PLANNING 26

Exercise 4: Identification of joint activities 26

SESSION 6: FINALIZATION OF THE JOINT ROAD-MAP 28

Exercise 5: Fine-tuning of joint activities 28

Exercise 6: World café 31

Exercise 7: Prioritization vote 32

SESSION 7: WAY FORWARD 33

Exercises to draw the way forward 33

OTHER INSTRUCTIONS 34

Tips for working group exercises 34

Workshop evaluation 34

USB stick 34

EXAMPLES OF EXPECTED WORKSHOP OUTPUTS 35

Output 1: Levels of collaboration assessed for key technical areas 35

Output 2: Objectives and actions identified per technical areas 36

Output 3: Prioritisation of objectives 41

APPENDIX 42

Annex 1: Workshop standard agenda 42

Annex 2: Examples of case study scenarios 45

Annex 3: Evaluation questionnaire 47

FACILITATOR CHECK-LISTS 49

PURPOSE OF THIS MANUAL

The purpose of this manual is to provide all necessary information for the facilitation of an IHR-PVS National Bridging Workshop. The manual describes the overall structure of the workshop and specifies in detail the objectives, tools, instructions and expected outcomes for each individual session. This document is to be used by NBW facilitators throughout the process to ensure that the methodology is well followed.

ABBREVIATIONS & ACRONYMS

AH	Animal Health
AI	Avian Influenza
CCHF	Crimean-Congo Hemorrhagic Fever
FAO	Food and Agriculture Organization of the United Nations
FELTP	Field Epidemiology and Laboratory Training Program
FP	Focal Point
HQ	Headquarters
IHR	International Health Regulations (2005)
IT	Information technology
JEE	Joint External Evaluation
LIMS	Laboratory Information Management System
MEF	Monitoring and Evaluation Framework
MoA	Ministry of Agriculture
MoH	Ministry of Health
NAP	National Action Plan
OIE	World Organisation for Animal Health
PH	Public Health
PVS	Performance of Veterinary Services
Q&A	Questions & Answers
SOP	Standard Operating Procedures
TOR	Terms of Reference
WHO	World Health Organization

INTRODUCTION

BACKGROUND

The World Health Organization (WHO) and the World Organisation for Animal Health (OIE) are the two main international organizations responsible for proposing references and guidance for the public health and animal health sectors respectively. Working in close collaboration with FAO, WHO and OIE have been active promoters and implementers of an intersectoral collaborative approach among institutions and systems to prevent, detect, and control diseases among animals and humans. They have developed various frameworks, tools and guidance material to strengthen capacities at the national, regional and global levels.

- WHO Member States adopted a legally binding instrument, the International Health Regulations (IHR, revised in 2005), for the prevention and control of events that may constitute a public health emergency of international concern. Through these regulations, States Parties are required to **develop, strengthen and maintain** minimum national core public health capacities to **detect, assess, notify and respond to public health threats** and as such, should implement plans of action to develop and ensure that the core capacities required by the IHR are present and functioning throughout their territories. Various assessment and monitoring tools have been developed by WHO such as the **IHR Monitoring and Evaluation Framework (MEF)**, which includes *inter alias* the Annual Reporting Questionnaire for Monitoring Progress and the Joint External Evaluation (JEE) Tool.

- The OIE is the intergovernmental organization responsible for developing standards, guidelines and recommendations for animal health and zoonoses; these are mainly laid down in the OIE Terrestrial and Aquatic Animal Codes and Manuals. In order to achieve the sustainable improvement of national Veterinary Services' compliance with those standards, in particular on the quality of Veterinary Services. The OIE has developed the **Performance of Veterinary Services (PVS) Pathway**, which is composed of different tools to assist countries to objectively assess and address the main weaknesses of their Veterinary Services.

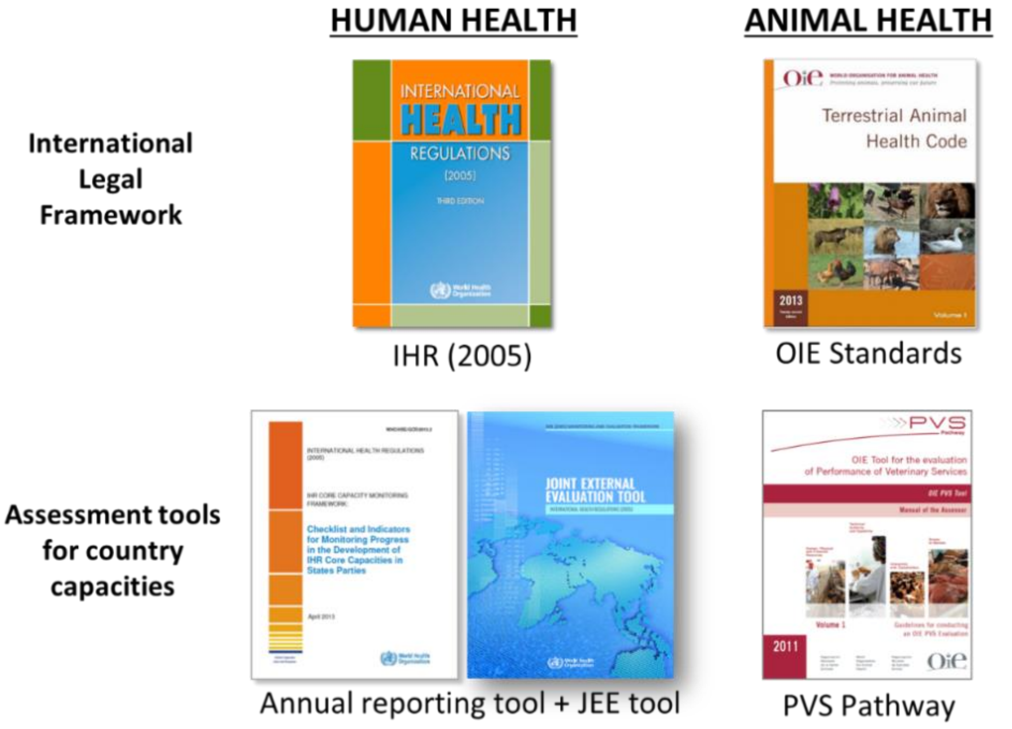

The use of WHO IHR monitoring tools and OIE PVS Pathway would result in a **detailed assessment of the existing forces and gaps**, with **better alignment of capacity building approach and strategies** at country level between the human and animal health sectors. The IHR-PVS National Bridging Workshops (NBW) enable countries to further explore possible overlapping areas addressed in the OIE and IHR capacity frameworks and develop, where relevant, appropriate bridges to facilitate coordination. A structured approach using user-friendly material, case studies and group exercises enables the identification of synergies, review of gaps and the definition of operational strategies to be used by policy makers for concerted corrective measures and strategic investments in national action plans for improved health security.

EXPECTED OUTCOMES OF IHR-PVS NATIONAL BRIDGING WORKSHOPS

The main objective of the NBW is to provide an opportunity to human and animal health services of hosting countries to review their current collaboration gaps in key technical areas and to develop a joint road-map of corrective measures and strategic investments to improve the work at the animal-human interface in the prevention, detection and control of zoonotic diseases.

Expected outcomes of the workshop:

- Increased awareness and understanding on the IHR-MEF and the OIE PVS Pathway, their differences and connections;
- Understanding of the contribution of the veterinary services in the implementation of the IHR (2005) and how the results of the PVS Pathway and IHR-MEF can be used to explore strategic planning;
- Diagnosis of current strengths and weaknesses in the collaboration between animal and human health services for key technical areas;
- Identification of practical next steps and activities for the development and implementation of joint national roadmap to strengthen collaboration and coordination.

OVERALL PROCESS

The workshop uses an interactive methodology and a structured approach with user-friendly material, case studies, group exercises, videos and facilitation techniques. The workshop is made of seven sessions that are structured in a step-by-step process from gap identification to action planning and validation of a joint roadmap for the improvement of the collaboration between the public health and animal health sectors.

Session 1 - Setting the scene: The first session sets the scene by providing background information on the One Health concept and the subsequent tripartite OIE-WHO-FAO collaboration. It is followed by comprehensive presentations from both Ministries on the national public and animal health services. A second documentary provides concrete worldwide examples of fruitful intersectoral collaboration, showing how the two sectors share a lot in terms of approaches, references and strategic views (total duration: **1h40**).

Session 2 - Identification of collaboration gaps: Participants are split in several working groups, each with a case study scenario. Participants discuss the management of zoonotic diseases, identify areas of convergence, evaluate the level of collaboration between the different sectors for key technical areas and identify the main gaps (total duration: **3h30**).

Session 3 - IHR-PVS tools and their bridging: The tools from both sectors (IHR MEF, JEE, PVS) are presented. Joint areas and activities identified for each case study are mapped onto a giant matrix consisting of the indicators of the IHR MEF and of the PVS Pathway. This process enables participants to visualize the gaps identified in each essential capacity and to distinguish disease-specific vs systemic gaps. This will also help determine which technical areas the following sessions will focus on (total duration: **2h30**).

Session 4 - Extraction of assessment results: Participants explore the improvement plans already proposed in the respective assessments (IHR annual reporting, JEE, PVS Evaluation, etc.), extract relevant sections and identify what can be synergized and improved jointly (total duration: **2h00**).

Session 5 - Joint road-planning: Results obtained from the case studies and from the assessment reports are used to develop a realistic and achievable road-map to improve the collaboration between the sectors (total duration: **2h30**).

Session 6 - Finalization of the joint road-map: Activities are fine-tuned and, through a world-café exercise, participants contribute to all technical areas to consolidate the joint-road map by making sure it is harmonized, concrete and achievable (total duration: **3h00**).

Session 7 - Way forward: the last session draws the way forward by identifying the next steps and by linking the developed road-map with other mandated plans such as the National Action Plan for Health Security. This is also where any need from the country can be addressed. This will depend greatly on the current status of the country in terms of IHR-MEF and on their level of One Health capacity.

The workshop uses a road analogy (The Road to One Health), and its process can be summarized with the following figure:

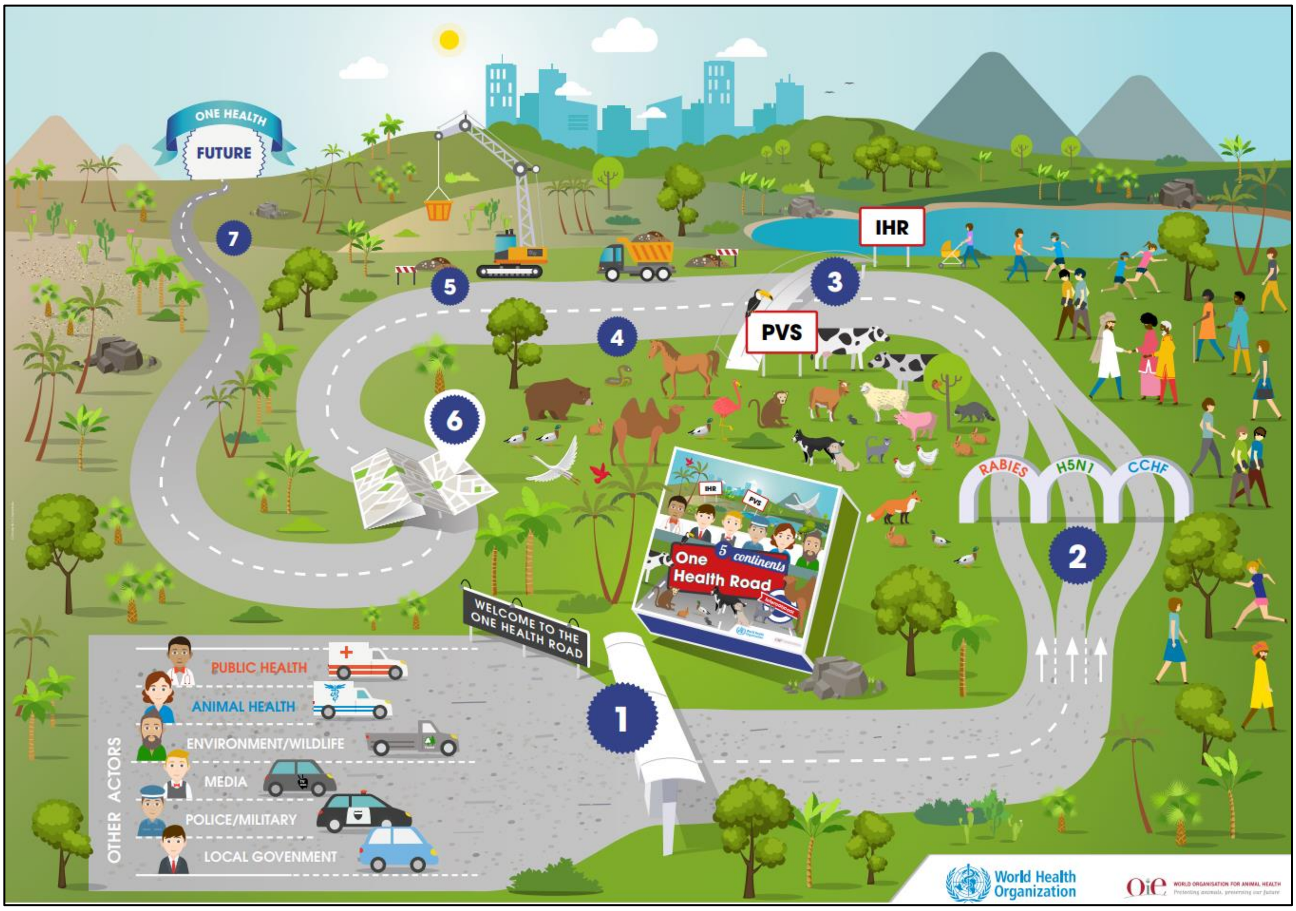

LIST OF MATERIAL

LOGISTICS

Necessary logistics at the venue for the workshop include:

-1 large meeting room able to accommodate 60-80 participants with:

-Computer

-Projector and screen

-Audio system and 3 microphones

-5 flip charts

-2 small meeting rooms for working group session

-Translation services if needed

PARTICIPANT MATERIAL

All participants should be given a workshop folder containing the following items:

-Agenda

-Concept Note

-List of participants

-Participant badge

-**NBW Participant Handbook:** this handbook provides all the necessary information for participants (background, exercise instructions, workshop material, tips, etc.) as well as the main slides from the workshop videos, and space to write notes or the results of their exercises. It should be printed in colors. Make sure the version of the Participant Handbook is the latest, and compatible with the version of this Manual (version 6).

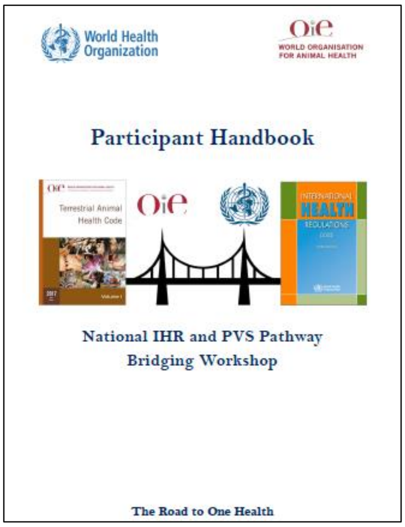

WORKSHOP MATERIAL

Special print-outs (provided by WHO HQ)

These print-outs require a specific format and should be prepared in the early stages of the organization of the workshop.

Road-lane arrow A0 poster (x5)

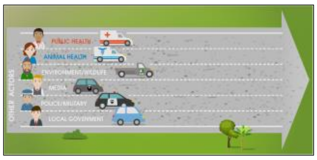

Gap cards (x75)

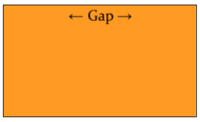

Recommendation cards (x75)

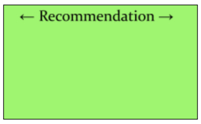

A1 IHR-PVS matrix posters (x5)

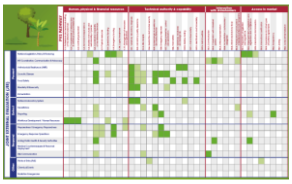

Technical cards (5 decks of 45 cards)

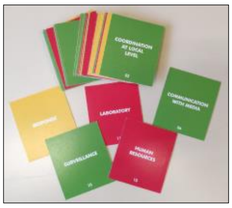

Objective cards (x25)

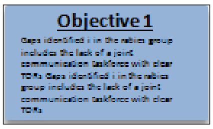

Activity cards (x75)

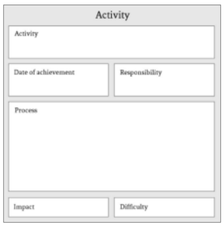

Session 2 report sheet (x5)

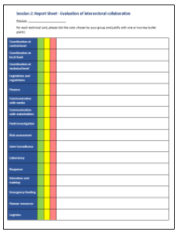

Handbook technical table (15)

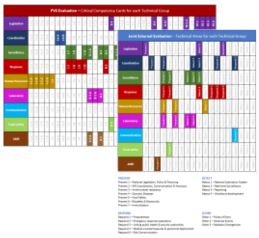

Workshop poster (x1)

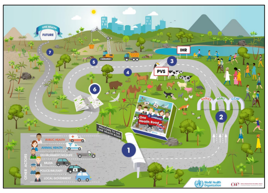

Normal print-outs (to be printed on-site)

These print-outs can be printed in regular A4 format and can be printed in-country:

Participant Handbook (1 pax, color)

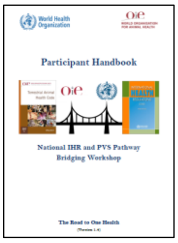

Case-study scenario (x15)

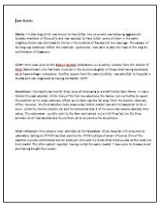

PVS country reports (x15)

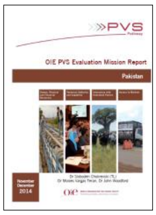

JEE country report (x15) (self-evaluation if no JEE)

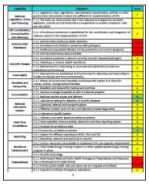

Evaluation Form (1 pax)

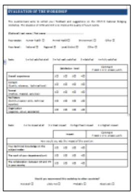

All the material for the workshop (country reports excluded) can be downloaded at:
www.bit.ly/NBWMaterial

Procurement (provided by HQ)

Large standing IHR-PVS matrix

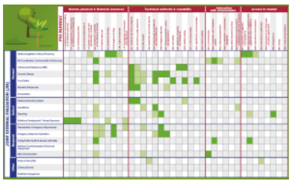

Black marker pens (x10)

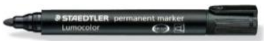

Black fine-point marker pens (x20)

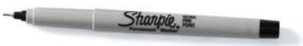

Round stickers (x250 for three colors)

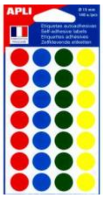

Blue-tack (4 tablets of 80pcs)

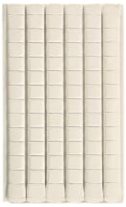

Post-its (6x50 – 3 colors)

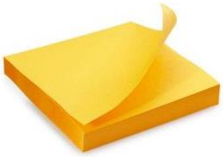

In-country procurement

USB stick (1 pax)

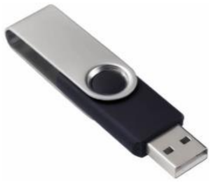

Flip-charts (x5)

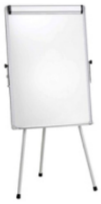

Participant badge (1 pax)

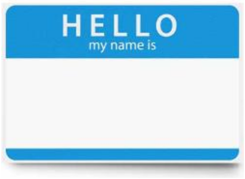

Soft material

-Powerpoint presentation of the workshop methodology (Session 1)
-The five videos for Session 1 and Session 3
-Video of NBW Bhutan to explain the methodology
-Facilitator's Excel sheet for Session 2 results
-Excel Road-map template
-Report template
-Evaluation Questionnaire

PRE-WORKSHOP MEETING

One day before the workshop, a pre-workshop meeting should be organized with:
-Representatives from WHO (country and regional office)
-Representatives from OIE
-Representatives from line Ministries of the country

Check-list for the pre-workshop meeting:

- ☐ Present method and process of the workshop to the line Ministries (use video of NBW Bhutan)
- ☐ Go over and validate the agenda
- ☐ Organize the opening ceremony (who will speak, in what order)
- ☐ Ensure that the minimum number of participants will be present (minimum 25 from each Ministry)
- ☐ Ensure that presentations from the Ministries are ready. Stress that the presentations should focus on the structure of each services, their key activities in terms of One Health, and their challenges. They should not be a lecture on One Health concept, or the PVS Pathway or the IHR.
- ☐ Fine-tune and validate the case studies
- ☐ Tell Ministry representatives that they will need to come up with a detailed list of working groups for each disease by lunchtime of day 1
- ☐ Tell Ministries that they will be taking the full leadership of session 7 on the way forward. This can include a presentation/discussion of how results will be included in other mandated plans (ex: NAPHS, One Health strategic plans, etc). The aim is to show that the Ministries take full ownership of the road-map and leadership of its implementation
- ☐ Talk about the workshop report that will be drafted by WHO and OIE and reviewed by line Ministries. Stress that unless it is clearly requested from their part, the final report is public and will go on WHO's website.
- ☐ Ask if Ministries have any other needs to address during the workshop

The day before the workshop:

- ☐ Use the checklist on page 49 of this manual to ensure that all the material is ready
- ☐ Visit the meeting rooms and ensure that all facilities and equipment are available (projector, screen, microphones, audio system for both microphone and computer).
- ☐ Ensure that **both** OIE and WHO logos are on the agenda and the workshop banner and at the same level of importance
- ☐ Ensure movies 1 and 2 are ready for Session 1

SESSION 1: ONE HEALTH CONCEPT & NATIONAL PERSPECTIVES

Objective: Session 1 sets the scene of the workshop by providing background information on the One Health concept and the subsequent tripartite OIE-WHO-FAO collaboration. It is followed by comprehensive presentations from both Ministries on the national public and animal health services. A second documentary provides concrete worldwide examples of fruitful intersectoral collaboration, showing how the two sectors share a lot in terms of approaches, references and strategic views.

WORKSHOP APPROACH & METHODOLOGY

Duration: 15 minutes

Material: Powerpoint presentation entitled: ***NBW Introduction to the methodology***

Workshop facilitator presents the overall approach and methodology of the workshop (*see Overall Process section earlier*). It is important to stress the following points:

- The workshop is not an evaluation;

- The workshop is not a training either;

- The country has the full ownership of this workshop and its outputs. WHO and OIE are only here to facilitate a methodology that has been tested and validated and to create a conducive environment;

- Organizers/facilitators are not coming with the solutions. They can share many experiences from other countries which may or may not be relevant or applied to the current country but ultimately the solutions will come from the participants themselves;

- Presentations will be kept to a minimum and the workshop will mainly revolve around group exercises. Participation from everyone is crucial. More interaction means more success.

MOVIE 1: TRIPARTITE ONE HEALTH COLLABORATION & VISION

Duration: 15 minutes

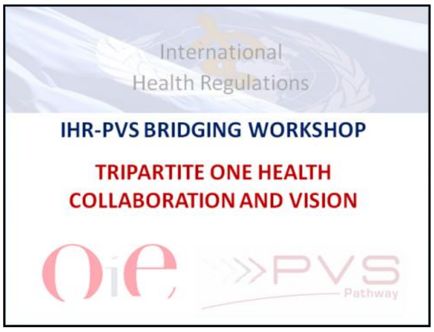

This first documentary video introduces the One Health Concept, its history, rationale and purpose and how it became an international paradigm. The video also introduces the workshop in the global and national context by providing information on the tripartite collaboration between WHO, OIE and FAO.

It is followed by a short Q&A session.

PRESENTATIONS: NATIONAL PUBLIC HEALTH & ANIMAL HEALTH SERVICES

Duration: 40 minutes

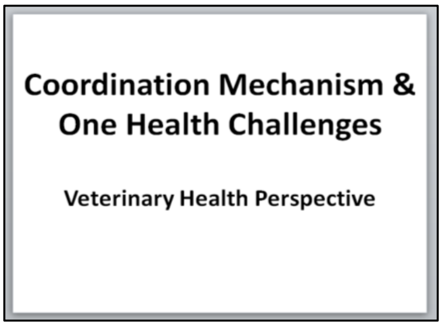

Two presentations are given back to back by representatives of each sector. Presentations should last about 15 minutes each and provide information on:

-their mandate and vision,

-their structure and organigram

-their coordination mechanisms with other sectors

-some examples of existing joint activities.

The presentations should not give a course on One Health or on the PVS or the IHR.

It is followed by a short Q&A session.

MOVIE 2: DRIVING SUCCESSFUL INTERACTIONS

Duration: 25 minutes

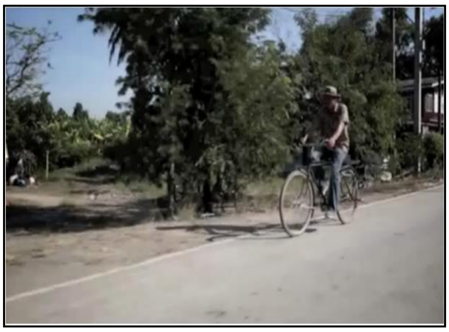

This documentary provides participants with concrete worldwide examples of intersectoral collaboration in addressing health issues at the human-animal interface.

The movie explains that, although there is almost always an inter-ministerial committee, this does not guaranty efficiency of operations at field level. Using the model developed for Rift Valley Fever, an example of a sub-committee framework to help bridge the two sectors at the technical level for all key technical domains is proposed.

It is followed by a short Q&A session as well as a plenary discussion on national experience and lessons learnt.

<u>Example of questions to stimulate the plenary discussion:</u>
-Are there any recent success stories of inter-sectoral collaboration in your country?
-What are the key lessons you have learnt from recent zoonotic outbreaks in terms of intersectoral collaboration needs?

Expected outcomes of Session 1:

- Understanding that intersectoral collaboration between animal and human health sectors happens, but mainly (only?) during outbreaks; with a better preparedness, much more could be done at the human-animal interface.
- Understanding that the two sectors have common concerns and challenges and conduct similar activities. Competencies exist and can be pooled. A collaborative approach is required;
- WHO, OIE and FAO are active promoters of One Health and can provide technical assistance to countries to help enhance inter-sectoral collaboration at the central, local and technical levels.

SESSION 2: THE ROAD TO ONE HEALTH – INTERACTIONS & GAPS

Objective: Discuss the management of zoonotic diseases, identify areas of convergence, evaluate the level of collaboration between the different sectors for key technical areas and identify the main gaps.

EXERCISE 1: CASE STUDIES & ASSESSMENT OF LEVELS OF COLLABORATION

Duration: 2h15

Participants are divided into working groups of mixed participants from both sectors (AH and PH) and from different levels (Federal, Provincial/Regional). Groups are provided with a case study scenario based on diseases relevant to the national context (to be defined before the workshop jointly with representatives from both Ministries).

Examples of case study scenarios used in previous workshops are available in Annex 2.

Organization of working groups

-Groups should have between 10 to 14 participants

-Groups should have an even representation from both sectors

-Groups should have a mix of central/provincial/regional/local representatives

-It is highly recommended to ask key representatives of the country to designate the working groups in advance

-if the number of participants from one sector is not sufficient to have a strict minimum of 5 representatives in each group, then we should reduce the number of groups.

Set-up and material

Facilitator refers to the section *Session 2 working group instructions* in the **NBW Participant Handbook (page 22)** and provides each group with the following material:

Case study scenario

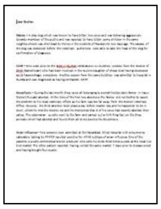

Deck of technical cards

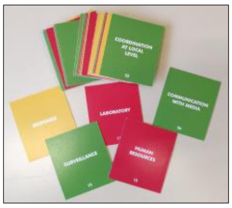

Road-lane arrow poster

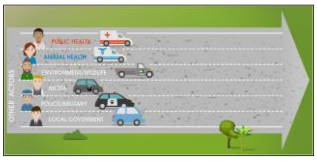

Marker pen

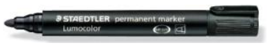

Blue-tack

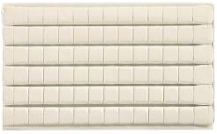

Report sheet

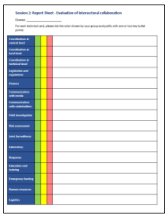

Presentation of the instructions

1. Facilitator refers participants to **page 22** of their **Handbook**
2. Facilitator reads out loud all the instructions
3. **Facilitator demonstrates, using the actual material, what is expected of participants.** It is important to go through the whole process of the exercise
4. Q&A to ensure everyone understood the instructions.

Process

Using experience from previous outbreaks of zoonotic diseases, the groups discuss on how they would have realistically managed these events, and evaluate the level of collaboration between the veterinary and the public health services for 15 key technical areas: coordination, investigation, surveillance, communication, etc. These activities/areas of collaboration are represented by the color-coded technical area cards.

1. Each group identifies a chairman, a rapporteur and a time-keeper
2. Read the scenario and instructions carefully
3. Discuss on past experiences in the management of similar situations
4. Evaluate, for all 15 technical areas, the current level of **collaboration** using the color-coded cards:
    - Very good level of collaboration: **GREEN** card
    - Some level of collaboration: **ORANGE** card
    - Insufficient level of collaboration: **RED** card
5. Put the selected cards on the road-lane arrow and link them to all actors involved using the marker pen.
6. Fill the report-sheet for each technical card by ticking the chosen color and writing the **one or two key points justifying this choice**. These report sheets will be used by other groups in Session 5, therefore please make sure to write in a clear and intelligible manner.

Example of expected results

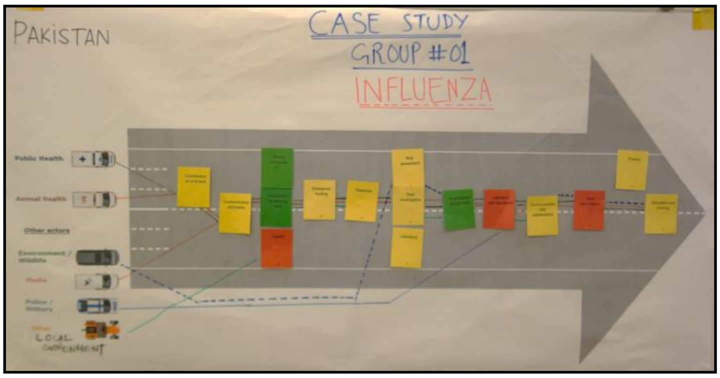

-Example: An intersectoral committee with actors from both services exists and meets both regularly and on an ad-hoc basis when required. Coordination of the response to the outbreak is done jointly at the central level → **Green card** for **'Coordination at high level'**.

-Communication messages are sometimes developed jointly by both sectors but communication plans are not aligned or shared → **Orange card for 'Communication with media'**.

-Each sector carries out its own surveillance and results are rarely shared → **Red card** for **'Surveillance'**.

Answers to frequently asked questions or common mistakes

-The arrow does not necessarily represent a timeline and there is no required order for the cards. The location of the card on the arrow does not matter either, only its color and its link to involved actors is important;

-Only one color for each card should be selected;

-A red card does not necessarily mean that there is absolutely nothing in place, just like a green card does not necessarily mean that everything is absolutely perfect;

-The purpose of the scenario is only to set the context for the discussions, do not be too strict with the details and feel free to drift away from the storyline if needed;

-Examples at the back of the cards are only for guidance. They are not check-lists required to get a green card.

Important: It is essential to ensure that participants understood that they have to evaluate the level of **collaboration**, and not the level of capacity of each sector!

PLENARY: RESTITUTION OF EXERCISE 1

Duration: 1h15

During an ensuing plenary session, each group presents the result of their group work.

Process

1. Facilitator identifies a time keeper who will ensure that each restitution lasts no more than 5 minutes
2. Facilitator calls the rapporteur of the first group to present the results. Rapporteur should not explain the scenario or present all the group members but go straight to the explanation of their results and justify the color that was selected for each technical area card (1 or 2 key reasons justifying the color chosen for each card)
3. Other groups present their result one after the other
4. The discussion is only opened after all groups have presented their results. Participants are invited to speak-up if they disagree with one of the chosen cards. It is possible to change the color of a card if the majority of the participants is in favor of changing it.

Expected outcomes of Session 2:

- Areas of collaboration are identified and joint activities discussed.
- Level of collaboration between the two sectors for 15 key technical areas is assessed.
- The main gaps in the collaboration are identified.

SESSION 3: BRIDGES ALONG THE ROAD TO ONE HEALTH

Objective: Session 3 presents the tools from both sectors (IHR MEF, JEE, PVS) and uses an interactive approach to map the joint areas and activities identified for each case study onto a giant matrix consisting of the indicators of the IHR MEF and of the PVS Pathway.

This process will enable participants to visualize the main gaps identified in each essential capacity and to distinguish disease-specific vs systemic gaps. This will also help participants and facilitators identify which technical areas the following sessions should focus on.

MOVIE 3: IHR MONITORING & EVALUATION FRAMEWORK

Duration: 25 minutes

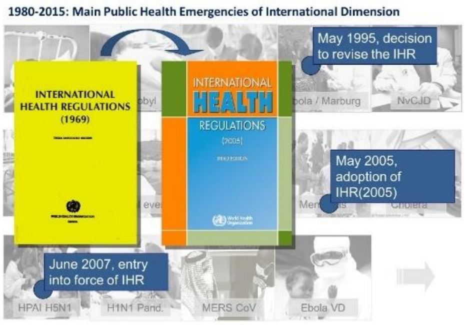

This documentary video presents the International Health Regulations from the initial conception to the recent revisions. It introduces the Monitoring and Evaluation Framework with a special focus on the annual reporting of capacities and the Joint External Evaluation.

It is followed by a Q&A session to ensure that participants have a clear understanding of the purpose and structure of the IHR MEF.

MOVIE 4: PVS PATHWAY

Duration: 25 minutes

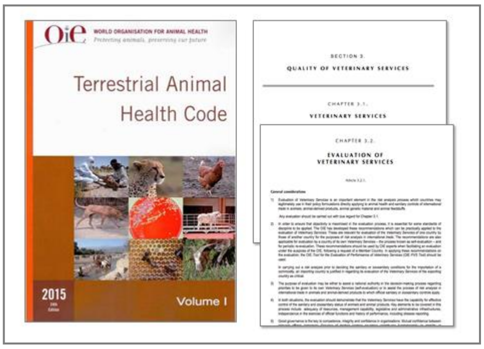

After a quick refresher about the roles and mandate of the OIE, this video presents the PVS Pathway. It explains the different steps of the pathway, their purpose and scope, how they are conducted and what outputs are produced.

It is followed by a Q&A session to ensure that participants have a clear understanding of the purpose and structure of the PVS Pathway.

MOVIE 5: IHR-PVS BRIDGING

Duration: 10 minutes

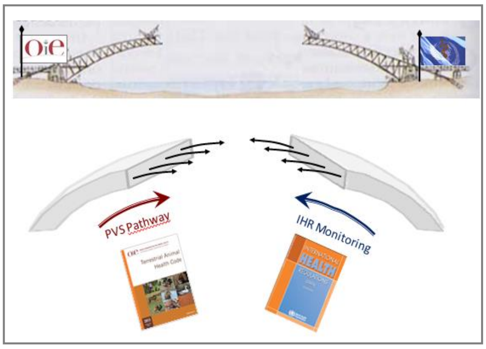

This brief video helps participants to understand how the OIE and WHO tools can be bridged. It shows how the Technical Areas of the IHR MEF can intersect or overlap with the Critical Competencies of the PVS Pathway. It presents the IHR-PVS matrix which will be used in the next exercise.

It is followed by a brief Q&A session.

→ After the last video, facilitator invites participants to open their handbook page 44, and goes through the JEE-PVS comparative table line by line. Pages 45 and 46 are then briefly discussed, just to highlight how the structure of the two tools is similar. This usually really helps participants better understand the similarities and differences between the two tools.

EXERCISE 2: MAPPING OF GAPS ON THE IHR-PVS MATRIX

Duration: 60 minutes (during coffee break)

Set-up and material

The same groups as for the first exercise are kept. Facilitators set up the large IHR-PVS matrix and provide each group with a printed copy of the matrix on an A1 format poster.

Presentation of the instructions

1. Facilitator refers participants to **Session 3 / Exercise 2** on **page 47** of the **Participant Handbook;**
2. **Facilitator demonstrates, using the actual material, what is expected of participants**;
3. Q&A to ensure everyone understood the instructions.

Process

1. Each group gathers the 15 technical area cards that they have selected in the first exercise;
2. Facilitators collect cards number **2**, **3**, **5**, **8**, **9**, **13**, **14**, and **15** from each working group;
3. Each group identifies on their A1 matrix where their seven remaining cards (**1**, **4**, **6**, **7**, **10**, **11**, **12**) fit-in by matching them to their corresponding indicators of the PVS (columns) and IHR (rows);
4. Each group then positions their seven cards on the large matrix using blue-tack;
   At the same time, facilitators position cards **2**, **3**, **5**, **8**, **9**, **13**, **14**, and **15** on the large matrix according to the model presented on the next page.

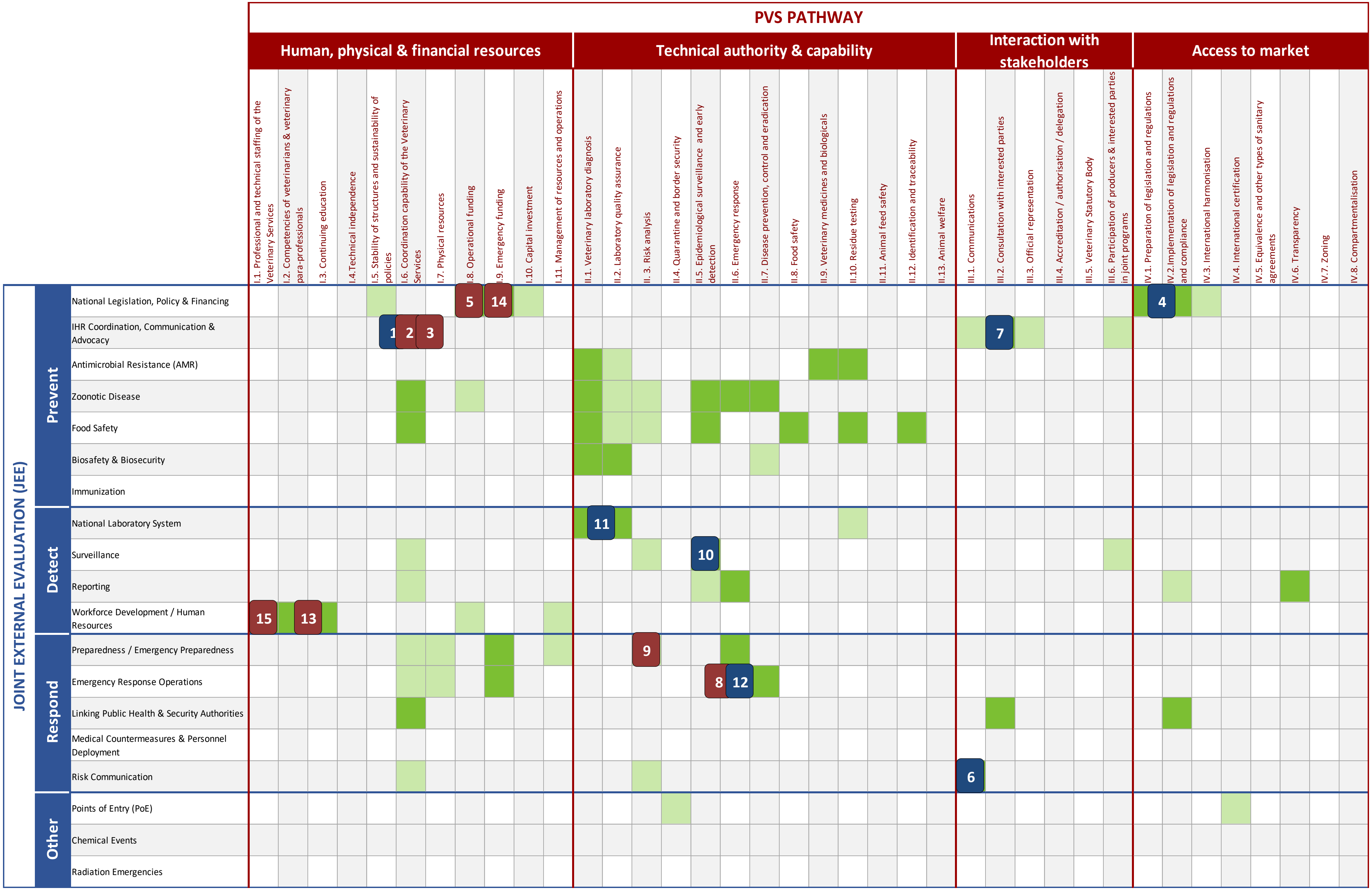
PVS PATHWAY
Human, physical & financial resources
Technical authority & capability
Interaction with stakeholders
Access to market
I.1. Professional and technical staffing of the Veterinary Services
I.2. Competencies of veterinarians & veterinary para-professionals
I.3. Continuing education
I.4.Technical independence
I.5. Stability of structures and sustainability of policies
I.6. Coordination capability of the Veterinary Services
I.7. Physical resources
I.8. Operational funding
I.9. Emergency funding
I.10. Capital investment
I.11. Management of resources and operations
II.1. Veterinary laboratory diagnosis
II.2. Laboratory quality assurance
II. 3. Risk analysis
II.4. Quarantine and border security
II.5. Epidemiological surveillance and early detection
II.6. Emergency response
II.7. Disease prevention, control and eradication
II.8. Food safety
II.9. Veterinary medicines and biologicals
II.10. Residue testing
II.11. Animal feed safety
II.12. Identification and traceability
II.13. Animal welfare
III.1. Communications
III.2. Consultation with interested parties
III.3. Official representation
III.4. Accreditation / authorisation / delegation
III.5. Veterinary Statutory Body
III.6. Participation of producers & interested parties in joint programs
IV.1. Preparation of legislation and regulations
IV.2.Implementation of legislation and regulations and compliance
IV.3. International harmonisation
IV.4. International certification
IV.5. Equivalence and other types of sanitary agreements
IV.6. Transparency
IV.7. Zoning
IV.8. Compartmentalisation
JOINT EXTERNAL EVALUATION (JEE)
Prevent
National Legislation, Policy & Financing
IHR Coordination, Communication & Advocacy
Antimicrobial Resistance (AMR)
Zoonotic Disease
Food Safety
Biosafety & Biosecurity
Immunization
Detect
National Laboratory System
Surveillance
Reporting
Workforce Development / Human Resources
Respond
Preparedness / Emergency Preparedness
Emergency Response Operations
Linking Public Health & Security Authorities
Medical Countermeasures & Personnel Deployment
Risk Communication
Other
Points of Entry (PoE)
Chemical Events
Radiation Emergencies
5
14
4
1
2
3
7
11
10
15
13
9
8
12
6

PLENARY: DISCUSSION

Duration: 30 minutes

A plenary analysis of the outcome is conducted in front of the matrix. Gap clusters are identified and discussed. One key finding is usually that the main gaps are identical for all or most of the working groups, showing that these gaps are not disease-specific but systemic.

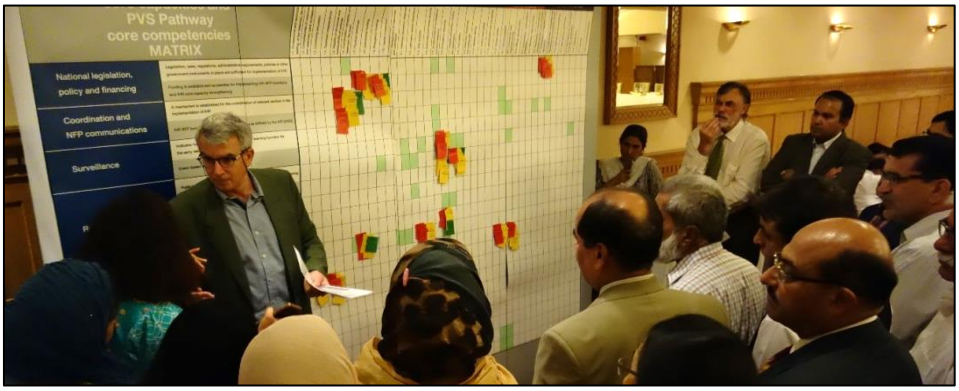

At this stage, the facilitator will propose to select 4 thematic areas where gaps are important and where there is potential for improvement in the near future. These technical areas will be the main focus of the next sessions.

<u>Frequently used thematic:</u>

-Coordination (central/local/technical level);

-Surveillance;

-Response;

-Communication (media and stakeholders);

-Laboratory;

-Note: Legislation & Regulations / Finances: we do not recommend these as the room for improvements is usually limited).

If the number of red or yellow cards is very important, it is possible to combine technical areas that are somewhat associated, for example 'surveillance and laboratory' or 'investigation and response'.

Expected outcomes of Session 3:

- Understanding that tools are available to explore operational capacities in each of the sectors.
- Understanding of the contribution of the veterinary sector to the IHR.
- Understanding of the bridges between the IHR MEF and the PVS Pathway. Reviewing together the results of capacities assessment may help in identifying possible synergies and optimize collaboration.
- Understanding that most gaps identified are not disease-specific but systemic.
- Identification of the technical areas to focus on during the next sessions.

SESSION 4: EXTRACTION OF ASSESSMENT RESULTS

Objective: Explore the improvement plans already proposed in the respective assessments (IHR annual reporting, JEE, PVS Evaluation, etc.), extract relevant sections and identify what can be synergized and improved jointly.

EXERCISE 3: EXTRACTION OF ASSESSMENT RESULTS

Duration: 1h45

Organization of working groups

-Set-up a blank flip-chart for each of the identified technical groups

-Add 5 slots for public health services and 5 slots for veterinary sector on each flip-chart

-Ask participant to sign-up for the group of their choice by adding their name in one of the free slots, but they cannot join a group that already has 5 participants from their sectors

-When all groups have 5 representatives from each sector, remaining participants can join any group they want.

Set-up and material

Facilitator refers to the section *Session 4 working group instructions* in the **NBW Participant Handbook** and provides each group with the following material:

Flip-chart

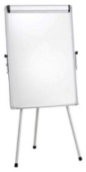

Gap cards (x12)

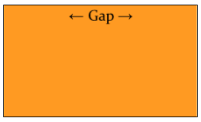

Recommendation cards (x12)

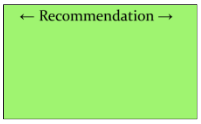

JEE report (x3)

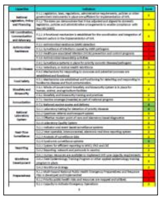

PVS Evaluation report (x3)

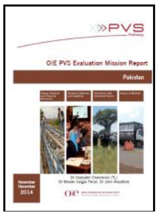

Indicator tables (x6)

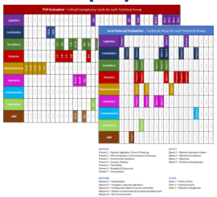

Fine point markers

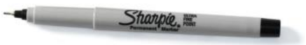

Blue-tack

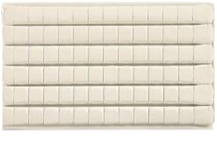

Presentation of the instructions

1. Facilitator refers participants to **Session 4 / Exercise 3** on **page 48** of the **Participant Handbook**;

2. **Facilitator demonstrates, using the actual material, what is expected of participants**. It is important to go through the whole process and explain each item.

3. Q&A to ensure everyone understood the instructions.

Process

1. Using the **indicator crossing table**, participants identify the sections from the PVS Evaluation, the annual reporting and the JEE which may be relevant to their technical area;

2. For each sector, participants extract the six main gaps (12 in total) reported in the assessment documents and write them on the **Gap cards**;

3. For each sector, participants extract the six main recommendations (12 in total) identified in these assessments and report them on the **Recommendation cards**;

4. Each group positions the **Gap** and **Recommendation** cards on the flip-chart using **blue-tack** and the following template:

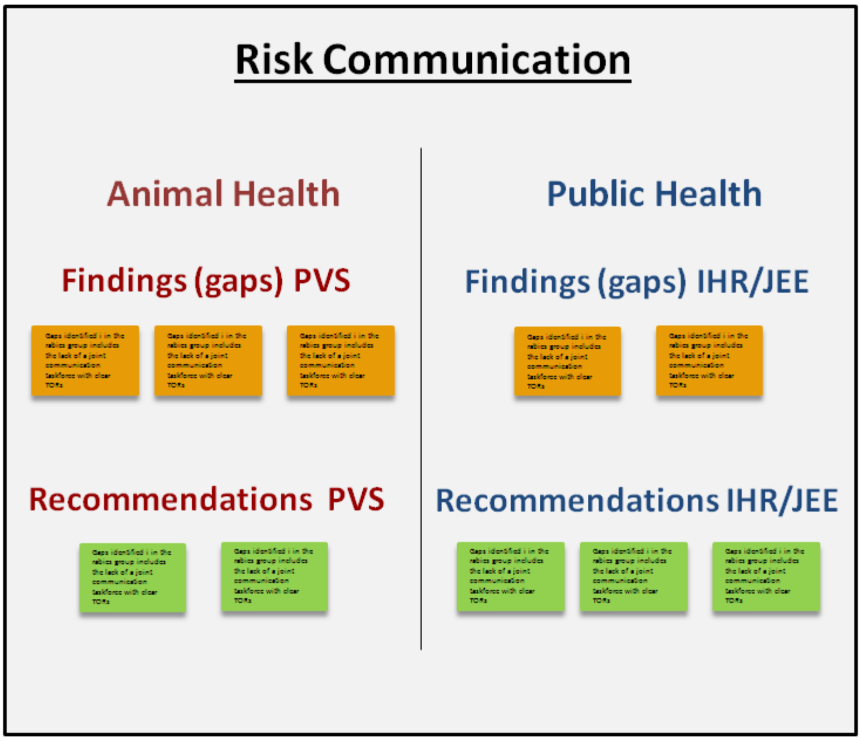

Important: It is possible and advised to include in this session any other relevant assessments that the country may have conducted: PVS Gap analysis, Laboratory mapping tool, Epi mapping tool etc.

Answers to frequently asked questions or common mistakes

-Focus should be made on gaps/recommendations that are **somewhat relevant to One Health**. If a gap or recommendation is entirely specific to one sector it is not relevant.

-Participants should focus only on their technical area and **avoid overlap** with thematics addressed by other groups.

-Avoid the situation where veterinarians work on their report and public health service work on theirs. This is a good opportunity for each sector to know about the other sector and open their assessment reports. The group should go through all the tools together.

Important: There is no restitution of the working groups for this session because it is only a preliminary step for Session 5.

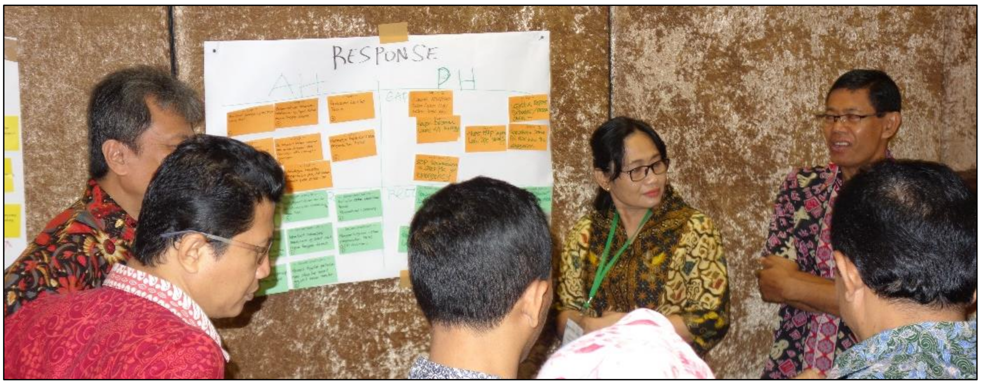

Expected outcomes of Session 4:

- Good understanding of the assessment reports for both sectors, their purpose and their structure.
- Main gaps relevant to each technical area have been extracted.
- Main recommendations from existing reports have been extracted.
- A common understanding of the effort needed starts to emerge.

SESSION 5: JOINT ROAD PLANNING

Objective: use the results obtained from the case studies and from the assessment reports to develop a realistic and achievable road-map to improve the collaboration between the sectors.

EXERCISE 4: IDENTIFICATION OF JOINT ACTIVITIES

Duration: 2h30

Organization of working groups

The same groups (per technical area) as for the previous exercise are kept.

Set-up and material

Flip-chart

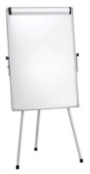

Fine point markers

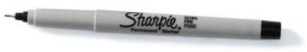

Session 2 results

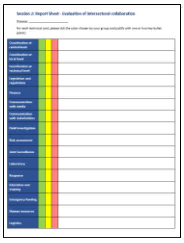

Session 4 results

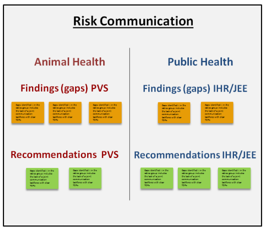

Technical card(s) relevant to each technical group

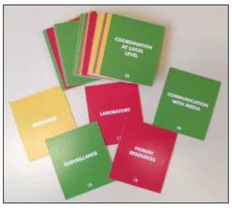

→ Facilitator refers participants to **Session 5 / Exercise 4** on **page 50** of the **Participant Handbook**.

Process

1. Participants should read fully the instructions before starting, including the good/bad examples on page 51 of the Participant Handbook.
2. Identify **realistic** and **achievable** JOINT ACTIVITIES (minimum 3, maximum 10) that would strengthen the inter-sectoral collaboration and improve performance for your thematic area.
3. Activities must fit the **SMART** criteria (**Specific**, **Measurable**, **Achievable**, **Relevant** and **Time-bound**).

4. The activities need to be clearly understandable (What? How?) by just reading them, without requiring further information.

5. Write the activities on the flip-chart and discuss them with the facilitating team.

6. Fine-tune the activities according to the outcomes of the discussion.

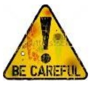

Activities should not be defined only based on gaps identified in the assessment reports. Use all sources of information, including:

→ The gaps identified in the case-study exercise (using the session 2 report-sheet)

→ The gaps and recommendations found in the assessment reports (JEE, PVS, etc.)

→ The discussions held during the workshop so far

→ And most importantly, your **<u>personal experience</u>**!

Answers to frequently asked questions or common mistakes

-Activities need to be clear and accurate. Do not mistake objectives and activities. For example, "capacity building of communication staff" is not an activity, but "3-day training for 25 communication staff" is.

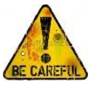

“Enhance”, “improve”, “harmonize”, “standardize” → Objective
“Create”, “Conduct”, “Produce”, “Develop”, “Prepare”, “Draft” → Activity

-Activities should be clear enough so that someone who is not from your group can understand **precisely** what you will do and how you will do it, without the need for any further explanation.

-Use existing resources and material nationally and internationally: avoid developing big things that already exist elsewhere (ex: assessment tools, training curricula, etc).

-Page 51 of the Participant Handbook gives concrete bad and good examples of activities.

-The back of the technical cards relevant to each group could contain some example of activities to use.

Important:

- It is essential to understand that you are **<u>not</u>** aiming at improving each sector, but that you are aiming to improve the **<u>collaboration</u>** between the two.
- Activities should be achievable: it is better to plan for little steps and to do them, than to plan for big leaps and to stand still!
- Make sure the activities are **SMART** (**Specific**, **Measurable**, **Achievable**, **Relevant** and **Time-bound**).

Expected outcome of Session 5:

- Clear and achievable activities are identified to improve inter-sectoral collaboration between the two sectors for all technical areas selected.

SESSION 6: FINALIZATION OF THE JOINT ROAD-MAP

Objective: To have all participants contribute to all technical areas and to consolidate the joint-road map by making sure it is harmonized, concrete and achievable.

EXERCISE 5: FINE-TUNING OF JOINT ACTIVITIES

Duration: 1h30

Organization of working groups

The same groups (per technical area) as for the previous exercise are kept.

Set-up and material

Flip-chart

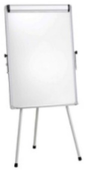

Objective cards (x3)

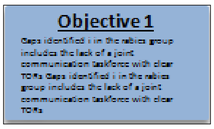

Activity cards (x10)

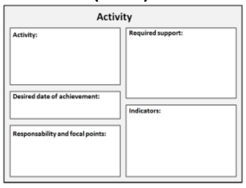

Blue-tack

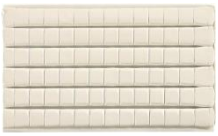

Fine point markers

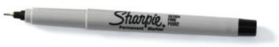

Red stickers (x30)

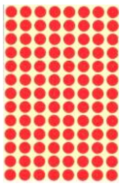

Blue stickers (x30)

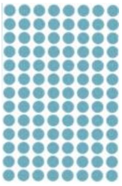

Presentation of the instructions

1. Facilitator refers participants to **Session 6 / Exercise 5** on **page 53** of the **Participant Handbook**;
2. **Facilitator demonstrates, using the actual material, what is expected of participants**. It is important to go through the whole process and explain each item.
3. Q&A to ensure everyone understood the instructions.

Process

1. Participants discuss with the facilitators to group the activities together under 1-to-3 specific objectives. Write the objectives on the **Objective cards**.

2. For **each** activity, fill up an **Activity card** indicating a desired date of achievement, who is responsible and explaining the **detailed** process of implementation.

3. Position the cards on a flipchart using blue-tack and the template shown on the next page.

4. For each activity, evaluate, using the coloured stickers, the cost of implementation and the level of impact this would have in terms of improvement by following the following scale:

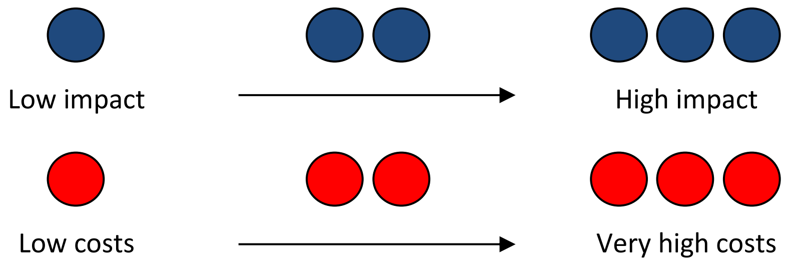

Answers to frequently asked questions or common mistakes

-Results will determine the future road-map, please use good hand-writing and avoid using acronyms.

-The cards must be sufficiently complete and clear, so that someone who is not in the workshop (for example your Minister of Finances) can understand precisely what you will undertake, why, and how you will implement it, by just reading the card. No further explanation should be required.

-Responsibility should be specific. “MoH and MoA” is not a satisfying answer for the box “Responsibility”.

-Use existing resources and material nationally and internationally: avoid developing big things that already exist elsewhere (ex: assessment tools, training curricula, etc).

Important:

- Activities should be achievable: it is better to plan for little steps and to do them, than to plan for big leaps and to stand still!
- Make sure the activities are **SMART** (**Specific**, **Measurable**, **Achievable**, **Relevant** and **Time-bound**).

➔ Invite participants to heck the detailed example of expected results and checklists on page 54 and 55 of the Participant Handbook.

Example of expected result

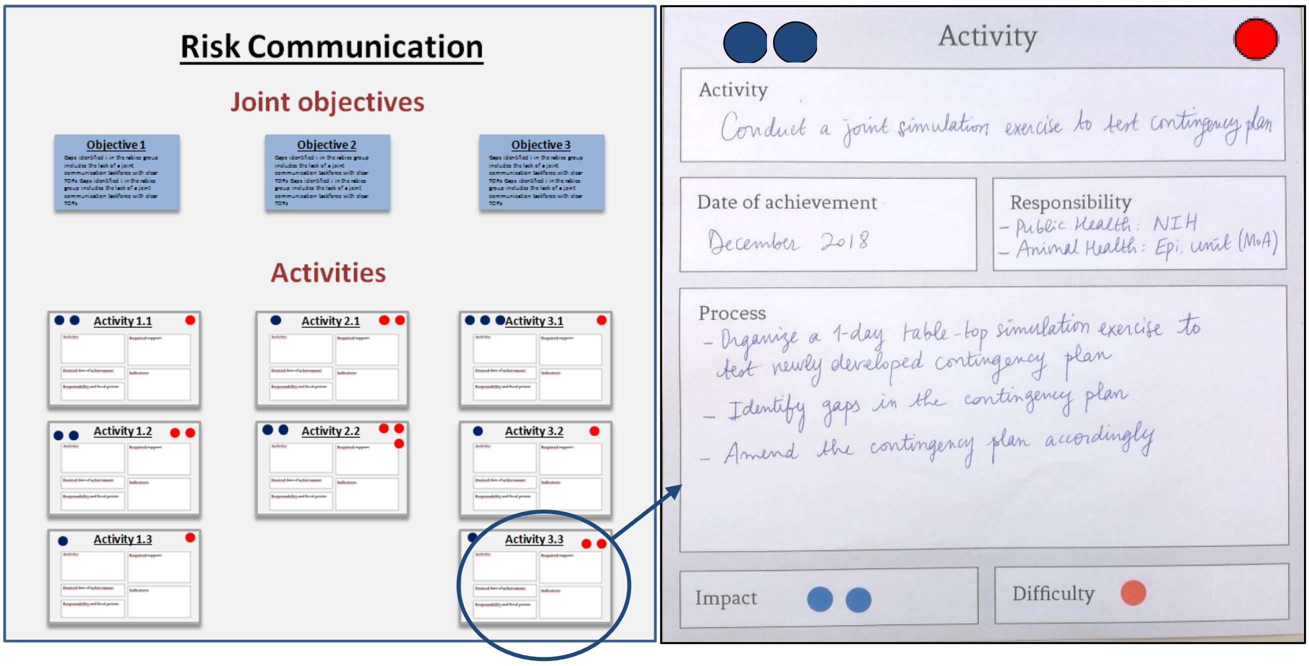

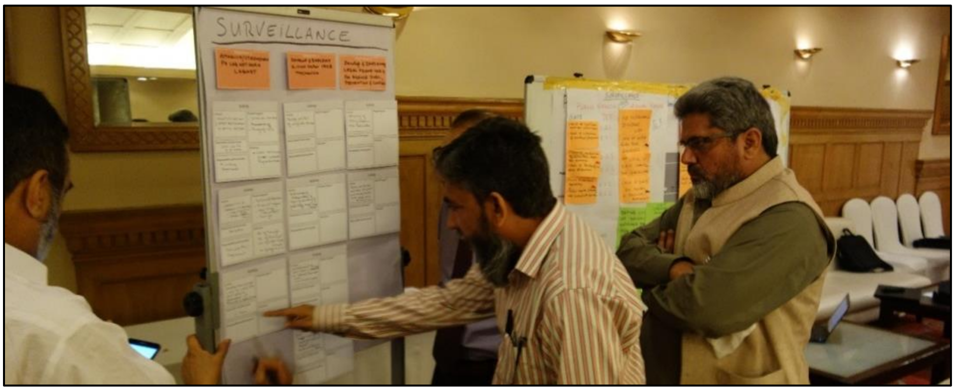

EXERCISE 6: WORLD CAFÉ

Duration: 90 minutes

The World Café exercise enables participants to contribute to the action points of all technical areas. Each group will rotate through the other groups to make comments or ask for further information by leaving post-it notes. World café Instructions will be given by the facilitators.

Process

1. Each group identifies one note-taker;
2. The note-taker stays at his/her board throughout the whole world café. The rest of the group will rotate from board to board;
3. The facilitator sets a 12-minute timer on the screen;
4. Members of Group 1 go to the board of Group 2 (and so on); they read the objectives and activities and should be able to understand them fully (What? How?). If they ask the note-taker of Group B for clarifications, it means the activity is not clear enough and needs to be further clarified.
5. Members provide their feedback (suggestions, edits, additions, re-evaluation of impact/difficulty etc) by writing them on post-its that they stick on the objective/activity cards.
6. When the timer beeps, groups rotate again (Group 1 now goes to the poster of Group 3 etc.);
7. Steps 3-7 are repeated until participants have had a chance to contribute to all technical areas;
8. Each group goes back to their board. The note-taker gives a summary of the feedback that was collected from the other groups;
9. Each group edits and fine-tunes their objectives and activities to consider the feedbacks received until all post-its have been addressed (20').

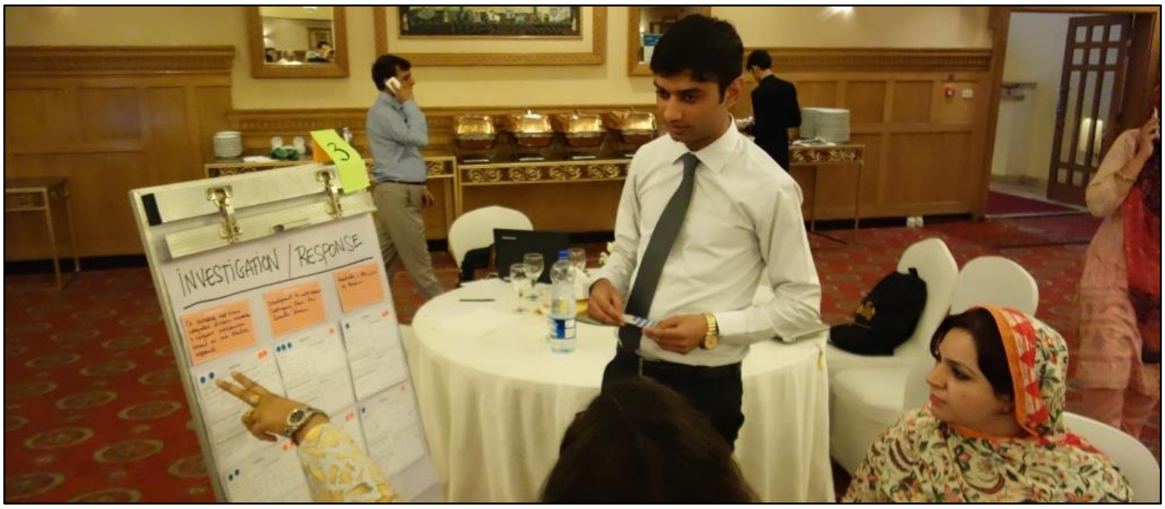

EXERCISE 7: PRIORITIZATION VOTE

Duration: 20 minutes.

Material

White stickers
(x5 per participant)

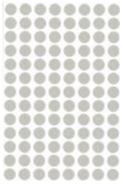

Process

1. Each participant is given 5 stickers;
2. Participants have to put one sticker on the 5 objectives that they believe should be set as high priority (voting for one objective means voting for all the underlying activities it contains).

An alternative is to use an online survey application (such as Google-Forms) as seen below:

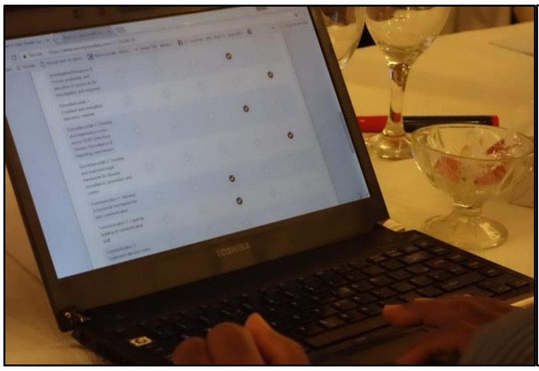

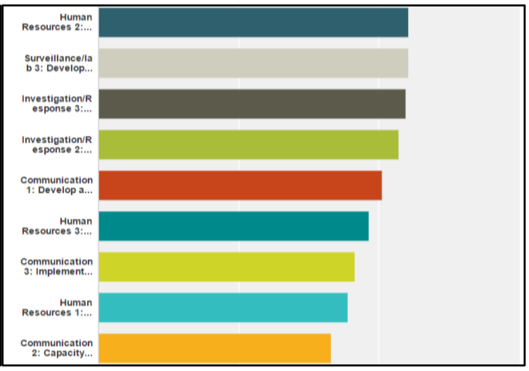

Expected outcomes of Session 6:

- Harmonized, concrete and achievable road-map.
- Buy-in and ownership of all participants who feel that they contributed to all areas of the road-map.
- Prioritization of the activities.

SESSION 7: WAY FORWARD

Objective: the last session draws the way forward by identifying the next steps and by inscribing the developed road-map into other mandated plans such as the National Action Plan for Health Security. This is also where any need from the country can be addressed. This will depend greatly on the current status of the country in terms of IHR-MEF and on the level of One Health capacity.

EXERCISES TO DRAW THE WAY FORWARD

These are some examples of exercises that can be conducted during Session 7:

Linkage with the IHR National Action Plan for Health Security (NAPHS) or other mandated plans

Should the country be in the process or planning to develop their NAPHS, this step is essential. The outcomes of the NBW can feed directly into the NAPHS. Doing so will enable to ensure a proper follow-up on the road-map, guarantee political commitment to its implementation, and provide funding opportunities.

Example 1: country presents the status of their NAPHS and an exercise is conducted to see how activities of the road-map can fit and be included in the NAPHS (Pakistan).

Example 2: country presents their One Health Strategic Plan and an exercise is conducted to see how activities of the road-map can fit and be included in it (Bhutan).

After meeting actions

A final group session can be organized to discuss the next steps and the implementation of identified One Health action. Groups can be asked the following questions: "As a follow up of this meeting what will be the immediate action to improve the coordination for One Health at your level?".

This exercise can be particularly interesting in decentralized countries, in which case it is recommended to organize the groups by province/region so that participants can exchange with their direct counterparts.

Opportunities for other components of the IHR-MEF

It can be interesting for countries to discuss about the next steps to further improve inter-sectoral collaboration and identify opportunities for other components of the IHR-MEF, such as a joint *Simulation Exercise* or a joint *After Action Review.*

Expected outcomes of Session 7: Depends on the country needs and level of advancement in implementation of the IHR-MEF but options can include:

- Linkages with NAPHS.
- Identification of immediate and practical next steps.
- Identification of opportunities for other components of the IHR-MEF.

OTHER INSTRUCTIONS

TIPS FOR WORKING GROUP EXERCISES

- When giving instructions for exercises, always refer participants to the relevant page of their handbook, go through all the instructions and make a full demonstration of what they are being asked to do;
- For each exercise, circulate early throughout all the groups to make sure that the instructions are well understood by all participants;
- Take clear pictures of all outputs produced during the working group sessions;
- Keep all **Objective** and **Activity** cards produced as they will be necessary to compile the final roadmap.

WORKSHOP EVALUATION

On the last day of the workshop, participants are asked to provide their feedback on the workshop using an evaluation questionnaire (Annex 3). This questionnaire is available at the end of the **NBW Participant Handbook**. To ensure that all participants provide feedback it is recommended to give the USB stick (see next point) in exchange for the filled form. The form takes about 10 minutes to fill.

USB STICK

On the last day of the workshop, each participant receives a USB stick which includes all the material used during the workshop, including movies, presentations, documents of references and results from the working groups. Copying all the material on the USB sticks can take a significant amount of time so plan wisely!

EXAMPLES OF EXPECTED WORKSHOP OUTPUTS

OUTPUT 1: LEVELS OF COLLABORATION ASSESSED FOR KEY TECHNICAL AREAS

The following example shows the results obtained from eight working groups (two groups per disease) when they assessed the level of collaboration between the animal health and public health sectors for the 16 key technical areas shown on the left. The level of collaboration uses a three-color code: **Red** (collaboration needs major improvement), **Orange** (some collaboration exists but it should be strengthened) and **Green** (collaboration is excellent).

Technical area (cards)	Rabies	Anthrax	H5N1	Brucellosis	RVF	Score*
Coordination at local Level						10
Finance						10
Communication w/ media						9
Emergency funding						9
Joint surveillance						8
Field investigation						7
Laboratory						6
Response						6
Communication w/ stakeholders						5
Risk assessment						5
Human resources						5
Coordination at technical Level						4
Coordination at high Level						3
Legislation / Regulation						3
Education and training						3

**The score uses a semi-quantitative scale (2 points for a red card, 1 for a yellow card and 0 for a green card).*

OUTPUT 2: OBJECTIVES AND ACTIONS IDENTIFIED PER TECHNICAL AREAS

The following example shows an example of joint road-map developed during a NBW:

Action	Timeline	Cost	Impact	Responsibility	Process
**COORDINATION AT HIGH, TECHNICAL, AND LOCAL LEVELS**					
**Objective 1: Establish high level national mandate for One Health collaboration**					
**1.1 Sign Memorandum of Understanding on mutual collaboration**	February 2019	+	+++	Legal and Technical Departments of MHSP, MARD, and Ministry of Environment	1) Agree to draft MoU at high level<br>2) Set up a working group<br>3) Draft MoU including Animal and Human Health inputs<br>4) Conduct consultations in each sector<br>5) Approve the MoU by all involved parties<br>6) Commence MoU
**1.2 Establish a National Multisectoral Committee on Zoonoses (NMCZ) according to the MoU provisions**	April 2019	+	+++	MHSP, MARD, and Ministry of Environment	1) Establish a working group<br>2) Develop ToR for NMCZ<br>3) Develop a working plan of the NMCZ<br>4) Approve establishment of NMCZ for coordination on zoonotic diseases of common interest in line with ToR
**Objective 2: Enhancing formal One Health coordination authority structures and frameworks**					
**2.1 Review legislation relevant to One Health and control of zoonoses**	December 2019	++	+++	Technical and legal experts from different sectors	1) NMCZ to establish a working group of technical and legal experts from different sectors<br>2) Develop ToRs<br>3) Report to NMCZ periodically<br>4) Final report and recommendations are to be approved by NMCZ
**2.2 Establish joint technical sub-committees for priority zoonoses to develop strategic joint response plan and/or update contingency/control plans**	October 2019	++	+++	National Multisectoral Committee on Zoonoses (NMCZ)	- NMCZ to establish technical sub-committees for specific zoonoses of common interest:<br>• Brucellosis<br>• Anthrax<br>• Tuberculosis<br>• Rabies<br>• Avian Flu<br>• West Nile Fever<br>• Leishmaniasis<br>- Appoint epidemiologists, laboratory, and other relevant specialists in each group<br>- Each sub-committee to develop ToRs<br>- Final reports and recommendations are to be approved by NMCZ
**2.3 Appoint focal points for intersectoral coordination at national and regional levels**	June 2019	++	+++	NMCZ	- Nominate focal points at national and regional levels<br>- Develop ToRs / job description<br>- Focal points become the secretaries of NMCZ<br>- Develop SOPs for focal points<br>- Train focal points

RISK ASSESSMENT, JOINT SURVEILLANCE & LABORATORY					
**Objective 3: Harmonize protocols and sharing of information for joint risk assessment surveillance and laboratory diagnostics of priority zoonotic diseases**					
**3.1 Develop integrated electronic system for routine sharing of data related to priority zoonoses**	May 2019 – September 2020	+++	+++	MHSP, MARD, Institute of Public Health (IPH), Food Safety and Veterinary Institute (FSVI), Medical and Veterinary Faculties	1) Establish working group (8-10 persons)<br>2) Identify the type of information and ways of sharing<br>3) Conduct IT tender<br>4) Develop and test the electronic system<br>5) Implementation
**3.2 Establish national library of SOPs**	December 2019	++	+	IPH, FSVI, FSA	1) SOPs for sample collection, transportation, storage, diagnostic methods, biosafety and biosecurity, waste management, etc.<br>2) Establish working group (8 persons)<br>3) Prepare materials and adopt into Albanian<br>4) Develop/adapt SOPs<br>5) Invite external expert from reference institution for evaluation and consultations<br>6) Test and update SOPs at national and local levels<br>7) Print and distribute SOPs at all sectors involved
**3.3 Develop guidelines for joint surveillance**	March 2019	++	+++	IPH, MARD, FSVI, FSA	1) Establish working group of 12 people<br>2) Translate and adapt existing documents from WHO, OIE, ECDC<br>3) Conduct meetings with Albanian and international experts<br>4) Develop the guidelines<br>5) Test guidelines with all actors involved<br>6) Conduct gap analysis and update the guidelines<br>7) Get approvals from MARD and MHSP<br>8) Publish guidelines electronically and print hard copies for each involved party
**3.4 Develop guidelines for joint risk assessment of zoonotic diseases**	September 2019	++	+++	IPH, MARD, FSVI, FSA	1) Establish a working group of 12 people<br>2) Translate and adapt existing documents from WHO, OIE, ECDC<br>3) Conduct meetings with Albanian and international experts<br>4) Develop the guidelines<br>5) Test guidelines with all actors involved<br>6) Conduct gap analysis and update the guidelines<br>7) Get approvals from MARD and MHSP<br>8) Publish guidelines electronically and print hard copies for each involved party
**Objective 4: Share resources to optimize collective capacity for risk assessment, surveillance and laboratory diagnostics of targeted zoonoses**					
**4.1 Develop capacity of the National Reference Laboratories for zoonoses, considering sharing of human, physical and financial resources**	March 2019	++	+++	Zoonotic Committee, IPH & FSVI	1) Review capacities from both sides involving international expertise<br>2) Identify resources that could be shared<br>3) Reinforce with necessary equipment and materials<br>4) Human resource capacity building:<br>- trainings<br>- exchange of scientific knowledge<br>- study tours
**4.2 Conduct joint vector surveillance and pathogen screening to enable joint risk**	February – November 2019	+++	++	IPH, FSVI, University Faculties	- Identify priority VBDs<br>- Conduct epi and spatial analyses to identify number of samples and sampling locations

**assessment and early detection of vector-borne diseases (VBD)**					- Conduct field work to collect vectors (ticks, flies, mosquitos) and animal samples<br>- Detect infectious agents in vectors and animal/human hosts
**Objective 5: Operationalize and test the joint risk assessment, surveillance and laboratory framework targeting zoonoses**					
**5.1 Organize cascade trainings on joint surveillance and joint risk assessment at the national and local levels**	March - June 2020	++	++	IPH, FSVI	- Establish working group (10 persons) including experts from both institutions and international experts<br>- Develop training materials and training plan<br>- Conduct training for trainers at the national level<br>- Trained trainers to conduct replica trainings at the local level
**RESPONSE, FIELD INVESTIGATION AND EMERGENCY FUNDING**					
**Objective 6: Improve strategic planning for response, field investigation, and emergency funding on priority zoonotic diseases**					
**6.1 Develop a strategic joint response plan for zoonotic diseases**	Six months after establishment of NMCZ (November 2019)	+	+++	NMCZ	- Organize a multisectoral technical meeting at national level to define legal background<br>- Identify focal persons from each sector responsible for drafting the plan<br>- Define ToRs for focal points and experts<br>- Map existing supporting documents (strategic plans, continuous plans, etc.)<br>- Draft a strategic plan<br>- Discuss the draft strategic plan with of all the stakeholders<br>- Finalize the plan<br>- Seek approval by the Government
**6.2 Develop new and update existing contingency and control plans of priority zoonotic diseases (within the framework of strategic joint response plan, as Annexes)**	One year after establishment of NMCZ (April 2020)	++	+++	Technical sub-committees of NMCZ	- Prioritize zoonotic disease (jointly)<br>- Map all existing contingency/control plans from all sectors for each priority zoonosis<br>- Nominate disease technical experts for each priority zoonosis to the respective technical sub-committees of NMCZ (Activity 2.2)<br>- Develop or update existing contingency/control plans which will include joint actions<br>- Structure developed/updated contingency/control plans as Annexes to the strategic joint response plan
**6.3 Develop joint SOPs and other documents supporting developed/updated contingency/control plans**	One year after establishment of NMCZ (April 2020)	+	+++	Technical sub-committees of NMCZ	- Map all existing SOPs/guidelines/recommendations from all sectors for each priority zoonosis<br>- Nominate disease technical experts responsible for development of SOPs<br>- Develop SOPs
**6.4 Conduct joint After Action Reviews for real events caused by priority zoonoses**	3 months after event, when relevant	++	+	National and regional technical staff from MHSP and MARD	- Identify experts from both sectors involved in the event<br>- Request WHO for support<br>- Conduct AAR
**6.5 Review emergency funding arrangements considering joint mechanisms**		+++	+++	NMCZ, MARD, MHSP	- Organize a meeting of experts (finance, MHSP, MARD, technical)<br>- Estimate costs related to response and field investigation<br>- Define the modality and mechanisms to activate joint emergency funds

**Objective 7: Enhance human resource capacities for joint response and field investigation of priority zoonotic diseases**					
**7.1 Develop a joint education module for Master program and post-graduate specialists and conduct trainings on response and field investigation of endemic and emerging zoonotic diseases**	Two years after establishment of NMCZ (April 2021)	+++	+++	NMCZ, IPH, FSVI, FSA, Universities	- Establish working group of experts from different sectors and universities<br>- Develop a curriculum and update it regularly<br>- Get accreditation from statutory bodies and define the related credits<br>- Develop a training plan for specialists<br>- Engage lecturers and trainers<br>- Conduct trainings for specialists<br>- Include the module into the Master programs
**7.2 Develop the joint training program to exercise contingency/control plans and SOPs developed under activities 6.2-6.3**	1.5 years after establishment of NMCZ (November 2020)	+++	+++	NMCZ, IPH, FSVI	- Establish a working group to develop the joint training program/plan<br>- Develop the training program including exercises with increased complexity for each contingency/control plan:<br>• table top exercises<br>• drills<br>• functional exercises<br>• full scale simulation exercises<br>- Identify focal points for exercise program<br>- Involve international experts to help in organization exercises
**7.3 Conduct joint exercises**	Two years after establishment of NMCZ (April 2021)	+++	+++	NMCZ, IPH, FSVI	- Conduct joint exercises on each contingency/control plans for priority zoonoses<br>- Develop content (scenarios, injects, etc.) for each exercise<br>- Prepare the budget and identify number of participants for each exercise<br>- Identify participants for each exercise incl. epidemiologists, laboratory specialists, etc.<br>- Involve international experts to help in delivery of exercises<br>- Revise respective joint strategy response plan, contingency/control plans and related SOPs
**COMMUNICATION**					
**Objective 8: Improve operational capacities for joint One Health risk communication for the management of zoonotic diseases**					
**8.1 Develop a joint risk communication cascade training**	30 November 2020	++	+++	Agricultural University of Tirana (Faculty of Veterinary Medicine), IPH, University of Tirana (Department of Media and Communications)	1) Establish a group of experts to coordinate development of the training program involving national and international experts<br>2) Prepare didactic materials for trainers and participants<br>3) Establish legal procedures to recognize training by Ministry of Education<br>4) Develop criteria for candidate acceptance
**8.2 Deliver a joint risk communication cascade training**	February 2021	++	+++	Agricultural University of Tirana (Faculty of Veterinary Medicine), IPH, University of Tirana (Department of	1) Identify course coordinator<br>2) Identify trainers<br>3) Establish a web workspace<br>4) Selection of modules<br>5) Establish the course timeline<br>6) Course accreditation

				Media and Communications)	7) Deliver the course
**8.3 Develop "One Health Risk Communication" guidelines and SOPs**	December 2019	++	++	MHSP + Agencies;<br>MARD + Agencies;<br>Department of Media and Communication	1) Establish a group of national experts, involve international experts<br>2) Working group to meet monthly<br>3) Prepare the concept of guidelines<br>4) Develop the guidelines<br>5) Approve by both Ministries<br>6) Distribute the guidelines
**8.4 Conduct a table-top exercise to test capacities on risk communication**	March 2021	++	+++	Faculty of Veterinary Medicine, IPH, National Food Authority, Department of Media and Communication	1) Identify TTX coordinator(s)<br>2) Develop content of TTX<br>3) Identify participants and trainers<br>4) Calculate budget<br>5) Deliver TTX<br>6) Evaluate risk communication capacities
**Objective 9: Enhance strategic planning for joint risk communication for management of zoonotic diseases**					
**9.1 Develop a multi-hazard One Health joint risk communication strategy**	November 2019	++	+++	MHSP + Agencies;<br>MARD + Agencies;<br>Prime Minister Office	1) Establish a joint working group<br>2) Mobilize international expert<br>3) Draft a joint RC strategy<br>4) Share the draft strategy with different stakeholders and reach the consensus<br>5) Approve the joint RC strategy at Prime Minister level<br>6) Launch the strategy with all stakeholders<br>7) Include the strategy in the curricula of Universities for zoonotic diseases
**9.2 Develop the joint action plan for the multi-hazard One Health joint risk communication strategy**	January 2020	++	+++	MHSP + Agencies;<br>MARD + Agencies;<br>Ministry of Finance	1) Establish a joint working group involving:<br>• experts who were engaged with development of strategy<br>• international experts<br>• financial experts<br>2) Draft a joint action plan<br>3) Share the joint action plan with different stakeholders and reach the consensus<br>4) Approve the joint action plan at Ministry of Finance and Prime Minister Office<br>5) Share the joint action plan with all stakeholders
**Objective 10: Enhance awareness and positive approach for solutions of problems during management of zoonotic diseases**					
**10.1 Establish joint advocacy group to increase capacities for staff, infrastructure, and budget**	May 2019	+	++	Department of Veterinary Public Health,<br>IPH, Department of Public Health	1) Identify group members<br>2) Develop ToR for the group<br>3) Group to meet regularly<br>4) Develop annual agenda<br>5) Identify key decision makers to meet them<br>6) Use social media for advocacy

Cost: *Low +, Moderate ++, High +++* ***Impact:*** *Low impact +, Moderate impact ++, High impact +++*

OUTPUT 3: PRIORITISATION OF OBJECTIVES

All participants were asked to vote individually via a mobile application and to select which five of the objectives they considered as of highest priority. This is an example of the result obtained:

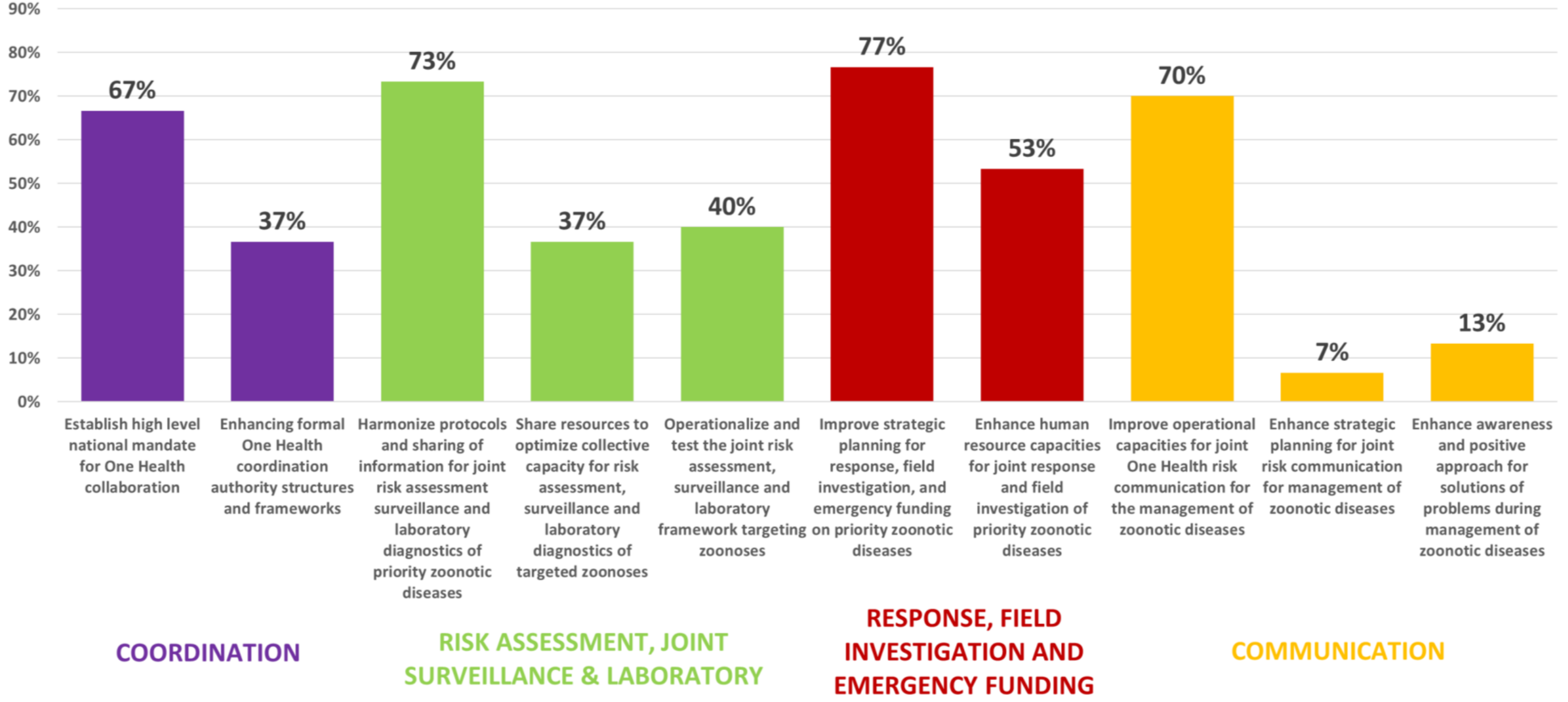

APPENDIX

ANNEX 1: WORKSHOP STANDARD AGENDA

Below is the standard workshop agenda. The agenda for your workshop may be slightly different depending on adjustments made by national authorities.

<table>
<tr><th colspan="2">DAY 1</th></tr>
<tr><td>08:30 – 09.00</td><td>Registration of participants</td></tr>
<tr><td>09.00 – 10.00</td><td><u>Opening Ceremony</u>
• Representative of the Ministries - Public Health + Agriculture (20’)
• Regional Representative of WHO + OIE (20’)
• Introduction of participants (10’)
• Group Picture (10’)
Coffee break (20’)</td></tr>
<tr><td rowspan="2">10.00 – 12.00</td><td><u>Session 1: Workshop Objectives and National Perspectives</u>
The first session sets the scene by providing background information on the One Health concept and the subsequent tripartite OIE-WHO-FAO collaboration. It is followed by comprehensive presentations from both Ministries on the national public and animal health services. A second documentary provides concrete worldwide examples of fruitful intersectoral collaboration, showing how the two sectors share a lot in terms of approaches, references and strategic views.</td></tr>
<tr><td>• Workshop approach and methodology – PPT (10’)
• MOVIE 1: Tripartite One Health collaboration and vision (15’)
• Veterinary Services and One Health – PPT (20’)
• Public Health Services and One Health – PPT (20’)
• MOVIE 2: Driving successful interactions - Movie (25’)</td></tr>
<tr><td colspan="2">Lunch (12:00-13:30)</td></tr>
<tr><td rowspan="2">13.30 – 17.00</td><td><u>Session 2: Navigating the road to One Health</u>
Session 2 divides participants in working groups and provides an opportunity to work on the presented concepts. Each group will have central and provincial representatives from both sectors and will focus on a fictitious emergency scenario.
Using diagrammatic arrows to represent the progression of the situation, groups will identify joint activities and areas of collaboration and assess their current functionality using one of three color-coded cards (green, orange, red).</td></tr>
<tr><td>• Presentation and organization of the working group exercise – PPT (15’)
• Case study - Working groups by disease (120’)
• Restitution (75’)</td></tr>
<tr><td colspan="2">Expected outcomes of Sessions 1 and 2:
• Understanding of the concept of One Health, its history, its frameworks and its benefits.
• Understanding that a lot of areas for discussion and possible improvements do exist and can be operational - not only conceptual.
• Level of collaboration between the two sectors for 16 key technical areas is assessed.
• Collaboration gaps identified for each disease.</td></tr>
<tr><td>17.00 – 18.30</td><td>Facilitators and moderators only:
Briefing Session 3-4-5 and compilation of results from Session 2</td></tr>
</table>

<table>
<tr><th colspan="2">DAY 2</th></tr>
<tr><td rowspan="2">08.30 – 11.20</td><td><u>Session 3: Bridges along the road to One Health</u>
Session 3 presents the tools from both sectors (IHR MEF, JEE, PVS) and uses an interactive approach to map activities identified earlier onto a giant IHR-PVS matrix.
This process will enable to visualize the main gaps, to distinguish disease-specific vs systemic gaps and to identify which technical areas the following sessions will focus on.</td></tr>
<tr><td>• MOVIE 3: IHR Monitoring and Evaluation Framework (25’)
• MOVIE 4: PVS Pathway (25’)
• MOVIE 5: IHR-PVS Bridging (10’)
• Mapping gaps on the IHR/PVS matrix (50’) + Coffee break (20’)
• Discussion – Plenary (30’)</td></tr>
<tr><td colspan="2">Expected outcomes of Session 3:
• Understanding that tools are available to explore capacities in each of the sectors.
• Understanding of the contribution of the veterinary sector to the IHR.
• Understanding of the bridges between the IHR MEF and the PVS Pathway.
• Identification of the technical areas to focus on during the next sessions.</td></tr>
<tr><td rowspan="2">11:20 - 12:40</td><td><u>Session 4: Crossroads - IHR MEF, JEE and PVS Pathway reports</u>
Participants will be divided into working groups by technical topic (surveillance, communication, coordination, etc) and will explore the improvement plans already proposed in the respective assessments (IHR annual reporting, JEE, PVS Evaluation, etc.), extract relevant sections and identify what can be synergized or improved jointly.</td></tr>
<tr><td>• Presentation and organization of the working group exercise (20’)
• Extract main gaps and recommendations from the PVS and IHR reports (including the JEE), in relation to gaps identified on the matrix (60’)</td></tr>
<tr><td colspan="2">Lunch (13:00-14:00)</td></tr>
<tr><td rowspan="2">14:00 - 14:30</td><td><u>Session 4 (continued)</u></td></tr>
<tr><td>• Extract main gaps and recommendations from the PVS and IHR reports (including the JEE), in relation to gaps identified on the matrix (continued, 30’)</td></tr>
<tr><td colspan="2">Expected outcomes of Session 4:
• Good understanding of the assessment reports, their purpose and their structure.
• Main gaps and recommendations from existing reports have been extracted.
• A common understanding of the effort needed starts to emerge.</td></tr>
<tr><td rowspan="2">14:30– 17:15</td><td><u>Session 5: Road planning</u>
Participants will use the results obtained from the case studies and from the assessment reports to develop a realistic and achievable road-map to improve the collaboration between the sectors.</td></tr>
<tr><td>• Presentation and organization of the working group exercise (15’)
• Identification of Activities (Working groups by technical topic) (150’)</td></tr>
<tr><td colspan="2">Expected outcomes of Session 5:
• Clear and achievable activities are identified to improve inter-sectoral collaboration between the two sectors for all technical areas selected.</td></tr>
<tr><td>17.15 – 19.00</td><td>Facilitators only: Compilation of results from Session 5 (drafting of the road-map) and preparation of Session 6</td></tr>
</table>

<table>
<tr><th colspan="2">DAY 3</th></tr>
<tr><td rowspan="2">9:00 - 12:30</td><td><u>Session 6: Fine-tuning the roadmap</u>
The objective of Session 6 is to have all participants contribute to all technical areas and to consolidate the joint-road map by making sure it is harmonized, concrete and achievable.</td></tr>
<tr><td>• Fine-tuning of the road-map: Objectives and filling out of Activity cards (90’)
Coffee break (15’)
• World Café (90’)
• Presentation of the prioritization vote (10’)
• Prioritization vote (during lunchtime)</td></tr>
<tr><td colspan="2">Expected outcomes of Session 6:
• Harmonized, concrete and achievable road-map.
• Timeline, focal points, needed support and indicators have been identified for each activity.
• The impact and the difficulty of implementation of proposed activities have been estimated.
• Buy-in and ownership of all participants who contributed to all areas of the road-map.
• Prioritization of the activities.</td></tr>
<tr><td colspan="2">Lunch (12:15-13:30)</td></tr>
<tr><td rowspan="2">13:30 - 15:30</td><td><u>Session 7: Way forward</u>
In the last session, representatives from the key Ministries take over the leadership and facilitation of the workshop to discuss with participant about the next steps and how the established roadmap will be implemented.
Linkages with other mandated plans such as the National Action Plan for Health Security are discussed. This is also where any need from the country can be addressed. This will depend greatly on the current status of the country in terms of IHR-MEF and on the level of One Health capacity.</td></tr>
<tr><td>• Results of the prioritization vote (15’)
• Integrating the action points into the IHR-MEF process (30’)
• Next steps (75’) (lead by Ministry representatives)</td></tr>
<tr><td colspan="2">Expected outcomes of Session 7:
• Linkages with NAPHS.
• Identification of immediate and practical next steps.
• Identification of opportunities for other components of the IHR-MEF.</td></tr>
<tr><td>15:30 - 16:30</td><td><u>Closing Session</u>
• Evaluation of the workshop (20’)
• Closing ceremony (40’)</td></tr>
<tr><td>16.30 – 17.00</td><td>Facilitators: Video interview of some participants</td></tr>
</table>

ANNEX 2: EXAMPLES OF CASE STUDY SCENARIOS

Venezuelan Equine Encephalitis

During the first week of May 2016, a cluster of 4 cases of neurological symptoms in horses is reported by local veterinarians of [Location] to the Veterinary Service. There is no evidence or reports of mortality in wild birds, but no surveillance system is in place. Three weeks later, human cases are detected. On June 1st, first laboratory results are obtained from equine samples, where it is confirmed that they are positive to Venezuelan Equine Encephalitis (EEV).

Rabies

A case of rabies which was confirmed in a dairy cow recently inseminated and regularly milked, generates panic in the population. A stray dog which was known to have bitten two cows and was behaving aggressively towards people was reported to have bitten some children in the same neighborhood. It was shot dead by Police in the outskirts of [Location] two days ago. The carcass of the dog was destroyed before the Veterinary authorities were able to take the head of the dog for confirmation of diagnosis.

Avian Influenza

Two persons were admitted at the [Location] Hospital with pneumonia. Laboratory testing by RT-PCR resulted positive for H7N9 subtype of avian influenza. One of the patients is a semi-commercial broiler producer who sells his birds three times a week at the local live bird market. The other patient reported having visited the same market 7 days prior to disease onset and having bought four quails.

Anthrax

9 people are showing identical anthrax like lesions reported in a district hospital close to a border post. One of these patients is a worker at the village's slaughterhouse.

Streptococcus suis

Private Vet reports unusual mortality among piglets in a commercial farm. Workers on the farm also show illness.

Undiagnosed Emerging Infectious Disease

An exporting country suspects that a shipment of piglet to [Location] was contaminated with Streptococcus suis and entered into the market.

Salmonellosis

90 people in the capital sought medical attention when they suffered high fever, nausea, diarrhea and severe abdominal pain, 12-36 hours after eating breakfast at a prominent hotel. Of these, 7 (5 children and 2 elderly) were hospitalized. All recovered within a week. The Managing Director of the hotel said that it sourced its eggs from a reputable supplier, and that the hotel stored its eggs according to food safety standards.

CCHF

One week prior to the Aïd celebrations six butchery workers from the District of [Location] who had been involved in the routine slaughter of sheep died having developed acute hemorrhagic symptoms. Another person from the same butchery was admitted to hospital in [Location] and was diagnosed as having contracted CCHF.

Brucellosis

During the last month three cows all belonging to a small-holder dairy farmer in [Location] aborted. At the time of the first two abortions the farmer did not bother to report the problem to his local veterinary officer as his farm was too far away from the District Veterinary Office. However, the third abortion took place a day before market day and he happened to be in town, where he met the District vet and he mentioned that 3 of his cows had recently aborted their calves. The veterinarian quickly went to the farm and carried out a Milk Ring Test on the three animals which had aborted and found them all to be positive for Brucellosis.

ANNEX 3: EVALUATION QUESTIONNAIRE

This questionnaire aims to collect your feedback and suggestions on the IHR-PVS National Bridging Workshop. The objective of WHO and OIE is to improve the quality of future events.

(Optional) Last name / first name: ………………………………………………………

Your sector: Human health ☐ Animal Health☐ Environment ☐ Other ☐

Your level: National ☐ Regional ☐ Local/district ☐ Other ☐

Scale: 1 = Not satisfied at all 2 = Not really satisfied 3 = Satisfied 4 = Fully satisfied

	**Satisfaction level**	**Comment**<br>If rated 1 or 2, please justify
**Overall experience**	1☐ 2☐ 3☐ 4☐	
**Content**<br>(Quality, relevance, technical-level)	1☐ 2☐ 3☐ 4☐	
**Format**<br>(Method, material, activities)	1☐ 2☐ 3☐ 4☐	
**Facilitators**<br>(Communication skills, technical expertise)	1☐ 2☐ 3☐ 4☐	
**Organization**<br>(Logistics, venue, assistance)	1☐ 2☐ 3☐ 4☐	

Scale: 1 = No impact at all 2 = Weak impact 3 = Significant impact 4 = Highest impact

	**Impact**	**Comment**<br>If rated 1 or 2, please justify
How would you rate the impact of this event on:		
**Your technical knowledge on the subject matter**	1☐ 2☐ 3☐ 4☐	
**The work of your department/unit**	1☐ 2☐ 3☐ 4☐	
**The collaboration between AH and PH in your country**	1☐ 2☐ 3☐ 4☐	

Would you recommend this workshop to other countries?

Not at all ☐ Likely not ☐ Probably ☐ Absolutely ☐

Evaluation of the sessions

1 = Not satisfied at all 2 = Not really satisfied 3 = Satisfied 4 = Fully satisfied		
Please rate only the sessions you have attended	**Content, Format and Usefulness of the session**	**Comment**
**Session 1:** Setting the scene	1☐ 2☐ 3☐ 4☐	
**Session 2:** Case studies and evaluation of collaboration	1☐ 2☐ 3☐ 4☐	
**Session 3:** IHR & PVS tools and mapping of gaps	1☐ 2☐ 3☐ 4☐	
**Session 4:** Compilation of gaps & recommendations from existing reports	1☐ 2☐ 3☐ 4☐	
**Session 5:** Brainstorm on joint activities	1☐ 2☐ 3☐ 4☐	
**Session 6:** Fine-tuning of the road-map, World café, Prioritization vote	1☐ 2☐ 3☐ 4☐	
**Session 7:** Way forward	1☐ 2☐ 3☐ 4☐	

- **In your view, what were the main strengths of this workshop?**

- **In your view, what were the main weaknesses of this workshop?**

FACILITATOR CHECK-LISTS

PREPARATION CHECK-LIST

ITEM	#	STATUS
**HQ / RO printing & procurement**		
Poster workshop A0	1	
Road-lane arrows	5	
A1 IHR-PVS matrix poster	5	
Pack of 45 technical cards	5	
Gap cards	75	
Recommendation cards	75	
Objective cards	25	
Activity cards	75	
Session 2 report sheets	5	
Handbook technical table	15	
Large standing IHR-PVS Matrix	1	
Black marker pens	10	
Black fine-pointed marker pens	20	
Blue round stickers (100pc)	3	
Red round stickers (100pc)	3	
White round sticker (100pc)	3	
Post-its packs (3 colors)	3	
Blue-tack (80pc)	4	
**In-country printing / procurement**		
Participant Handbook (color)	1 pax	
Case-study scenarios	15	
PVS country reports	15	
JEE or self-assessment reports	15	
Evaluation form	1 pax	
USB stick	1 pax	
Flip-chart	5	
Participant badge	1 pax	

END OF SESSION 1 CHECK-LIST

- ☐ Name, title and affiliation of the people that talked during the opening ceremony were captured
- ☐ Presentations of the Ministries were collected, as well as full name, title and affiliation of presenters
- ☐ Case study scenarios are validated by both Ministries
- ☐ Working groups are constituted
- ☐ Workshop poster is posted on a wall using the blue-tack
- ☐ Material is ready for Session 2 as per the check-list below

ITEM	#	STATUS
**Material for Session 2**		
List of participants for each group is printed	1 for each group	
Case study scenarios are printed	3 for each group	
Road-lane arrow posters	1 for each group	
Deck of 45 technical cards	1 for each group	
Black marker pen	2 for each group	
Blue tack	20 pcs for each group	
Report sheet	1 for each group	

END OF SESSION 2 CHECK-LIST

- ☐ Results of session 2 are captured in the Facilitator's Excel sheet
- ☐ Results are discussed, and technical groups are pre-identified by facilitators
- ☐ Session 2 report sheets have been collected and photocopied (5 copies of each)
- ☐ IHR-PVS Matrix is set-up
- ☐ Material is ready for Session 3 as per the check-list below

ITEM	#	STATUS
**Material for Session 3**		
Movie 3 on IHR	1	
Movie 4 on PVS	1	
Movie 5 on Bridging IHR and PVS	1	
A1 IHR-PVS matrix poster	1 for each group	
Copy of the 15 technical cards chosen by each group	1 for each group	
Blue-tack	20pc for each group	

END OF SESSION 3 CHECK-LIST

- ☐ Prepare the template on the flip chart for each group
- ☐ Material is ready for Session 4 as per the check-list below

ITEM	#	STATUS
**Material for Session 4**		
Flip-chart with template	1 per group	
JEE report (or self-evaluation)	3 per group	
PVS Evaluation (or Follow-up) report	3 per group	
Indicator tables for JEE	3 per group	
Indicator tables for PVS	3 per group	
Gap cards	12 per group	
Recommendation cards	12 per group	
Blue-tack	20pcs per group	
Fine-pointed marker pen	2 for each group	

Flipchart template for Session 4:

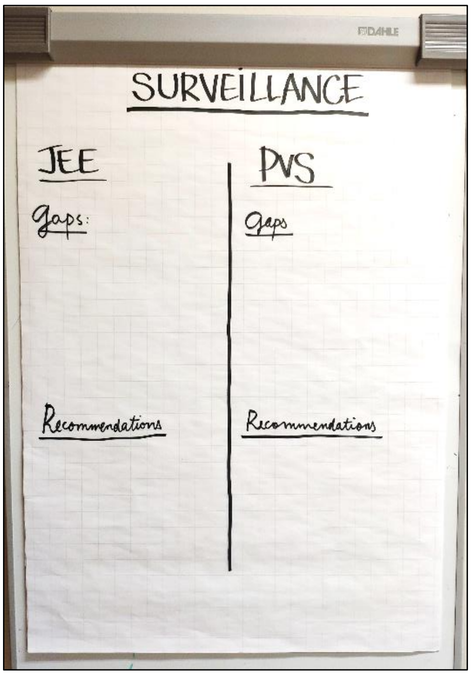

END OF SESSION 4 CHECK-LIST

- ☐ Prepare the template on the flip chart for each group
- ☐ Material is ready for Session 5 as per the check-list below

ITEM	#	STATUS
**Material for Session 5**		
Flip-chart with template	1 per group	
Fine-pointed marker pen	2 for each group	
Photocopy of the session 2 report sheets	1 copy of each disease for each group	
Copy of the red technical cards (used in session 2) relevant to each group	1-2 per group	

Flipchart template for Session 5 and 6

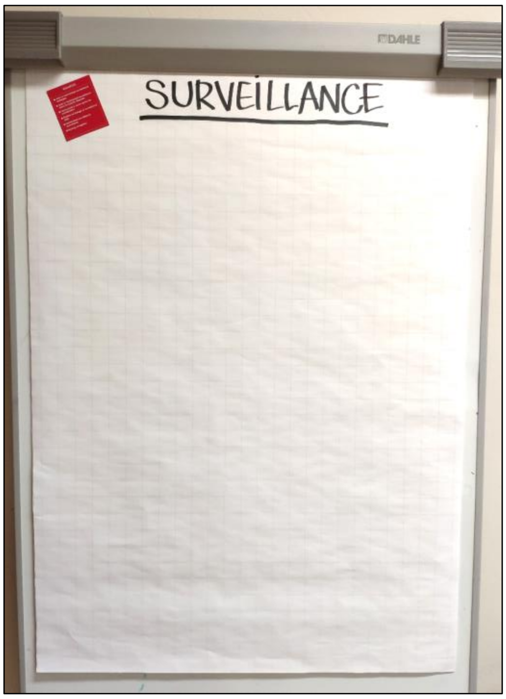

END OF SESSION 5 CHECK-LIST

- ☐ Review all activities with the facilitating team
- ☐ Leave yellow post-it notes for comments to be addressed by the group
- ☐ Leave red post-it notes for modifications that require a discussion with a facilitator
- ☐ Give a number to each panel for the world café
- ☐ A master USB key with all the material of the workshop is ready

ITEM	#	STATUS
**Material for Session 6**		
Flip-chart with template	1 per group	
Objective cards	3 per group	
Activity cards	10 per group	
Blue-tack	20pcs for each group	
Fine-pointed marker pen	2 for each group	
Red round stickers	30 for each group	
Blue round stickers	30 for each group	

END OF SESSION 6 CHECK-LIST

ITEM	#	STATUS
**Material for Session 7**		
Evaluation form	1 per participant	
USB stick with all the material	1 per participant	

END OF WORKSHOP CHECK-LIST

- ☐ All activities and objectives (including voting results) have been captured in the Excel Road-map template
- ☐ Feedback forms have been collected
- ☐ USB sticks were given to participants

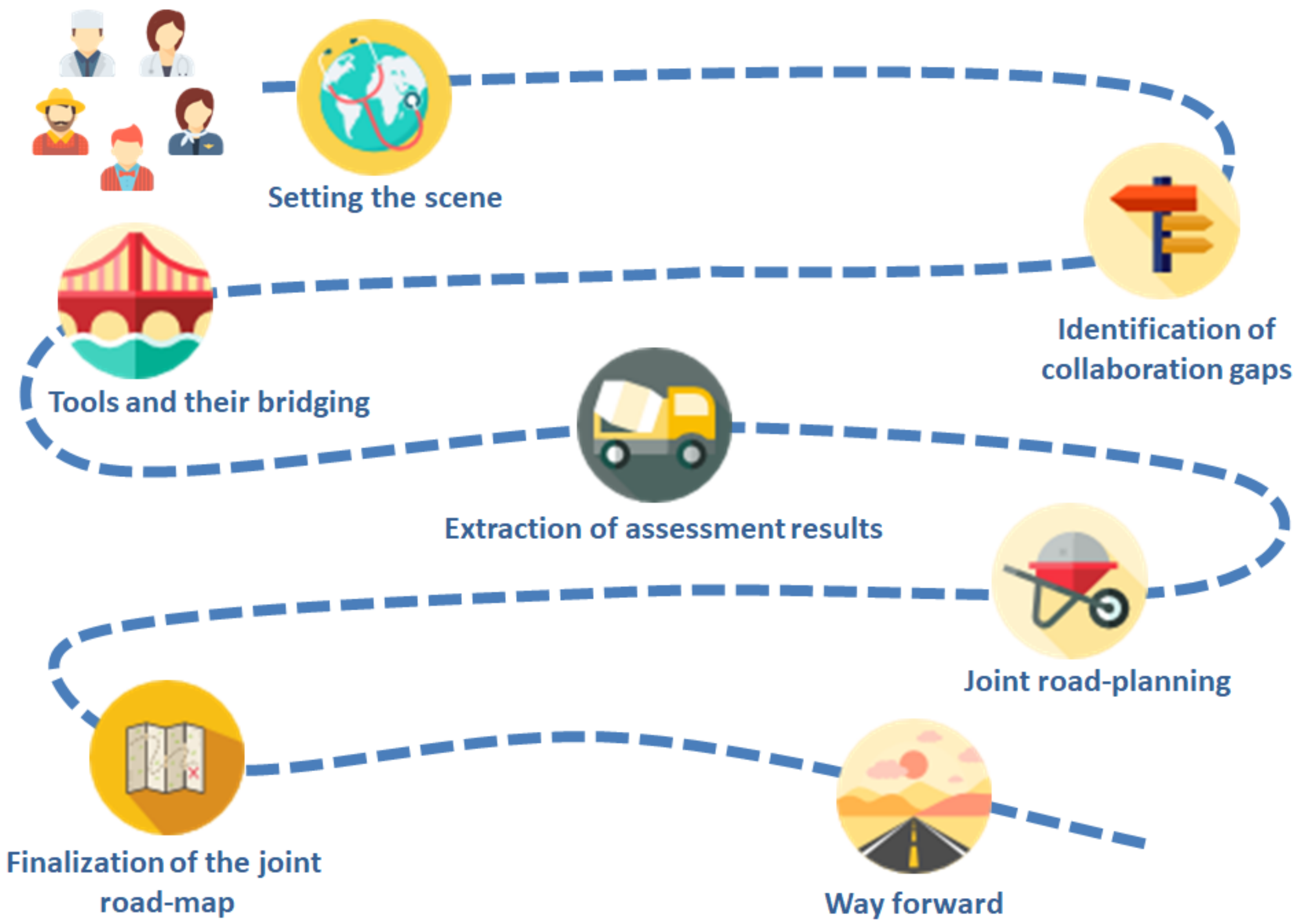
Setting the scene
Identification of collaboration gaps
Tools and their bridging
Extraction of assessment results
Joint road-planning
Finalization of the joint road-map
Way forward
