# Supplementary material for: IHR-PVS National Bridging Workshops, a tool to operationalize the collaboration between human and animal health while advancing sector-specific goals in countries
Source: PLoS One. 2021 Jun 1;16(6):e0245312. doi: 10.1371/journal.pone.0245312 (PMC8168895; doi:10.1371/journal.pone.0245312)
Supplement: S3 Appendix — (PDF) [file pone.0245312.s003.pdf]

NBW FACILITATOR – CHECK-LISTS KIT

PREPARATION CHECK-LIST

ITEM	#	STATUS
**HQ / RO printing & procurement**		
Poster workshop A0	1	
Road-lane arrows	5	
A1 IHR-PVS matrix poster	5	
Pack of 45 technical cards	5	
Gap cards	75	
Recommendation cards	75	
Objective cards	25	
Activity cards	75	
Session 2 report sheets	5	
Handbook technical table	15	
Large standing IHR-PVS Matrix	1	
Black marker pens	10	
Black fine-pointed marker pens	20	
Blue round stickers (100pc)	3	
Red round stickers (100pc)	3	
Green round sticker (100pc)	3	
Removable glue-stick	2	
Post-its packs (3 colors)	4	
Blue-tack (80pc)	4	
**In-country printing & procurement**		
Participant Handbook (color)	1 pax	
Case-study scenarios	15	
PVS country reports	15	
JEE or self-assessment reports	15	
Evaluation form	1 pax	
USB stick	1 pax	
Flip-chart	5	

PRE-WORKSHOP MEETING

One day before the workshop, a pre-workshop meeting should be organized with:
-Representatives from WHO (country and regional office)
-Representatives from OIE
-Representatives from line Ministries of the country

Check-list for the pre-workshop meeting:

- ☐ Method and process of the workshop was presented to the line Ministries (use video)
- ☐ Go over and validate the agenda
- ☐ Organize the opening ceremony (who will speak, in what order)
- ☐ Ensure that the minimum number of participants will be present (minimum 25 from each Ministry)
- ☐ Ensure that presentations from the Ministries are ready. Stress that the presentations should focus on the structure of each services, their key activities in terms of One Health, and their challenges. They should not be a lecture on One Health concept, or the PVS Pathway or the IHR.
- ☐ Discuss and validate the case studies
- ☐ Tell Ministry representatives that they will need to come up with a detailed list of working groups for each disease by lunchtime of day 1
- ☐ Tell Ministries that they will be taking the full leadership of session 7 on the way forward. This can include a presentation/discussion of how results will be included in other mandated plans (ex: NAPHS, One Health strategic plans, etc). The aim is to show that the Ministries take full ownership of the road-map and leadership of its implementation
- ☐ Talk about the workshop report that will be drafted by WHO and OIE and reviewed by line Ministries
- ☐ Ask if Ministries have any other needs to address during the workshop
- ☐ Address any question or concern raised by the Ministries

The day before the workshop:

- ☐ Use the **Preparation checklist** to ensure that all the material is ready
- ☐ Visit the meeting rooms and ensure that all facilities and equipment are available (projector, screen, microphones, audio system for both microphone and computer).
- ☐ Ensure that **both** OIE and WHO logos are on the agenda and the workshop banner and at the same level of importance

END OF SESSION 1 CHECK-LIST

- ☐ Name, title and affiliation of the people that talked during the opening ceremony were captured
- ☐ Presentations of the Ministries were collected, as well as full name, title and affiliation or presenters
- ☐ Case study scenarios are validated by both Ministries
- ☐ Working groups are constituted
- ☐ Material is ready for Session 2 as per the check-list below

ITEM	#	STATUS
**Material for Session 2**		
List of participants for each group is printed	1 for each group	
Case study scenarios are printed	3 for each group	
Road-lane arrow posters	1 for each group	
Deck of 45 technical cards	1 for each group	
Black marker pen	2 for each group	
Removable glue stick or blue tack	1 for each group	
Report sheet	1 for each group	

END OF SESSION 2 CHECK-LIST

- ☐ Results of session 2 are captured in the Facilitator's Excel sheet
- ☐ Results are discussed and technical groups are pre-identified by facilitators
- ☐ Session 2 report sheets have been collected and photocopied (5 copies of each)
- ☐ IHR-PVS Matrix is set-up
- ☐ Material is ready for Session 3 as per the check-list below

ITEM	#	STATUS
**Material for Session 3**		
Movie on IHR	1	
Movie on PVS	1	
Movie on Bridging IHR and PVS	1	
A1 IHR-PVS matrix poster	1 for each group	
The 15 technical cards chosen by each group	1 for each group	
Blue-tack	20pcs per group	

END OF SESSION 3 CHECK-LIST

- ☐ Prepare the template on the flip chart for each group
- ☐ Material is ready for Session 4 as per the check-list below

ITEM	#	STATUS
**Material for Session 4**		
Flip-chart with template	1 per group	
JEE report (or self-evaluation)	3 per group	
PVS Evaluation (or Follow-up) report	3 per group	
Indicator tables for JEE	3 per group	
Indicator tables for PVS	3 per group	
Gap cards	12 per group	
Recommendation cards	12 per group	
Blue-tack	20pcs per group	
Fine-pointed marker pen	2 per group	

END OF SESSION 4 CHECK-LIST

- ☐ Prepare the template on the flip chart for each group
- ☐ Material is ready for Session 5 as per the check-list below

ITEM	#	STATUS
**Material for Session 5**		
Flip-chart with template	1 per group	
Fine-pointed marker pen	2 for each group	
Photocopy of the session 2 report sheets	1 copy of each disease for each group	
Copy of the technical cards relevant to each group	1 per group	

END OF SESSION 5 CHECK-LIST

- ☐ Review all activities with the facilitating team
- ☐ Leave yellow post-it notes for comments to be addressed by the group
- ☐ Leave red post-it notes for modifications that require a discussion with a facilitator
- ☐ Give a number to each panel for the world café
- ☐ A master USB key with all the material of the workshop is ready

ITEM	#	STATUS
**Material for Session 6**		
Flip-chart with template	1 per group	
Objective cards	3 per group	
Activity cards	10 per group	
Blue-tack	20pcs per group	
Fine-pointed marker pens	2 per group	
Red round stickers	30pcs per group	
Blue round stickers	30pcs per group	
Green round stickers for prioritization	5pcs per participant	

END OF SESSION 6 CHECK-LIST

- ☐ Master USB key has been copied
- ☐ Material is ready for Session 7:

ITEM	#	STATUS
**Material for Session 7**		
Evaluation form	1 per participant	
USB stick with all the material	1 per participant	

END OF WORKSHOP CHECK-LIST

- ☐ All activities and objectives (incl. voting results) have been captured in the Excel roadmap template
- ☐ Feedback forms have been collected
- ☐ USB sticks have been given to participants
