# Supplementary material for: IHR-PVS National Bridging Workshops, a tool to operationalize the collaboration between human and animal health while advancing sector-specific goals in countries
Source: PLoS One. 2021 Jun 1;16(6):e0245312. doi: 10.1371/journal.pone.0245312 (PMC8168895; doi:10.1371/journal.pone.0245312)
Supplement: S4 Appendix — (PDF) [file pone.0245312.s004.pdf]

Q3. What is the role of WHO and OIE?

WHO and OIE experts act as **workshop facilitators.** For the workshop to be a success, it is critical that the organising Ministries take **ownership** of it.
As such, the role of WHO and OIE facilitators is to present and explain the methodology in order to create a conducive environment for the identification of gaps, needs and corrective measures. It is **not a training,** and it is not WHO or OIE's role to bring solutions. The **solutions are home-grown**, identified and fine-tuned by participants throughout the different exercises.
WHO and OIE handle all the logistical aspects of the workshop preparation (room rental, equipment, material, facilitators, meals, etc.).

Q4. Who should participate?

A strict **minimum of 50 participants** is needed, although 60 or more is recommended. An **ideal distribution** of participants would be:

- **30 representatives for animal health (1/3 national, 1/3 regional, 1/3 local)**
- **30 representatives for human health (1/3 national, 1/3 regional, 1/3 local)**
- **10 representatives from other sectors** (wildlife, environment, police, media, etc.).

Because of the level of interactivity of the workshop it is important to select **dynamic participants** who **actively contribute** to the discussions and exercises. Due to the way the sessions are interconnected, it is important that all participants commit to the three days of the workshop.

59
Average number of national participants per workshop (from 20 NBWs)

98%
Average participant satisfaction level (from 20 NBWs)

Q5. Who should be invited as observers?

It is up to the organising Ministries to decide who they wish to invite as observers. It is highly recommended to invite any **organisation, institution, donor, NGO, academia** or other partner involved in national activities related to human or animal health and who could either **contribute to the development** of the roadmap, or who could possibly assist, either technically or financially, in the **follow-ups** and in the **implementation** of the activities that will be defined in the roadmap.

Q6. I am a development agency and wish to support NBWs, what should I do?

The success of the methodology has triggered a lot of interest from many countries. The list of country candidates for NBWs is growing fast and funding opportunities become the limiting factor. If your agency is interested in supporting the implementation of a NBW in a specific country, please contact the focal points in WHO and OIE *(see Q8)*.

Q7. I am a country representative and wish to plan an NBW in my country, what should I do?

Implementation of an IHR-PVS NBW is a voluntary process. If you are interested in running a workshop in your country, please contact either the regional OIE representation or your WHO country office, to submit a request.
It is better if the country has undergone a PVS Evaluation or PVS Follow-up in the recent years. The timing of an NBW is also ideal when it is implemented after a Joint External Evaluation (JEE) has been conducted and before a National Action Plan is developed. However, the flexibility of the tool ensures that it can be successful even in the absence of any JEE.

100%
Of participants recommend this workshop to other countries (from 20 NBWs)

Q8. How can I get more information?

Link	Description
www.bit.ly/NBWVideo	5-minute introduction to NBWs
www.bit.ly/NBWMethod	4-minute video on the method of the 7 sessions
www.bit.ly/NBWTanzania	3-minute video of the NBW organized in Tanzania
www.bit.ly/NBWManual	Facilitator's Manual (PDF)
www.bit.ly/NBWParticipant	Participant Handbook (PDF)

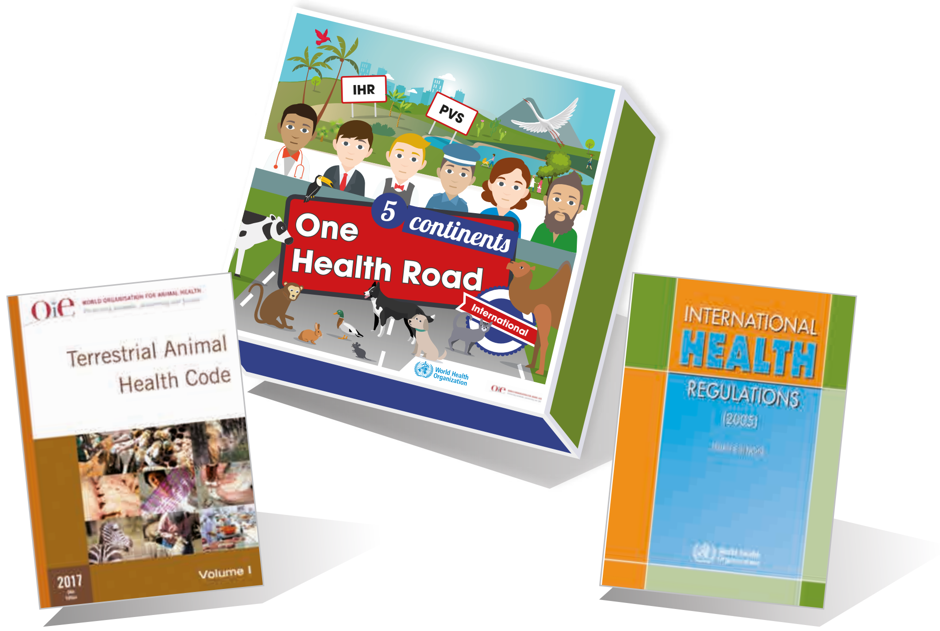

Contact points

WHO HQ	Dr Stephane de la ROCQUE (delarocques@who.int)
OIE HQ	Dr François CAYA (f.caya@oie.int)

WORLD ORGANISATION FOR ANIMAL HEALTH

World Health Organization

© World Health Organization 2019. Some rights reserved. This work is available under the CC BY-NC-SA 3.0 IGO licence. Design: crayonbleu.com - France

FACT SHEET

IHR-PVS NATIONAL BRIDGING WORKSHOP

A three-day interactive event to bring human health and animal health actors to work together

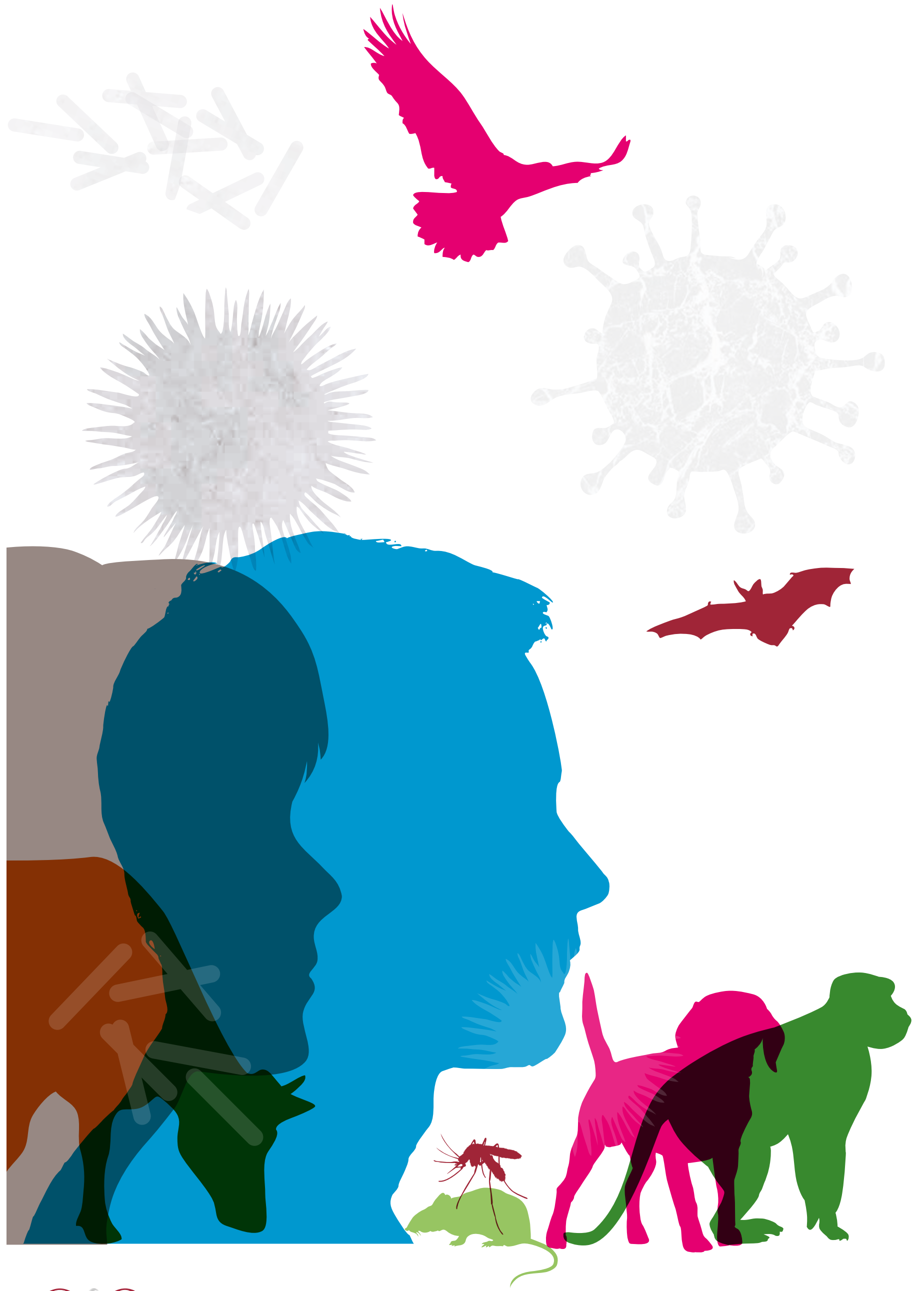

WORLD ORGANISATION FOR ANIMAL HEALTH

World Health Organization

Q1. What is an IHR-PVS National Bridging Workshop (NBW)?

The IHR-PVS NBW is a **three-day workshop** which brings together 60 to 90 stakeholders from the **animal health** and the **human health** services of the country to improve their **collaboration** at the human-animal interface. Representatives of other relevant sectors (environment, wildlife, media, police etc.) often participate.

A 5-minute video explaining the concept of these NBWs can be viewed at the following link: www.bit.ly/NBWVideo.

Q2. What is the approach used during this workshop?

The workshop is very interactive with the whole process being built around **group exercises and discussions**.
The workshop is divided into **seven sessions** and follows a **step-by-step approach** where the results of each session feed into the next session:

KEY OUTPUTS OF THE NBW

A diagnosis of current **strengths** and **weaknesses** in the **collaboration** between animal and human health services for 15 technical areas that are key for the prevention, detection and response to health events at the human-animal interface (zoonotic diseases, food safety and AMR).

A harmonized, detailed and realistic **joint roadmap** of activities that the country will implement to improve the collaboration between the two sectors.

JOINT TECHNICAL AREAS ADDRESSED IN A NBW

- Coordination at high Level
- Coordination at local Level
- Coordination at technical Level
- Legislation / Regulation
- Finance
- Emergency funding
- Communication with media
- Communication with stakeholders
- Field investigation
- Response operations
- Risk assessment
- Joint surveillance
- Laboratory
- Education and training
- Human resources

SESSION 1

Setting the scene (One Health concept, examples of successful interactions).

SESSION 2

Participants are divided into working groups and each group is given a different simulation scenario. The objective is to identify **strengths** and **weaknesses** in the current collaboration for **15 key technical areas.**

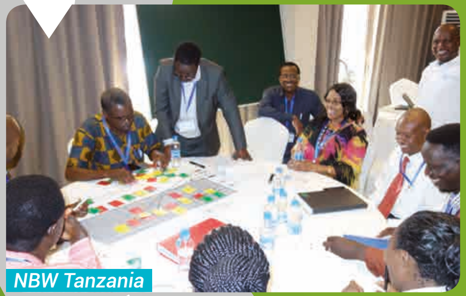
NBW Tanzania

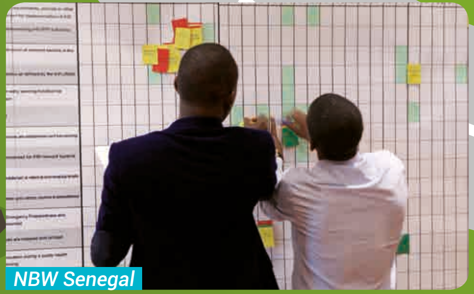
NBW Senegal

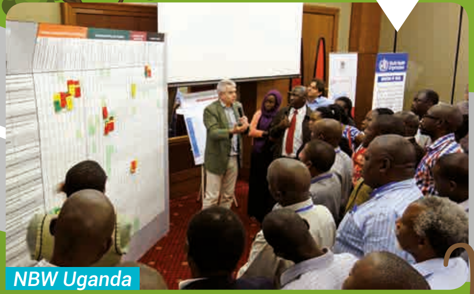
NBW Uganda

The tools (WHO's IHR MEF and OIE's PVS Pathway) are presented and the strengths and weaknesses identified in session 2 are mapped on the **IHR-PVS matrix.**

SESSION 3

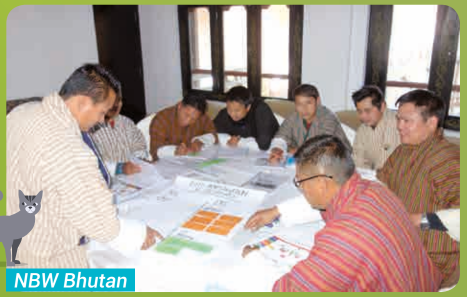
NBW Bhutan

Using results from session 3, five technical working groups are made. Participants use the reports of the assessments already conducted by both sectors to extract the information relevant for their technical area.

SESSION 4

SESSION 5

Each group identifies **key joint objectives and activities** to strengthen the collaboration between the two sectors.

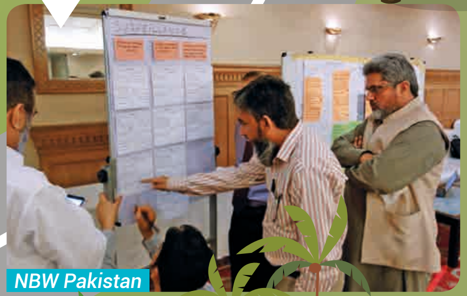
NBW Pakistan

A world café exercise and a prioritization vote are conducted to finalize the **joint roadmap.**

SESSION 6

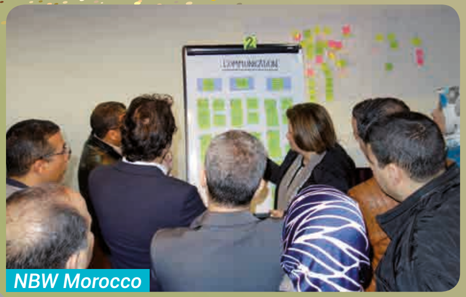
NBW Morocco

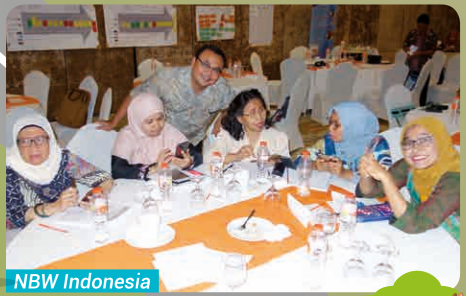
NBW Indonesia

Discussion on the way forward and on the implementation of the joint roadmap.

SESSION 7
