# Supplementary material for: IHR-PVS National Bridging Workshops, a tool to operationalize the collaboration between human and animal health while advancing sector-specific goals in countries
Source: PLoS One. 2021 Jun 1;16(6):e0245312. doi: 10.1371/journal.pone.0245312 (PMC8168895; doi:10.1371/journal.pone.0245312)
Supplement: Attachment — Submitted filename: PONE Submission - Response to Reviewers.docx [file pone.0245312.s006.docx]

**Journal Requirements:**

1. Please ensure that your manuscript meets PLOS ONE's style requirements, including those for file naming. The PLOS ONE style templates can be found at

https://journals.plos.org/plosone/s/file?id=wjVg/PLOSOne_formatting_sample_main_body.pdf and

<https://journals.plos.org/plosone/s/file?id=ba62/PLOSOne_formatting_sample_title_authors_affiliations.pdf>

Response from authors: after revision of the style requirements, the following corrections were brought to the manuscript:

- Removal of Figures from the manuscript
- Relocation of the legends after the tables for Table 2 and Table 3
- Relocation of the ‘Supporting Information’ section after the ‘References’ section

The manuscript now meets PLOS ONE’s style requirements as detailed in the templates provided.

2. Thank you for stating the following in the Acknowledgments Section of your manuscript:

"This work has been supported by many funders including the United States Defense Threat
 Reduction Agency (US-DTRA), the Global Partnership Program (GPP), the EU Commission's
Directorate-General for International Cooperation and 475 Development (DG DEVCO), the Russian
Federation and the World Bank, among others."

We note that you have provided funding information that is not currently declared in your Funding Statement. However, funding information should not appear in the Acknowledgments section or other areas of your manuscript. We will only publish funding information present in the Funding Statement section of the online submission form.

Please remove any funding-related text from the manuscript and let us know how you would like to update your Funding Statement. Currently, your Funding Statement reads as follows:

"The authors received no specific funding for this work"

Please include your amended statements within your cover letter; we will change the online submission form on your behalf.

Response from authors:

- The funding-related paragraph was removed from the Acknowledgments section.
- Our amended statement was added in the cover letter: “The organization of IHR-PVS National Bridging Workshops in countries was supported by many funders including the United States Defense Threat Reduction Agency (US-DTRA), the Global Partnership Program (GPP), the EU Commission's Directorate-General for International Cooperation and Development (DG DEVCO), the Russian Federation and the World Bank, among others.”

3. Please remove your figures from within your manuscript file, leaving only the individual TIFF/EPS image files, uploaded separately.  These will be automatically included in the reviewers’ PDF.

Response from authors: All figures have been removed from the manuscript accordingly.

 4. We note that Figure 4 in your submission contain map images which may be copyrighted. All PLOS content is published under the Creative Commons Attribution License (CC BY 4.0), which means that the manuscript, images, and Supporting Information files will be freely available online, and any third party is permitted to access, download, copy, distribute, and use these materials in any way, even commercially, with proper attribution. For these reasons, we cannot publish previously copyrighted maps or satellite images created using proprietary data, such as Google software (Google Maps, Street View, and Earth). For more information, see our copyright guidelines: http://journals.plos.org/plosone/s/licenses-and-copyright.

We require you to either (1) present written permission from the copyright holder to publish these figures specifically under the CC BY 4.0 license, or (2) remove the figures from your submission.

 Response from authors: The map (Figure 4) was replaced by a table (Table 4) for enhanced clarity and to avoid any potential copyright issue.

**Comments from Reviewers:**

**Reviewer #1**

Reviewer #1: The enjoyed reading this article unveiling the role of National Bridging Workshops in operationalizing the collaboration between human and animal health sectors. The authors have highlighted the importance of integration of sector-specific and collaborative goals. The article can be considered for publication in the PLOS ONE. I have the following minor comments.
1. Introduction: Authors may consider revising the line 87-92.
2. Results: Authors should provide a summary of the key finding and significant features of the tool before getting into the detailed description. It is difficult to get the salient outcomes of the study in its current form.
3. Fig 4. Please indicate what different colours are referring to. I would suggest adding country names as well which make it easier to understand for the readers with different parts of the world.
4. The discussion could have been improved by incorporating major outcomes and its relevant literature from similar efforts/tools developed in the past.
5. Currently, methods, results and discussion – all the sessions are amalgamated and twisting around.
6. Conclusion: No comment.

Response from authors:

1. As suggested, lines 87-92 in the introduction have been fully rewritten and expanded upon for enhanced clarity (lines 93-98 of the track changes version).
2. The paragraph summarizing the 5 key lessons learned from the development of the tool was added in the Results section (lines 264-272) right before the detailed description: “Key lessons learned from the two phases of iterative development of the tool include (i) the need to have a shared understanding of sector-specific assessments such as IHR and PVS and how they contribute to collaborative advantages, (ii) the need to have representatives from different levels (national, sub-national, local) to jointly share the current status of collaboration and discuss how to operationalize shared outputs; (iii) the need for stakeholders to engage as early as possible in scenario-based exercises, so that the conceptualization of joint activities is facilitated and gaps can easily be identified and discussed; (iv) the importance of having the two sectors develop and commit to a joint, realistic and operational roadmap to improve their collaboration; and (v) a well-structured approach and robust facilitation are required for these events.”
3. The map (Figure 4) was replaced by a table (Table 4) displaying all the country names for clearer understanding.
4. We agree with the reviewer statements and have clarified and highlighted the following key outcomes in lines 389-391 of the version with track changes: The NBW is a novel tool which bridges internationally accepted framework (IHR and PVS) and tools from the two sectors to allow for improved collaboration while supporting sector-specific needs. It is the first tool that aims to do this and as such, no similar effort or tools was found in the literature for comparison.
5. Method: The authors have made every attempt to clarify the structure and flow of the paper to walk to the reader through a linear progression of methods, results and discussion. The method section now describes the iterative development of the process of NBWs, which of course was an iterative methodology of improvement. Once the full method and material for the NBWs were finalized, the process was divided into 7 distinct sessions. We edited line 254 to better clarify this: “The final process of the NBW was split into seven sessions (Table 2) over the course of a three-day in-person workshop”.

Result: We made some formatting changes to better separate the different sessions and make them more visible. Each session has its own paragraph to avoid any blending and to make sure each is distinctive. In addition, we moved the introduction of key findings from the methods to results as was suggested under point 2, see lines 285-293.

Discussion: The discussion points address the NBW event as a whole and are not session-specific. We discuss the fact that it is the first event of this type and then discuss what from our experience were the key success factors: (i) high-level engagement and country ownership, (ii) participant representation, (iii) interactive and participatory approach with robust facilitation and (iv) linkages with IHR and PVS sector-specific goals. These four points are addressed in further details in the manuscript but they are generic to the whole workshop and not specific to any session. To avoid any confusion, we removed any reference to a session number, apart from the paragraph detailing the role of country officials in the last session of the workshop as it is an important example to discuss point (i). As recommended in reviewer point 4, we added additional language to highlight the key outcome in lines 438-440.

**Reviewer #2**

Reviewer #2: The One Health approach is often visualized with three key actors: human health, animal health and environmental health. The authors explains in the emanuscript an endeavour for one health program across nations through a series of six phased pilots, the IHR-PVS National Bridging Workshop (NBW) method was developed and refined. The NBW process gathers human and animal health stakeholders and follows seven sessions, scheduled across three days. The outputs from each session build towards the next one, following a structured process that goes from gap identification to joint planning of corrective measures. These efforts helped identify the strength and gaps in country's preparedness. The NBW process allows human and animal health sector representatives to jointly identify actions that support collaboration while advancing evaluation goals identified through the IHR-MEF and the OIE PVS Pathway. These paper elaborates how an international effort was used counter One health issues and program to address the gaps was launched. The authors should carry more such programs in regard to One Health.

Response from authors: No specific revision was requested by Reviewer #2.
